# A systems serology approach to identifying key antibody correlates of protection from cerebral malaria in Malawian children

**DOI:** 10.1186/s12916-024-03604-8

**Published:** 2024-09-12

**Authors:** Isobel S. Walker, Saber Dini, Elizabeth H. Aitken, Timon Damelang, Wina Hasang, Agersew Alemu, Anja T. R. Jensen, Janavi S. Rambhatla, D. Herbert Opi, Michael F. Duffy, Eizo Takashima, Visopo Harawa, Takafumi Tsuboi, Julie A. Simpson, Wilson Mandala, Terrie E. Taylor, Karl B. Seydel, Amy W. Chung, Stephen J. Rogerson

**Affiliations:** 1 Department of Medicine, The University of Melbourne, The Peter Doherty Institute for Infection and Immunity, Melbourne, VIC 3000 Australia; 2 Centre for Epidemiology and Biostatistics, Melbourne School of Population and Global Health, The University of Melbourne, Parkville, VIC 3010 Australia; 3 Department of Infectious Diseases, The Peter Doherty Institute for Infection and Immunity, Melbourne, VIC 3000 Australia; 4 Department of Microbiology and Immunology, The Peter Doherty Institute for Infection and Immunity, Melbourne, VIC 3000 Australia; 5 Centre for Medical Parasitology, Department of Immunology and Microbiology, Faculty of Health and Medical Sciences, University of Copenhagen, Copenhagen, Denmark; 6 Burnet Institute, 85 Commercial Road, Melbourne, VIC 3004 Australia; 7 Department of Immunology, Monash University, Melbourne, VIC Australia; 8 Division of Malaria Research, Proteo-Science Center, Ehime University, Matsuyama, Japan; 9 Malawi-Liverpool-Wellcome Trust Clinical Research Programme, Blantyre, Malawi; 10 Division of Cell-Free Sciences, Proteo-Science Center, Ehime University, Matsuyama, Japan; 11 Academy of Medical Sciences, Malawi University of Science and Technology, Thyolo, Malawi; 12 Blantyre Malaria Project, Kamuzu University of Health Sciences, Blantyre, Malawi; 13 College of Osteopathic Medicine, Michigan State University, East Lansing, USA

**Keywords:** *Plasmodium falciparum*, Malawi, Antibody, Immunity, Africa

## Abstract

**Background:**

*Plasmodium falciparum* erythrocyte membrane protein 1 (PfEMP1) proteins are expressed on the surface of infected erythrocytes, mediating parasite sequestration in the vasculature. PfEMP1 is a major target of protective antibodies, but the features of the antibody response are poorly defined.

**Methods:**

In Malawian children with cerebral or uncomplicated malaria, we characterized the antibody response to 39 recombinant PfEMP1 Duffy binding like (DBL) domains or cysteine-rich interdomain regions (CIDRs) in detail, including measures of antibody classes, subclasses, and engagement with Fcγ receptors and complement. Using elastic net regularized logistic regression, we identified a combination of seven antibody targets and Fc features that best distinguished between children with cerebral and uncomplicated malaria. To confirm the role of the selected targets and Fc features, we measured antibody-dependent neutrophil and THP-1 cell phagocytosis of intercellular adhesion molecule-1 (ICAM-1) and endothelial protein C (EPCR) co-binding infected erythrocytes.

**Results:**

The selected features distinguished between children with cerebral and uncomplicated malaria with 87% accuracy (median, 80–96% interquartile range) and included antibody to well-characterized DBLβ3 domains and a less well-characterized CIDRγ12 domain. The abilities of antibodies to engage C1q and FcγRIIIb, rather than levels of IgG, correlated with protection. In line with a role of FcγRIIIb binding antibodies to DBLβ3 domains, antibody-dependent neutrophil phagocytosis of ICAM-1 and EPCR co-binding IE was higher in uncomplicated malaria (15% median, 8–38% interquartile range) compared to cerebral malaria (7%, 30–15%, *p* < 0.001).

**Conclusions:**

Antibodies associated with protection from cerebral malaria target a subset of PfEMP1 domains. The Fc features of protective antibody response include engagement of FcγRIIIb and C1q, and ability to induce antibody-dependent neutrophil phagocytosis of infected erythrocytes. Identifying the targets and Fc features of protective immunity could facilitate the development of PfEMP1-based therapeutics for cerebral malaria.

**Supplementary Information:**

The online version contains supplementary material available at 10.1186/s12916-024-03604-8.

## Background

There are more than 200 million cases of malaria annually that result in over half a million deaths, predominantly of children under 5 years of age [1]. Cerebral malaria is a severe and lethal manifestation of *Plasmodium falciparum* malaria that is characterized by impaired consciousness and predominantly occurs in children or adults who lack protective immunity. The pathogenesis of cerebral malaria is complex and only partially understood; however, a central component is the adhesion of parasite-infected erythrocytes (IE) to blood vessel endothelial cells in the brain, resulting in sequestration of IE in the cerebral microvasculature [2]. In the majority of cases, malaria illness is uncomplicated, with fever and non-specific symptoms including headache, myalgia, and chills, and children do not progress to cerebral malaria.

Adhesion of IE to endothelial cells is mediated by *Plasmodium falciparum* erythrocyte membrane protein 1 (PfEMP1) variant surface antigens expressed on the IE surface. PfEMP1 are encoded by *var* genes, of which there are ~ 60 variants per parasite genome, with one variant expressed at a time. The majority of *var* genes can be classified as group A, B, or C based on their upstream promoter sequence (UPS A, B, or C) and chromosomal location, and encode for PfEMP1s with a combination of two to ten Duffy binding like (DBL) domains and cysteine-rich interdomain regions (CIDRs), some of which are frequently found in tandem arrangements, known as domain cassettes (DCs) [3]. Groups B/A and C/B are intermediates of two groups (A and B or C and B), and group E consists of a unique pregnancy specific *var* gene, *var2csa*. DBL and CIDR domains are further classified into multiple types (DBL α, β, γ, δ, ε, ζ and x, and CIDR α, β and γ) and subtypes (denoted by numbers, e.g., CIDRα1.1) [3]. Representative PfEMP1 structures are illustrated in Additional file 1: Fig. S1. Despite the enormous diversity in *var* genes, previous studies have identified PfEMP1 domains that are more frequently expressed in different clinical manifestations of malaria, and some of these PfEMP1 domains are known to bind to particular endothelial cell receptors. Generally, group A *var* genes are associated with severe malaria, whereas group B and C *var* genes are associated with uncomplicated malaria. PfEMP1 with CIDRα1 domains that bind to endothelial protein C receptor (EPCR), including those within DC8, have been associated with severe and cerebral malaria [4, 5]. CIDRα1 domains are sometimes found adjacent to a DBLβ domain that binds to intercellular adhesion molecule 1 (ICAM-1), including in DC4 and DC13, enabling dual receptor binding of IEs to brain endothelial cells [6]. Dual binding PfEMP1 can be predicted by the presence of a short sequence of amino acids in group A and some group B/A DBLβ domains, referred to as DBLβ_motif_, and have been associated with cerebral malaria [7, 8].

PfEMP1 is the main target of antibodies on the surface of the IE and antibodies targeting PfEMP1 or recombinant PfEMP1 domains have been associated with protection from severe malaria and uncomplicated malaria in multiple studies [9–11], including protection from uncomplicated malaria associated with antibodies to a DBLβ domain [12], to “group 2” DBLα domains, and antibody to a CIDRγ3 domain [13, 14]. However, there does not appear to be a single antigen variant that is associated with cerebral malaria in all cases, and given the high diversity of PfEMP1, it is likely that individuals would have to acquire antibodies to a combination of PfEMP1 target antigens to be protected. Previous studies have shown that antibodies targeting PfEMP1 have diverse functions, including inhibition of adhesion to endothelial cells, and promoting phagocytosis by monocytes [15] and killing of IEs by neutrophils [16], and by natural killer cell cytotoxicity [17]. However, due to the high diversity of PfEMP1, most studies have focused on a small number of domains or parasite lines and have employed a small number of functional immunoassays. Systems serology involves characterizing multiple antibody Fab targets and Fc features, followed by machine learning to identify the most relevant antibody responses [18]. We have recently used this approach to identify antibody responses that best correlate with protection from placental malaria [19].

This study aimed to characterize the antibody responses at the time of hospital presentation in Malawian children with cerebral and uncomplicated malaria and to identify a combination of antibody features that could differentiate between the two groups. We measured 11 antibody Fc features targeting 39 PfEMP1 domains that have previously been associated with severe or uncomplicated malaria [20, 21] and used machine learning to select a combination of antibody targets and Fc features that could best discriminate between cerebral and uncomplicated malaria. These results provide important new insights into the development of protective antibody immunity against cerebral malaria.

## Methods

### Clinical samples

Study participants were Malawian children presenting with cerebral or uncomplicated malaria to Queen Elizabeth Central Hospital, Blantyre, Malawi, over three malaria seasons (2015–2017) [22]. Malaria was diagnosed by light microscopy or rapid diagnostic test accompanied by fever > 37.5 °C. Participants were classified as having cerebral malaria based on a Blantyre coma score (BCS) of ≤ 2, or as having uncomplicated malaria based on normal consciousness and a BCS of 5 [23]. Participants were included if they were aged between 6 months and 12 years and were excluded if they tested positive to HIV, had recent history of non-malaria illness, or appeared severely malnourished. Venous blood samples were collected at enrollment. Plasma was separated and stored at − 80 °C. To minimize differences in prior exposure to malaria, we matched individuals with cerebral and uncomplicated malaria based on village of residency and rural or urban environment. Five samples were further removed due to inadequate volumes to complete all assays. Plasma from 10 adults from a non-malaria endemic area (Melbourne, Australia) were included in each assay as negative controls.

### Recombinant proteins

The study included recombinant proteins previously associated with severe, cerebral, or uncomplicated malaria (see Additional file 1: Table 1). This included 28 PfEMP1 domains derived from *var* sequences that were upregulated in severe or uncomplicated malaria in Indonesian adults and children (coded as SM or UM, respectively) [10] and 11 PfEMP1 DBLβ domains associated with ICAM-1 or non-ICAM-1 binding PfEMP1 [21, 24, 25]. We included four merozoite antigens, merozoite surface protein-2 (MSP2), MSP3, erythrocyte binding antigen-175 (EBA175-RIII-B), and apical membrane antigen-1 (AMA1), and a sporozoite antigen, circumsporozoite protein (CSP), as markers of prior exposure. Tetanus toxoid antigen was used as a positive control and an antigen-free bead region was used as a negative control (see Additional file 1: Table 2).

### Multiplex immunoassay

A multiplex immunoassay was used to assess antibody reactivity and antigen-specific antibody features to the selected recombinant PfEMP1 domain antigens, as previously described in detail [26]. Recombinant proteins were coupled to Bio-Plex magnetic carboxylated beads (Bio-Rad, Hercules, CA, USA) as per the manufacturer’s instructions. For each assay, protein coupled beads were combined to a final concentration of 20 beads/µL per bead region in 1% bovine serum albumin in phosphate buffered saline (BSA/PBS). Combined beads were incubated in a 96-well plate (Corning, Corning, NY, USA) with participant plasma, diluted 1:50 in PBS, overnight at 4 °C on a plate shaker. Beads were washed with 1% BSA/PBS and incubated with detector antibody. We compared IgG responses to antigens probed in single format to multiplex array format, to confirm there was minimal antibody competition or interaction between antigens (see Additional file 1: Fig. S2). To detect IgG, IgG1, IgG2, IgG3, and IgG4, beads were incubated with phycoerythrin (PE) conjugated fluorescent anti-human IgG, IgG1, IgG2, IgG3, or IgG4 antibody (1.3 µg/mL in 1% BSA/PBS, Southern Biotech, Birmingham, AL, USA). To detect IgM, beads were incubated with a primary biotinylated anti-human IgM antibody (1.3 µg/mL in 1% BSA/PBS, MabTech, Sweden), for 2 h, followed by streptavidin-PE conjugated secondary detector (1.3 µg/mL in 1% BSA/PBS, Thermo Fisher Scientific, Waltham, MA, USA), for 1 h. To detect Fc receptor binding to antibodies, biotinylated recombinant human FcγRIIb and FcγRIIIb monomers (ACRObiosystems, Newark, DE, USA) were pre-conjugated to streptavidin-PE at a ratio of 4:1 mol to form fluorescent tetramers. FcγRIIa-His^131^ and FcγRIIIa-Val^158^ were available as biotinylated, soluble homodimers [27]. Tetramers or dimers were diluted to 1.3 µg/mL in 1% BSA/PBS and incubated with beads, for 2 h. To detect C1q binding to antibody, recombinant C1q (MP Biochemicals, Irvine, CA, USA) was biotinylated using the EZ-Link™Sulfo-NHS-LC-Biotin (Thermo Fisher Scientific, Waltham, MA, USA) according to the manufacturer’s instructions, using a 1:5 mol ratio of C1q to biotin. Biotinylated recombinant C1q was conjugated to streptavidin-PE at a 4:1 mol ratio to form fluorescent tetramers, which were diluted to 15.92 µg/mL in 1% BSA/PBS and incubated with beads, for 2 h. Median fluorescent intensities (MFI) from a minimum of 40 beads per region were acquired on a Luminex instrument (Bio-Plex® MAGPIX™ or Flexmap3D). Experiments were performed once with 37% of samples duplicated in each plate.

### Infected erythrocyte selection

*P. falciparum* IE were cultured in type O + human erythrocytes from healthy donors, as previously described [28]. 3D7 and IT4 IE were selected for expression of PfEMP1 that are predicted to bind to ICAM-1 (3D7VAR04 and IT4VAR13), as described in Joergensen et al. [29], using antibody raised against the ICAM-1 binding DBLβ domain of 3D7VAR04 (Pfd1235w) [30] and IT4VAR13 PfEMP1 [6]. 3D7VAR04 were further enriched by fluorescence activated cell sorting. Isolated trophozoite stage IE were incubated with 0.16 mg/mL monoclonal mouse anti-human VAR04 DBLβ3 [30] and 4 μg/mL anti-IgG Fc-AF647 (Invitrogen) secondary antibody. IE were gated by forward and side scatter using unstained IE and AF647 labeled IE were sorted. The percentage of IE in culture expressing the selected PfEMP1 was monitored by flow cytometry (CytoFLEX, Beckman Coulter). IE were stained with 25 µg/mL dihydroethidium bromide (DHE, Sigma) and 0.16 mg/mL mouse anti-human VAR04 DBLβ or rat anti-serum against VAR13 DBLβ3 [6], and 4 μg/mL anti-IgG Fc-AF647 secondary antibody. Gating for AF647 positive events was set using stain-free controls. The estimated percentage of parasites expressing VAR04 (Pfd1235w) prior to experiments ranged from 44 to 55% and the estimated expression of VAR13 ranged from 36 to 52%.

### Antibody-dependent neutrophil and THP-1 cell phagocytosis assay

Antibody-dependent THP-1 cell phagocytosis of 3D7VAR04 and IT4VAR13 IE was performed as described in Ataide et al. [31] and in Kassa et al. [32]. The THP-1 monocyte-like cell line, which expresses FcγRI and FcγRIIa/b but lacks FcγRIIIa/b, has been used to study antibody-dependent cellular phagocytosis for some time [33]. Antibody-dependent neutrophil phagocytosis of 3D7VAR04 and IT4VAR13 IE was performed as described in Aitken et al. [19]. Neutrophils were isolated from fresh venous blood from two healthy non-immune volunteers using the EasySep Direct Human Neutrophil Isolation Kit (STEMCELL Technologies, Tullamarine, Australia) as described [19]. IE were labeled with dihydroethidium bromide and the “percentage phagocytosis” was defined as the frequency of THP-1 or neutrophil cell events that were positive for DHE, relative to a rabbit anti-human red blood cell positive control serum. For neutrophils, the average percentage phagocytosis from the two experiments was used. Wilcoxon rank sum test was used to compare median antibody responses between groups. Each experiment was performed once with all samples in duplicate.

### Statistical analysis for identification of key antibody features

Prior to analysis, we subtracted the serum-free control and antigen-free control region MFI. We normalized between plates by fitting a linear or non-linear equation to 36 duplicated samples from each plate. Negative fluorescence intensities were converted to a value of 1. Antigen specific antibody features were labeled with the following format: “Fc feature.protein identifier_domain description” (see Additional file 1: Table 1). Four variables were eliminated as all values were below background (FcgRIIIb.SM14_DBLγ3, C1q.SM5_DBLβ3, FcgRIIb.SM26_CIDRγ12, IgG2.SM5_DBLβ3) and two antigens were not included for all Fc detectors due to low protein availability (SM27_DBLδ7 and UM1_DBLα0.13, only probed for IgG, IgG1, IgG2, IgG3, and IgG4). Merozoite and sporozoite antigens were not included for all Fc detectors due to low protein availability (AMA1 only probed for IgG and IgG1, MSP3 not probed for IgG1, EBA175-RIII-B not probed for IgG1, IgG2, IgG3, and IgG4, and CSP not probed for Fcγ receptors).

To compare the mean difference in antibody levels for each antigen between patients with cerebral malaria and uncomplicated malaria, data were log(*x* + 1) transformed (to adjust for right skewedness) and the means were compared by a Welch’s *t*-test. A *p* value < 0.05 and a log_2_(fold change) > 1 (i.e., > twofold change in geometric means of antibody levels) were considered significant differences and there was no adjustment for multiple comparisons. To evaluate the influence of age, we divided the population around the median age of 49 months for some analyses. To group responses to multiple proteins, the geometric mean of responses was calculated for each individual. To select a combination of features that best distinguish between cerebral and uncomplicated malaria, we performed multivariable logistic regression coupled with machine learning, as previously described with minor changes [19]. Merozoite antigens were excluded from the multivariable analysis. Data were log(*x* + 1) transformed, mean centered, and scaled to one standard deviation. Missing values were imputed as the median of five imputed data sets using multivariate imputations by chained equations [34] with predictive mean matching. Elastic net regularized logistic regression (ENLR) [35] was used to identify features that best distinguish between cerebral and uncomplicated malaria. We performed 5000 repeats of ENLR with the *α* tuning parameter set to 0.5. For each repeat, the data were randomly split into 10 folds, with nine folds used to fit the model across a range of lambda values and one fold to assess the area under the receiver operator curve (AUROC, tenfold cross validation). The features that appeared in the model with the greatest AUROC and the odds ratio (OR) for each feature were recorded. Features were ranked based on the frequency with which they appeared in the model with the greatest AUROC across 50,000 models (5000 repeats with tenfold cross validation). Alpha tuning parameters from 0.25 or 1 were also assessed. To determine a minimum set of variables that could be linearly combined by partial least squares regression (PLSR) to explain the variation in cerebral and uncomplicated malaria, we added one feature at a time to a PLSR model, in order of frequency that they appeared in the ENLR. We performed 500 repeats of tenfold cross validation to estimate the AUROC after the addition of each feature and selected the top features whose addition resulted in a significant increase in the AUROC. We assessed the performance of the linear combination of selected features to classify samples as cerebral and uncomplicated malaria by fitting the selected features to a PLSR model and computing the AUROC (as above, the estimation was from 500 repeats of tenfold cross validation). The PLSR performance was also assessed with randomly shuffled outcome variables and random feature selection.

## Results

### Characteristics of the Malawian children

The final cohort included 51 children with cerebral malaria (median age of 51 months) and 46 with uncomplicated malaria (median age of 48 months). Children with cerebral malaria had a Blantyre coma score of 0–2 and 42 (82%) were retinopathy positive [36] (Table 1).

**Table 1:** Summary of study population categorized by disease severity

Characteristic	Uncomplicated malaria (*n* = 46)	Cerebral malaria (*n* = 51)
**Age, median [IQR], months**	48 [29–88]	51 [28–83]
**Age group, *n* (%)**		
0–48 months	24 (52%)	25 (49%)
49 + months	22 (48%)	26 (51%)
**Sex, *n* (%)**		
Female	19 (41%)	16 (31%)
Male	27 (59%)	35 (69%)
**Location, *n* (%)**		
Urban	25 (54%)	25 (49%)
Rural	21 (46%)	26 (51%)
**BCS, *n* (%)**		
0	0 (0%)	5 (10%)
1	0 (0%)	15 (29%)
2	0 (0%)	31 (61%)
5	46 (100%)	0 (0%)
**Retinopathy positive, *n* (%)**	0	42 (82%)
**Severe anemia, *n* (%)**	0	11 (23%)
**Temperature, median [IQR], degrees Celsius**	38.6 [37.8–39.3]	39 [38.5–39.4]
**Parasitemia, median [IQR], per mL**	N/A	4599 [396–58,880]
**Hemoglobin, median [IQR], g/dL**	10.4 [9.0–11.7]	8.2 [7.2–9.2]

Slides from children with uncomplicated malaria were counted using ‘+’s. Actual parasitaemias were not available

*IQR* Inter-quartile range, *N/A* Data not available

### Univariate analysis of differences in antibody responses in cerebral and uncomplicated malaria

We measured antibody responses to 39 recombinant PfEMP1 domains that have been associated with severe malaria, cerebral malaria, or uncomplicated malaria. We also included 5 recombinant merozoite and sporozoite antigens, an antigen-free negative control, and tetanus toxoid as a positive control. We measured 11 antigen-specific antibody Fc features: IgG, IgM, IgG subclasses IgG1, IgG2, IgG3, and IgG4, and antibody Fc binding to recombinant FcγRIIa, FcγRIIb, FcγRIIIa, FcγRIIIb, and C1q. The final analysis included a total of 451 features, 413 of which were related to PfEMP1 antigens and 38 of which were related to merozoite or sporozoite antigens.

**Fig. 1:**
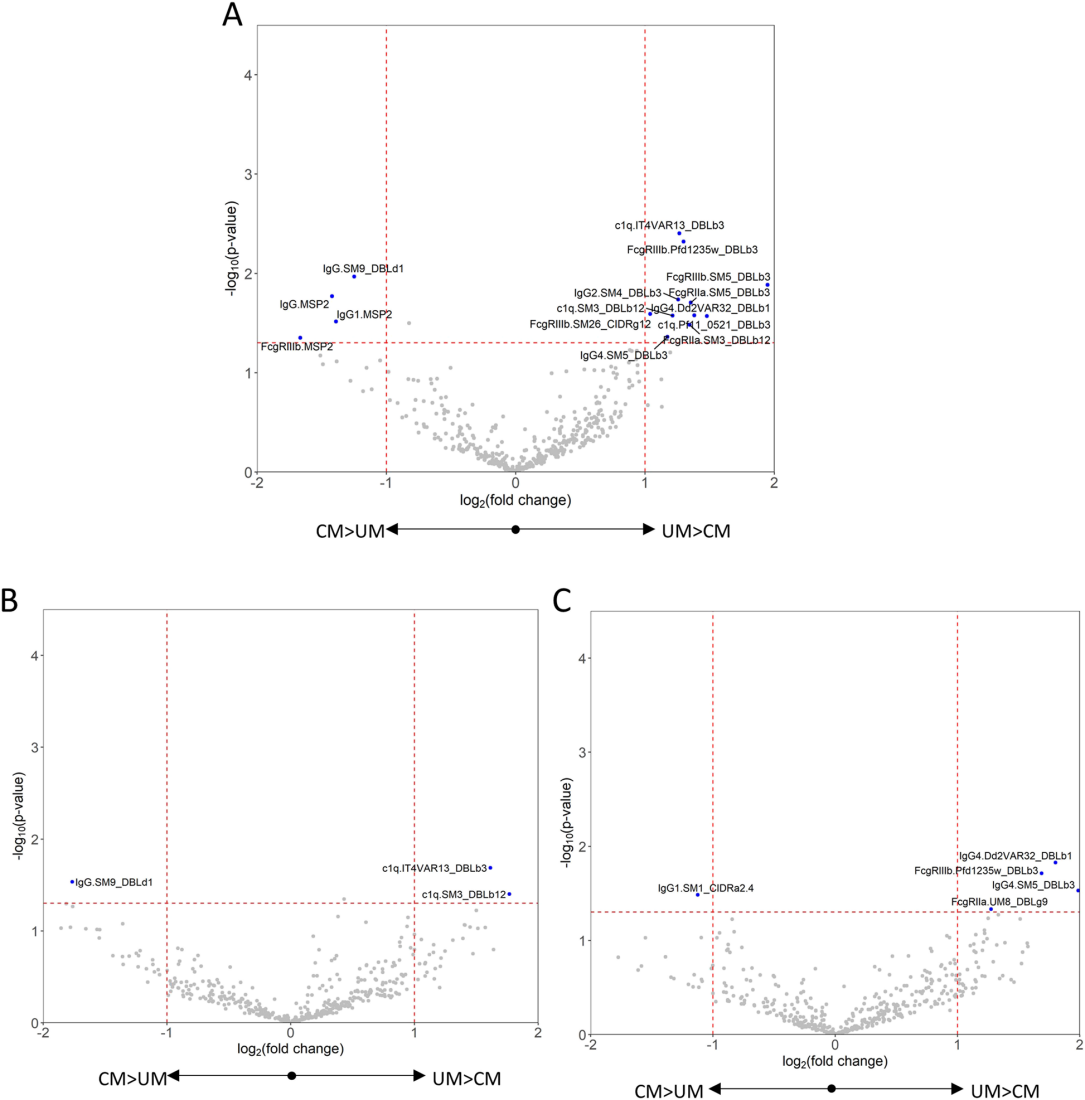
Individual antibody features to recombinant proteins compared between cerebral and uncomplicated malaria. **A** All individuals, **B** children under 49 months, **C** children 49 months and older. X-axis represents the magnitude of difference (log2 transformed) between the geometric mean antibody levels of the cerebral and uncomplicated malaria groups. Vertical lines at log_2_(2) and log_2_(0.50) indicate a twofold elevation in uncomplicated malaria or cerebral malaria, respectively. Y-axis represents − log_10_ transformed *p* value from Welch’s *t*-test comparison of cerebral and uncomplicated malaria. Horizontal line indicates log_10_(0.05) threshold of statistical significance, and there were no adjustments for multiple comparisons. DBL Duffy binding like domain, CIDR cysteine-rich interdomain region, a α, b β, d δ, e ε, g γ, z ζ

Half of the recombinant PfEMP1 proteins (19 out of 39) were recognized by IgG antibodies in at least 25% of children with cerebral and uncomplicated malaria (see Additional file 1: Fig. S3). We first used a volcano plot to display univariate analyses comparing antibody responses for each PfEMP1 protein and antibody Fc features between children with cerebral and uncomplicated malaria. IgG to one PfEMP1 domain, SM9_DBLδ1, was significantly elevated by greater than twofold in cerebral malaria compared to uncomplicated malaria (Fig. 1A). IgG, IgG1, and FcγRIIIb responses to the merozoite antigen, MSP2, were significantly higher in cerebral than uncomplicated malaria and there were no other significant differences for other non-PfEMP1 antigens. Antibody features that were significantly elevated in uncomplicated malaria were IgG2 targeting SM4_DBLβ, IgG4 targeting SM5_DBLβ, IgG4 targeting Dd2VAR32_DBLβ1, FcγRIIa binding antibodies targeting SM5_DBLβ and SM3_DBLβ12, FcγRIIIb binding antibodies targeting PFD1235w_DBLβ, SM26_CIDRγ12, and SM5_DBLβ, and C1q fixing antibodies targeting IT4VAR13_DBLβ and PF11_0521_DBLβ (Fig. 1A).

Given that age is an important determinant of PfEMP1 antibody [37], we divided the children into those older or younger than the median age of 49 months. Amongst children under 49 months old, IgG targeting SM9_DBLδ1 remained significantly greater in cerebral malaria, and C1q response to IT4VAR13_DBLβ3 remained significantly greater in uncomplicated malaria. C1q response to SM3_DBLβ12 was also significantly greater in uncomplicated malaria (Fig. 1B). In children over 49 months, IgG1 to SM1_CIDRα2.4 was significantly greater in cerebral malaria compared to uncomplicated malaria, although it was not significantly elevated in all children combined. Features that were significantly higher in uncomplicated malaria were IgG4 to Dd2VAR32_DBLβ1 and SM5_DBLβ3, FcγRIIIb binding antibodies targeting PFD1235w_DBLβ, and FcγRIIa binding antibodies targeting UM8_DBLγ9 (Fig. 1C). The last of these was not significantly increased in the whole group in Fig. 1A. Amongst children with cerebral malaria or uncomplicated malaria, there were no correlations between age and antibody responses to features that were significantly different in cerebral and uncomplicated malaria (see Additional file 1: Figs. S4 and S5).

Due to the potential importance of DBLβ domains in cerebral malaria, we grouped each individual’s responses to DBLβ domains based on binding phenotype (using the geometric mean): group A DBLβ that bind ICAM-1 with the DBLβ_motif_ ICAM-1-binding motif, group A DBLβ that do not bind ICAM-1, and group B DBLβ that bind ICAM-1, but do not contain DBL_motif_ (Additional file 1, Additional file 2). IgG1 antibodies targeting group B DBLβ were higher in children with cerebral malaria (Welch’s *t*-test *p* value 0.032). Antibodies that engage c1q and target ICAM-1-binding group A DBLβ_motif_ and group B DBLβ domains were higher in children with uncomplicated malaria compared to cerebral malaria (Welch’s *t*-test *p* value 0.032 and 0.024, respectively) whereas antibodies targeting group A non-ICAM-1 binding DBLβ domains did not differ significantly between the two clinical groups. We also grouped responses to domains that were upregulated in severe or uncomplicated malaria in a previous study in Indonesian adults and children [10], labeled as “SM” or “UM” (Additional file 1, Additional file 2). Here, IgG1 antibodies that target UM proteins were higher in cerebral malaria compared to uncomplicated malaria (*p* value 0.004). There were no significant differences between cerebral malaria and uncomplicated malaria in grouped responses to domains associated with DC8. IgG1 antibodies to group B proteins and to all proteins grouped were higher in cerebral malaria compared to uncomplicated malaria (*p* value 0.0092 and 0.017, respectively). All individual comparisons between cerebral and uncomplicated malaria for each protein and Fc feature are included in Additional file 3.

### Multivariate analysis to select the combination of features that best discriminates between cerebral malaria and uncomplicated malaria

**Fig. 2:**
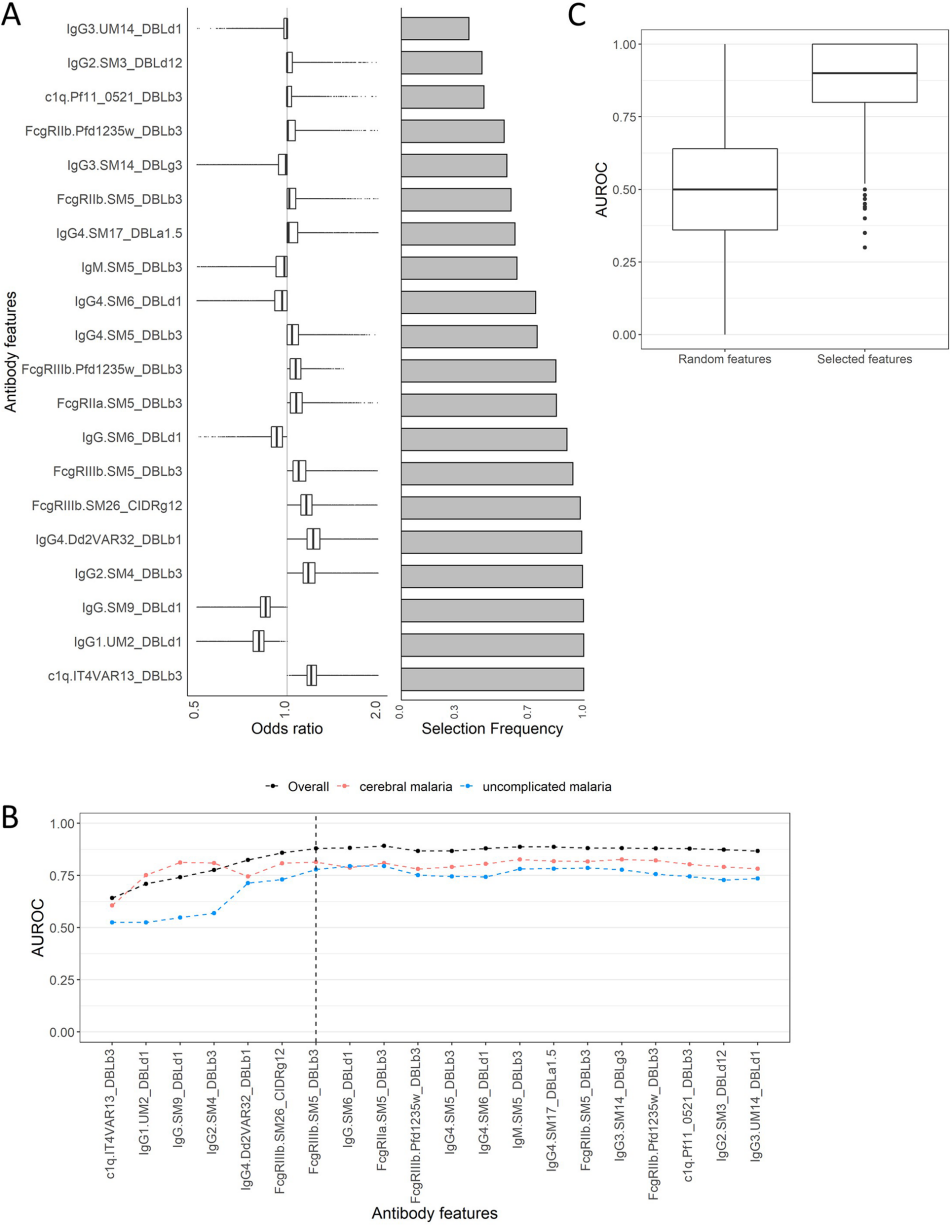
Multivariate analysis to select a minimum combination of features that best distinguishes between cerebral and uncomplicated malaria. A combination of elastic net regularized logistic regression (ENLR) and partial least squares (PLS) regression was used. **A** Odds ratio of antibody features from 5000 repeats of tenfold cross validated ENLR models, in order of selection frequency (top 20 most selected variables are shown). Features with median odds ratio greater than 1 represents responses associated with increased odds of uncomplicated malaria and features with median odds ratio less than 1 are associated with increased odds of cerebral malaria. **B** Performance of PLS model after addition of features (x-axis) in order of ENLR selection frequency (left to right), as measured by AUROC from 5000 repeats of tenfold cross validated PLS regression. Black line shows AUROC for all children, red line shows the accuracy of classifying children with cerebral malaria only, and blue line the accuracy of classifying children with uncomplicated malaria only. Vertical dashed line represents point at which the addition of one or two more features does not significantly increase the AUROC which occurs at seven features (referred to as the “selected features”). **C** Performance of PLS regression models using only the seven selected features from ENLR (87% median, 80–96% IQR), compared to randomly selected combinations of seven features (50% median, 40–65% IQR). AUROC corresponds to 5000 repeats of tenfold cross validated PLS regression models. AUROC area under the receiver operating characteristic curve. Box plots show median and interquartile range (IQR) and whiskers show points within Q1 − 1.5*IQR and Q3 + 1.5*IQR. DBL Duffy binding like domain, CIDR cysteine-rich interdomain region, a α, b β, d δ, e ε, g γ, z ζ

**Fig. 3:**
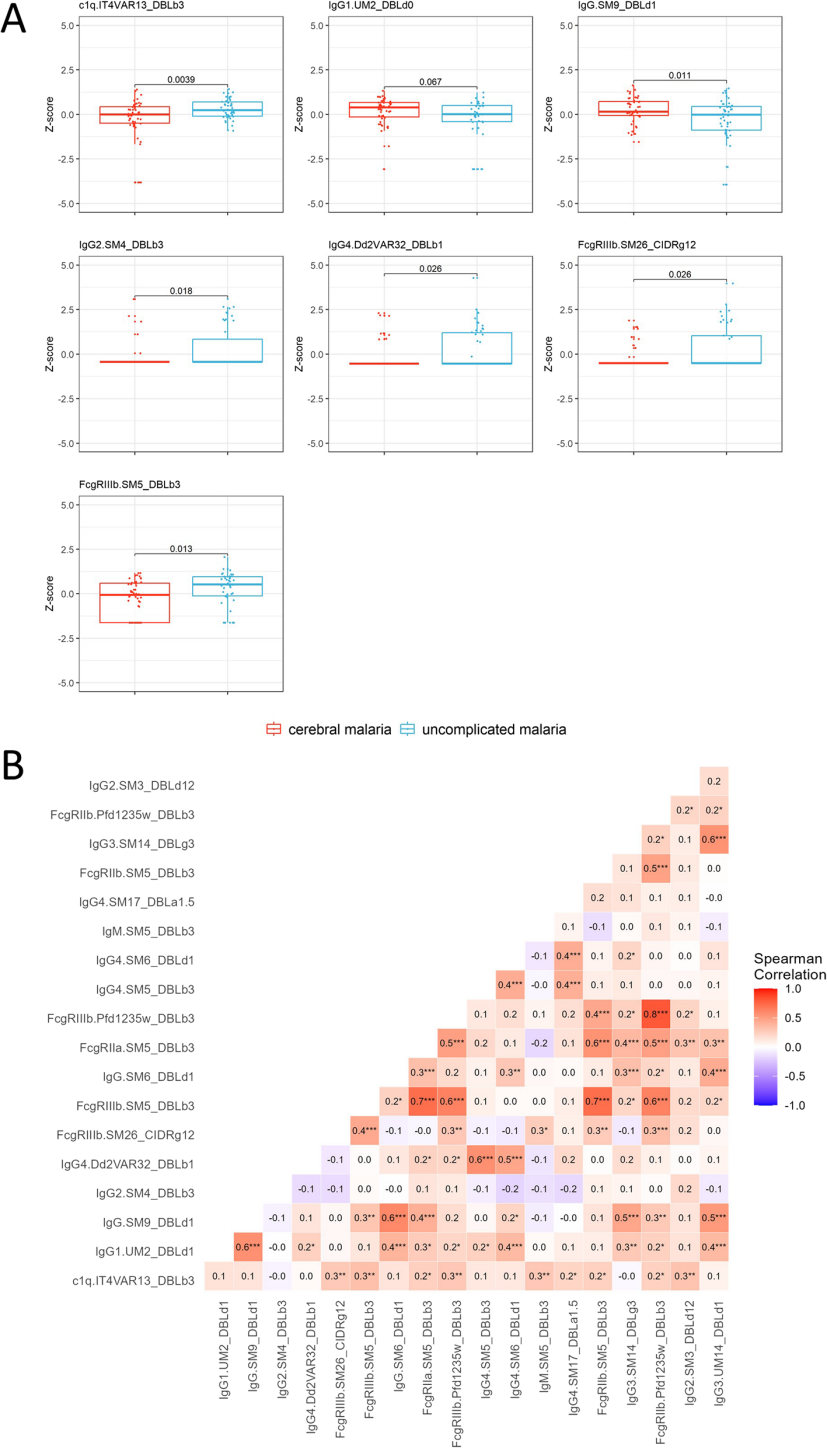
Antibody features associated with protection from cerebral malaria and their correlation with one another. **A** Distribution of seven selected features in children with cerebral malaria (red) and uncomplicated malaria (blue). MFI readouts were log(*x* + 1) transformed, mean centered, and scaled to 1 standard deviation (*z*-score). Box plots show median and interquartile range (IQR) and whiskers show IQR + 1.5*IQR. Horizontal bars represent Welch’s *t*-test comparison with *p* value shown. **B** Spearman correlation of features that appeared in > 70% of ENLR model iterations, including the seven selected features, using non-transformed MFI values. DBL Duffy binding like domain, CIDR cysteine-rich interdomain region, a α, b β, d δ, e ε, g γ, z ζ. *p value < 0.05, **p value < 0.01, ***p value < 0.001

To identify the antibody features that best differentiate between cerebral and uncomplicated malaria, we performed repeated elastic net regularized logistic regression (ENLR) [35] with tenfold cross validation and recorded the frequency and odds ratio (OR) of features selected in each model iteration, as described in Gunn et al. and Aitken et al. [19, 38]. Area under the ROC (AUROC) was used as the metric of discrimination power. The 20 most frequently selected features included both features associated with an increased odds of cerebral malaria and of uncomplicated malaria (Fig. 2A). To select a minimum combination of features that best discriminate between cerebral and uncomplicated malaria, we added features one at a time to a PLS regression model, in order of the frequency of selection based on the effect size of the odds ratio from the ENLR (Fig. 2A) and assessed the AUROC (Fig. 2B). After the seventh variable was included in the model, the AUROC did not increase by adding further variables. The top seven most frequently selected features were C1q fixing antibodies targeting IT4VAR13_DBLβ3, IgG1 targeting UM2_DBLδ1, IgG targeting SM9_DBLδ1, IgG2 targeting SM4_DBLβ3, IgG4 targeting Dd2VAR32 DBLβ1, and antibodies targeting SM26_CIDRγ12 or SM5_DBLβ3 that engage FcγRIIIb (Fig. 2B). The univariate analyses for these features are shown in Fig. 3A. Altering the *α* tuning parameter to 0.25 or 1 did not change the features that were most frequently selected (see Additional file 1: Figs. S6 and S7).

To assess the predictive power of our seven selected features, we compared their ability to correctly classify children to that of seven randomly selected features. Based on the AUROC, the seven selected features could predict clinical presentation with 87% accuracy (median, 80–96% IQR) whereas seven randomly selected features could predict clinical presentation with 50% accuracy (40–53% IQR), equivalent to chance alone (Fig. 2C). The seven selected features did not generally correlate strongly with each other or with the 20 features that frequently appeared in the ENLR model iterations (Fig. 3B). There were moderate correlations between DBLδ1 domains (IgG.UM2_DBLδ1, IgG_SM9_DBLδ1, IgG.SM6_DBLδ1; Spearman correlation coefficient *R* = 0.39–0.60), moderate to strong correlations between FcγR binding to antibodies targeting group A DBLβ domains (FcγRIIa.SM5_DBLβ3, FcγRIIb.SM5_DBLβ3, FcγRIIIb.SM5_DBLβ3, FcγRIIIb.PFD1235w_DBLβ3, FcγRIIb.PFD1235w_DBLβ3, *R* = 0.45–0.81), and a moderate correlation between IgG4 responses to group A DBLβ domains (IgG4.Dd2VAR32_DBLβ1 with IgG4.SM5_DBLβ3, *R* = 0.57). We performed similar correlation analyses for children with severe malaria only or uncomplicated malaria only (Additional file 1: Figs. S8 and S9).

### Antibody-dependent neutrophil phagocytosis of ICAM-1 + EPCR binding IE is associated with protection from cerebral malaria

**Fig. 4:**
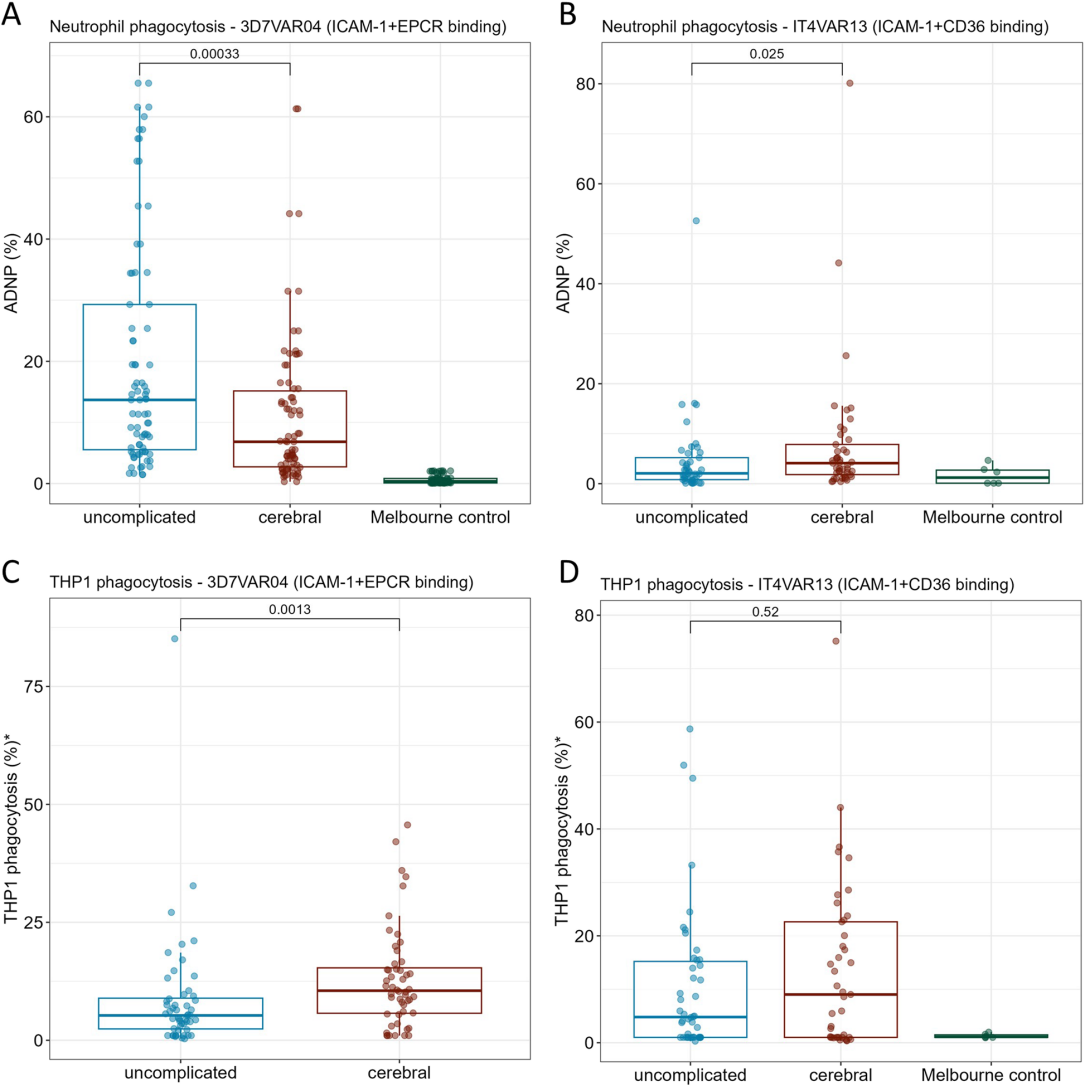
Antibody-dependent neutrophil phagocytosis and THP-1 cell phagocytosis of ICAM-1 binding IE. **A** Neutrophil phagocytosis of 3D7VAR04 ICAM-1 + EPCR co-binding IE and **B** IT4VAR13 ICAM-1 + CD36 co-binding IE. Mean responses from two neutrophil donors shown. **C** THP-1 cell phagocytosis of 3D7VAR04 ICAM-1 + EPCR co-binding IE and **D** IT4VAR13 ICAM-1 + CD36 co-binding IE. IE were opsonized with plasma from Malawian children with cerebral or uncomplicated malaria, or healthy Melbourne donors (Melbourne control). Y-axis (% phagocytosis) represents percentage of neutrophils or THP-1 cells associated with DHE stained IE, relative to a positive control serum. Boxes represent median and interquartile range (IQR) from Q1 to Q3, and whiskers range from (Q1 − 1.5*IQR) to (Q3 + 1.5*IQR). Medians (horizontal bars) were compared by Wilcoxon rank sum test and associated *p* values are shown

To assess the role of FcγRIIIb binding to antibodies targeting ICAM-1 binding DBLβ domains in protection from cerebral malaria, we measured antibody-dependent neutrophil phagocytosis (ADNP) of IE selected for expression of 3D7VAR04 (Pfd1235w), which co-binds to ICAM-1 and EPCR, and IT4VAR13, which co-binds to ICAM-1 and CD36 (Fig. 4). Children with uncomplicated malaria had higher ADNP of ICAM-1 + EPCR binding IE compared to children with cerebral malaria (median (IQR), 15% (8–34%) and 7% (3–15%), respectively, Wilcoxon signed-rank test *p* < 0.001). Children with cerebral malaria had higher ADNP of ICAM-1 + CD36 binding IE than children with uncomplicated malaria (4% (2–7%) and 2% (1–3%), respectively, *p* = 0.025). We also measured antibody-dependent cellular phagocytosis (ADCP) of ICAM-1 binding IE by THP-1 cells, which lack FcγRIIIb. Children with cerebral malaria had higher ADCP of ICAM-1 + EPCR binding IE compared to children with uncomplicated malaria (10% (6–15%) and 6% (2–9%), respectively, *p* = 0.001) and there was no statistically significant difference in THP-1 cell phagocytosis of ICAM-1 + CD36 binding IE between children with cerebral and uncomplicated malaria (9% (1–22%) and 5% (1–15%), respectively, *p* = 0.52). ADNP and ADCP using IE expressing 3D7VAR04 and ITVAR13 did not correlate with the features of the antibody response to the DBLβ3 domain of Pfd1235w or the DBLβ3 domain of VAR13, respectively (Figs. S10 and S11).

## Discussion

PfEMP1 plays a key role in the pathogenesis of cerebral malaria; however, previous studies have been unable to identify a single PfEMP1 antigen to which IgG antibodies are consistently associated with protection. In this study, we performed a detailed characterization of the targets and Fc features of antibodies to PfEMP1 antigens in Malawian children with cerebral malaria and uncomplicated malaria. In univariate analyses, responses that were better developed in uncomplicated malaria included antibodies engaging FcγRIIa, FcγRIIIb and C1q, and IgG4 and IgG2 responses, primarily targeting DBLβ domains. Only responses to MSP2 and IgG to one DBLδ domain were higher in cerebral than uncomplicated malaria.

We then used a previously published statistical approach that combines logistic regression and machine learning [19, 38] as an unbiased method to select a combination of features that could best differentiate antibody responses of children with cerebral and uncomplicated malaria. We found few differences in IgG but identified a combination of seven antigen-specific antibody Fc features that could differentiate between the groups with 87% accuracy, whereas randomly selected antibody features could not predict clinical presentation. This supports the idea that specific PfEMP1 variants are involved in the pathogenesis of cerebral malaria and that specific features of the Fc domains of antibodies to these variants facilitate IE clearance and prevent the development of cerebral malaria.

Four of the seven selected antibody features were targeting DBLβ domains and were associated with decreased odds of cerebral malaria. Upregulated expression of group A DBLβ_motif_ that contain the ICAM-1-binding motif, predictive of dual ICAM-1 and EPCR receptor binding IEs, has been clearly linked to cerebral malaria [5–8], but the role of antibodies targeting these domains in protection from cerebral malaria is less clear. Antibodies targeting group A ICAM-1 binding DBLβ domains can block cytoadhesion to human brain endothelial cells and have been associated with protection from cerebral malaria in some studies [21, 39] but not in others [40, 41]. An advantage of this study is that we were able to probe multiple examples of DBLβ domains, as well as multiple antibody Fc features to identify correlates of protection. The selected combination of features included a group A ICAM-1 binding domain with the DBLβ_motif_, as well as two group A non-ICAM-1 binding domains that lack the motif and a group B domain that binds to ICAM-1 but lacks the motif. These responses are likely influenced by antibody cross reactivity between DBLβ domains [21] and may be influenced by variants causing the current infection, as described in Olsen et al. [24]*.*

Other PfEMP1 targets amongst the selected combination of features included two DBLδ1 domains and a CIDRγ12 domain. DBLδ1 is one of the most diverse domains [42] and there is limited data on its functions, which may be equally diverse [43, 44]. In contrast to our study, antibodies to group B and C DBLδ1 have previously been associated with protection from severe malaria (for UM2_DBLδ1) [13] and reduced prospective risk of hyperparasitemia [25]. In our study, antibody responses to the DBLδ1 domains (UM2 and SM9) were highly correlated and therefore it is difficult to discern whether the antibody response in children with cerebral malaria is driven by proteins more commonly expressed in severe malaria (SM9_DBLδ1) or uncomplicated malaria (UM2_DBLδ1). Similarly, little is known about the function of SM26_CIDRγ12, other than that CIDRγ are part of the head structure of a rosetting parasite line [45]. Antibodies to SM26_CIDRγ12 were elevated in uncomplicated malaria compared to severe malaria in Indonesian adults and children [10] and may be a novel target of protective antibodies to cerebral malaria.

The selected combination of seven antibody features that could best distinguish between cerebral and uncomplicated malaria included antigen specific antibody engagement with C1q and FcγRIIIb responses and IgG2 and IgG4 responses. Recent work from our group has shown that complement deposition leads to antibody mediated lysis of VAR2CSA IE at high concentrations of antibodies (unpublished). However, another recent study found that complement component 1 s (C1s) cleaves PfEMP1 at sites found in interdomain regions, including in IT4VAR13 [46]. Additionally, C1q deposition relies on formation of antibody hexamers and the distribution of PfEMP1 and antibodies on the bead-based multiplex platform are unlikely to accurately represent the surface of the IE [47]. Antibodies to malaria sporozoites that fix complement can inhibit their motility and invasion [48], and complement fixation can enhance antibody mediated phagocytosis [49]. Therefore, the relevance of C1q deposition at the DBLβ3 domain to downstream effects on the IE requires further study. FcγRIIIb is highly expressed on neutrophils and can work in concert with FcγRIIa to induce ADNP [50] but is not involved in the release of reactive oxygen species [51].

The detection of IgG2 and IgG4 specific antibodies as correlates of protection was unexpected, but one or both antibody subclass responses have been identified as correlates of protection in malaria vaccine studies [52, 53] and IgG4 to CSP was a correlate of naturally acquired protection in Malian children [54]. Antibody responses to recombinant group A and group B ICAM-1 binders are dominated by the cytophilic subclasses, IgG1 and IgG3 [55], and most children we studied had low or undetectable IgG2 and IgG4. Their selection may reflect greater class switch recombination associated with a more mature antibody response [56] as indicated by their selection in older rather than younger children. Although the specific function of IgG2 and IgG4 targeting PfEMP1 is unclear, they may be useful biomarkers of protective immunity.

ADNP is emerging as a correlate of naturally acquired protection from malaria in pregnancy and children [19, 54] and as an important correlate of vaccine-induced immunity [57–59]. ADNP has been implicated in clearance of VAR2CSA expressing IE [19] and ICAM-1 binding IE [16]. To further assess the role of FcγRIIIb binding to antibodies targeting ICAM-1 binding DBLβ domains in protection from cerebral malaria, we measured ADNP of two lines of ICAM-1 binding IE. In line with the recombinant protein interactions, we found that ADNP of IE expressing ICAM-1 + EPCR binding IE (that contain DBLβ3_Pfd1235w) was elevated in uncomplicated malaria compared to cerebral malaria, in keeping with the published associations between ICAM-1 + EPCR binding PfEMP1 types and cerebral malaria [6]. In contrast, ADNP of ICAM-1 + CD36 binding IE (that contain DBLβ3_IT4VAR13) was elevated in cerebral malaria. Further, phagocytosis of ICAM-1 + EPCR binding IE by THP-1 cells, which lack FcγRIIIa or b, was elevated in cerebral malaria. This suggests that children who are protected from cerebral malaria have FcγRIIIb binding antibodies that target ICAM-1 binding DBLβ and which promote phagocytosis of ICAM-1 + EPCR binding IE by neutrophils. The higher levels of phagocytosis of these ICAM-1 + EPCR binding IE by THP-1 cells in the CM group were unexpected and warrant further investigation, and future studies will further characterize the relationship between antibodies to recombinant domains and to IE expressing the intact PfEMP1.

Systems serology is based on the idea that “neutralizing” antibody titers alone do not capture the complexity of the immune response. In the context of malaria, systems serology has been applied to characterize functional antibody responses to Rh5 vaccination [60], RTS,S/AS01 vaccination [52], placental malaria [19], and protection from parasitemia and clinical malaria in children [54]. Like those studies, we observed that total IgG may not be an accurate predictor of protection from malaria. This finding has been mirrored in a recent study of immunity to IE in pregnant women, in which functional antibodies that promoted phagocytosis by monocytes or neutrophils were better at differentiating between women who were protected from placental malaria or who were susceptible, compared to total IgG levels towards recombinant VAR2CSA proteins or the VAR2CSA-expressing placental binding IE [19]. The present study, the placental malaria study [19], and the study of protection from clinical malaria [54] all indicate that a broad range of antibody responses contribute to protection from malaria, findings that have implications for the development of new tools to prevent severe malaria syndromes such as cerebral malaria. Similarly, IgG titer against DBLβ domains did not differ in Beninese children with severe or uncomplicated malaria, but opsonic phagocytosis by THP-1 cells induced by a DBLβ3 (group B, IT4VAR13) was elevated in uncomplicated malaria [55]. Our study supports these findings to suggest that rather than the quantity of antibodies targeting PfEMP1, the Fc features and downstream functional activity of antibodies may be more useful correlates of protection.

The interactions between antibodies, recombinant parasite proteins, and recombinant Fc receptors measured by multiplex immunoassay did not correlate with phagocytosis assays using IE (see Additional file 1: Figs. S10 and S11). However, based on the antibody features identified using systems serology, we were able to hypothesize and confirm that ADNP of ICAM-1 + EPCR binding IE is associated with protection from cerebral malaria. Therefore, systems serology using recombinant proteins is an effective tool to identify potential mechanisms of protective immunity against IE, but further studies are required to better understand the differences between antibody that binds to a single recombinant protein domain and is detected using isolated Fc receptors, and antibody to a whole PfEMP1 in its native form, measured using a live phagocytic cell.

The pathogenesis of cerebral malaria likely involves multiple factors, including PfEMP1 mediated sequestration of IE in the microvasculature, activation of coagulation pathways, endothelial cell activation, and breakdown of the blood–brain barrier [61]. Previous studies have shown that antibodies targeting DBLβ domains can block cytoadhesion of IE and may function to reduce IE sequestration in the brain [21]. Our study adds to current knowledge of protective immunity to cerebral malaria by suggesting that antibodies may facilitate clearance of IE expressing variants of PfEMP1 that are associated with severe malaria, including PfEMP1 with ICAM-1 binding DBLβ domains and CIDRy12 domains, via FcγRIIIb and C1q mediated pathways such as phagocytosis.

Strengths of the study include well-characterized children with cerebral or uncomplicated malaria who were matched for location, and assessment of a broad array of responses to PfEMP1 antigens, including protein products of genes known to be expressed in severe or uncomplicated malaria, and PfEMP1 types known to bind to key endothelial receptors. As we have captured a single time point in single infections, interpretation requires caution, and convalescent samples would provide useful insight into the evolution of antibody responses following infection. The dynamics of the antibody response do not necessarily align with those of the disease and the observed responses are likely to be influenced by the timing of sampling. We expect there is variation in the duration of infection prior to presentation to hospital, which we partially accounted for by matching for residency location. However, we were unable to account for the possibility that children with uncomplicated malaria may have progressed to severe malaria without timely treatment. Higher IgG to MSP2 in cerebral malaria compared to uncomplicated malaria may suggest differences in exposure, despite matching of residency location, although there were no differences in IgG responses to other non-PfEMP1 antigens. There is a possibility of differences in exposure over the 3-year sample collection period. Additionally, the antibody responses associated with protection from a single malaria episode do not necessarily represent complete or ongoing protection. Further limitations of this study include the use of recombinant proteins that may not reflect the native protein structure or capture epitopes that span multiple domains, and a relatively small sample size. Stratifying our data by participant age may have limited our power to find PfEMP1 antibody responses that are age dependent. Protein expression system did not appear to bias the likelihood of selection. Domains expressed in both WGCF and *Escherichia coli* systems appeared in the top features, including examples of DBLβ domains expressed in both systems. Twenty-eight of the 39 PfEMP1 antigens probed in this study were identified in Papuan adults and children with severe malaria, and it is possible that these domains may be more relevant to manifestations of severe malaria other than cerebral malaria or may be more relevant in Papua than Africa, and even better separation of groups might be obtained with more locally adapted multiplex protein arrays. Due to limited plasma availability, experiments were conducted once. Validation in other sample sets will be important. Expression of PfEMP1 with EPCR binding CIDRα1 domains has been associated with severe malaria in several studies; however, only one CIDRα1 domain (CIDRα1.6) was well recognized in this population and other variants may be more relevant. It is also possible that including multiple examples of some domains may have increased the probability of them appearing in the selected features.

In this exploratory study, we performed a detailed characterization of the domain specific antibody responses in children with cerebral malaria. The systems serology approach may be used in future studies to characterize geographical and age-dependent differences in the antibody response to PfEMP1 variants, as well as temporal associations with protection. Half of PfEMP1 antigens were recognized by less than 25% of children with cerebral or uncomplicated malaria (see Additional file 1: Fig. S3), indicating that children in both clinical groups had many gaps in their antibody repertoire that potentially leave them vulnerable to cerebral malaria in the follow-up period. Longitudinal studies have reported that individuals with antibodies targeting DBLβ domains with the DBLβ_motif_ have a reduced prospective risk of uncomplicated malaria [9] and high density parasitemia [13] and similar studies to determine the risk of cerebral malaria in the follow-up period would be informative.

## Conclusions

This study showed Fc features of antibodies targeting PfEMP1 domains could accurately distinguish between children with cerebral and uncomplicated malaria. Antibodies targeting specific PfEMP1 variants are likely involved in protection against cerebral malaria and PfEMP1 specific antibodies may mediate clearance of IE via C1q and FcγRIIIb dependent pathways, such as neutrophil phagocytosis, to protect children from cerebral malaria. Characterizing the antibody response to PfEMP1 may lead to the development of a multivalent PfEMP1-based vaccine or monoclonal antibody cocktail to protect from cerebral malaria by inducing a targeted functional immune response. Future validation of the antibody features identified in this study as correlates of protection may lead to the development of more sensitive prognostic indicators to identify populations and individuals who are susceptible to developing cerebral malaria.

## Supplementary Information

Additional file 1: Table 1**-**2, Figures S1–S11. Table 1–2 **-** Recombinant proteins used in multiplex assays. Fig S1 **-** General structure of PfEMP1. Fig S2**-** IgG responses measured in singleplex compared to multiplex. Fig S3 **-** Antigen specific IgG seropositivity. Fig S4–S5 - Correlation between age and antibody responses. Fig S6**-**S7 **-** ENLR with alpha tuning parameter set to 0.25 and 1. Fig S8–S9**-** Correlation between features most frequently selected by ENLR. Fig S10-S11 **-** Correlation between ADNP and ADCP of IE and antibody features targeting related recombinant proteins.

Additional file 2: Box and whisker plots comparing cerebral and uncomplicated malaria for grouped antigens.

Additional file 3: Box and whisker plots comparing cerebral and uncomplicated malaria for all variables.

## Data Availability

The data sets generated during the current study are available from the corresponding author on reasonable request.

## Abbreviations

ADCP: Antibody-dependent cellular phagocytosis
ADNP: Antibody-dependent neutrophil phagocytosis
AMA: Apical membrane antigen
AUROC: Area under the receiver operator curve
BCS: Blantyre coma score
BSA: Bovine serum albumin
CIDR: Cysteine-rich interdomain region
CSP: Circumsporozoite protein
DBL: Duffy binding like
DHE: Dihydroethidium bromide
EBA: Erythrocyte binding antigen
ENLR: Elastic net regularized logistic regression
EPCR: Endothelial protein C receptor
FcγR: Fcγ receptor
ICAM-1: Intercellular adhesion molecule-1
IE: Infected erythrocyte
Ig: Immunoglobulin
IQR: Interquartile range
MFI: Median fluorescence intensity
MSP: Merozoite surface protein
PBS: Phosphate buffered saline
PE: Phycoerythrin
*P. falciparum*: *Plasmodium falciparum*
PfEMP1: *Plasmodium falciparum* erythrocyte membrane protein 1
PLSR: Partial Least Squares Regression
SM: Severe Malaria
UM: Uncomplicated Malaria
