# Supplementary material for: A systems serology approach to identifying key antibody correlates of protection from cerebral malaria in Malawian children
Source: BMC Med. 2024 Sep 12;22:388. doi: 10.1186/s12916-024-03604-8 (PMC11396342; doi:10.1186/s12916-024-03604-8)
Supplement: Supplementary file 1 — Additional file 1: Table 1-2, Figures S1–S11. Table 1–2 - Recombinant proteins used in multiplex assays. Fig S1 - General structure of PfEMP1. Fig S2- IgG responses measured in singleplex compared to multiplex. Fig S3 - Antigen specific IgG seropositivity. Fig S4–S5 - Correlation between age and antibody responses. Fig S6-S7 - ENLR with alpha tuning parameter set to 0.25 and 1. Fig S8–S9- Correlation between features most frequently selected by ENLR. Fig S10-S11 - Correlation between ADNP and ADCP of IE and antibody features targeting related recombinant proteins. [file 12916_2024_3604_MOESM1_ESM.pdf]

Supplementary Table 1. Recombinant PfEMP1 proteins used in multiplex immunoassay

Protein identifier	Domain	Group	Associated DC/ gene	Predicted binding receptor	Expression system	Source
SM1	CIDRα2.4	B			WGCFS	*a*
SM2	CIDRβ1	B			WGCFS	*a*
SM3	DBLβ12	B/A	DC8		WGCFS	*a*
SM4	DBLβ3	A			WGCFS	*a*
SM5	DBLβ3	A	DC4		WGCFS	*a*
SM6	DBLδ1	B			WGCFS	*a*
SM8	DBLδ1	B			WGCFS	*a*
SM9	DBLδ1	B	DC8		WGCFS	*a*
SM11	DBLε3	B	DC11		WGCFS	*a*
SM12	DBLε9	B			WGCFS	*a*
SM14	DBLγ3	B	DC9?		WGCFS	*a*
SM15	DBLζ4	B	DC9?		WGCFS	*a*
SM17	DBLα1.5	A	DC4		WGCFS	*a*
SM18	CIDRα1.1	B/A	DC8	EPCR	WGCFS	*a*
SM19	CIDRα1.6	A	DC4	EPCR	WGCFS	*a*
SM22	DBLε5	*A*	*var*1		WGCFS	*a*
SM24	DBLζ3	B/A			WGCFS	*a*
SM25	DBLβ13	B			WGCFS	*a*
SM26	CIDRγ12	B			WGCFS	*a*
SM27	DBLδ7	A			WGCFS	*a*
SM28	CIDRα2.6DBLβ5	B			WGCFS	*a*
UM1	DBLα0.13	B			WGCFS	*a*
UM2	DBLδ1	B			WGCFS	*a*
UM8	DBLγ9	B/A			WGCFS	*a*
UM14	DBLδ1	B			WGCFS	*a*

UM19	DBLδ1	B			WGCFS	*a*
UM20	CIDRα3.1	B/C			WGCFS	*a*
UM45	CIDRα1.7	A			WGCFS	*a*
M1	DBLβ3*	A	PF11_0521_D4	ICAM-1	*Escherichia coli*	*b*
M6	DBLβ3*	A	3D7_Pfd1235w _D4	ICAM-1	*Escherichia coli*	*b*
M9	DBLβ1*	A	Dd2VAR32_D4	ICAM-1	*Escherichia coli*	*b*
CIDR–DBLβ3*	CIDRα1.6–DBLβ3*	A	3D7_Pfd1235w _D3-4	EPCR-ICAM-1	*Escherichia coli*	*b*
N27	DBLβ3	B	IT4VAR13_D4	ICAM-1	*Escherichia coli*	*b*
P1021	DBLβ3	B	KOB58843/ HB3var34	ICAM-1	*Escherichia coli*	*b*
P1014	DBLβ5	B	KOB63129/ HB3var21	ICAM-1	*Escherichia coli*	*b*
P1011	DBLβ5	B	AA75396/ Dd2var01a	ICAM-1	*Escherichia coli*	*b*
N30	DBLβ7	A	Dd2VAR52_D4		*Escherichia coli*	*b*
N31	DBLβ7	A	Hb3vAR01_D4		*Escherichia coli*	*b*
N32	DBLβ6	A	BT1983_D4		*Escherichia coli*	*b*

Table 1: (DC) Domain Cassette. (*) contains $DBL\beta_{motif}$ associated with dual EPCR-ICAM-1 binding IE. (WGCFS) wheat germ cell free system. (*a)* Proteins labelled SM and UM were derived from sequences of Papuan field isolates (1) and were expressed by Professor Takafumi Tsuboi and Dr. Eizo Takashima, Proteo-Science Center, Ehime University, Matsuyama, Ehime, Japan (2). (*b)* DBLβ proteins were supplied by Prof Anja Jensen, University of Copenhagen, Copenhagen, Denmark (3–5).

Supplementary Table 2. Recombinant merozoite and sporozoite proteins used in multiplex immunoassay

Protein	Expression system
AMA1	HEK293
MSP2 (FC27 allele)	*Escherichia coli*
MSP3	WGCFS
EBA175-RIII-V	*Escherichia coli*
CSP	HEK293

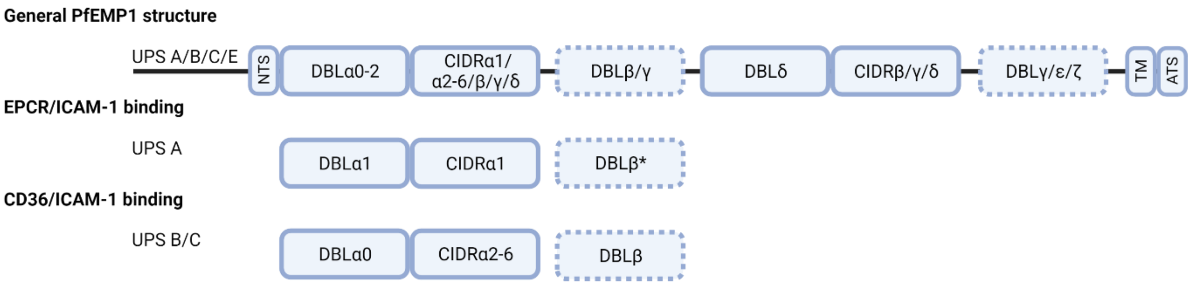

Figure S1. **General structure of PfEMP1.** PfEMP1 are classified based on the up stream promotor sequence (Groups A/B/C/E). The NTS is followed by a DBLα and CIDR domain head structure. Head structure CIDRα1 domains bind to EPCR and CIDRα2-6 bind to CD36. The head structure is followed by at least 2 domains, shown with solid borders, and longer PfEMP1 have additional domains, shown with dashed borders. Group A EPCR binding head structures can sometimes be followed by DBLβ with the ICAM-1 binding motif (*). Group B CD36 binding head structures can sometimes be followed by DBLβ that also bind ICAM-1 (but cannot be predicted by the motif). DBL: Duffy Binding Like; CIDR: Cysteine-rich interdomain Region; NTS: N-terminal sequence, TM: Transmembrane region; ATS: A-terminal sequence. Figure adapted from Smith *et al.* 2013, created with BioRender.com.

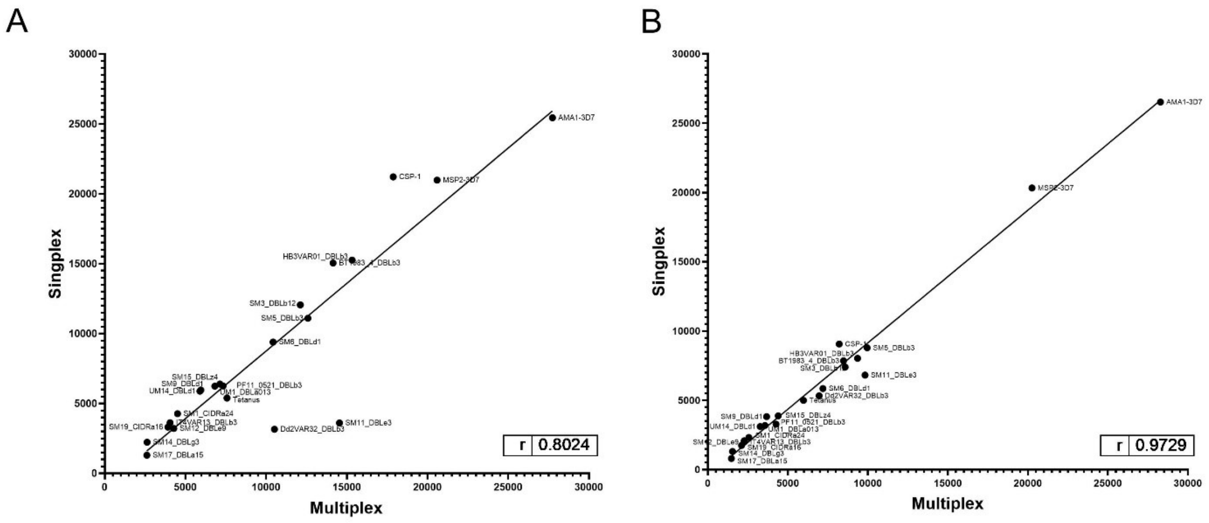

Figure S2. IgG responses for array of 17 antigens (one of two arrays) compared in single and multiplex formats, using serum pooled from 10 Malawian children, diluted by A) 1:50 and B) 1:100. r = Spearman's correlation coefficient. DBL = Duffy Binding Like domain. CIDR = Cysteine-Rich Interdomain Region. a = alpha, b = beta, d = delta, e = epsilon, g = gamma, z = zeta.

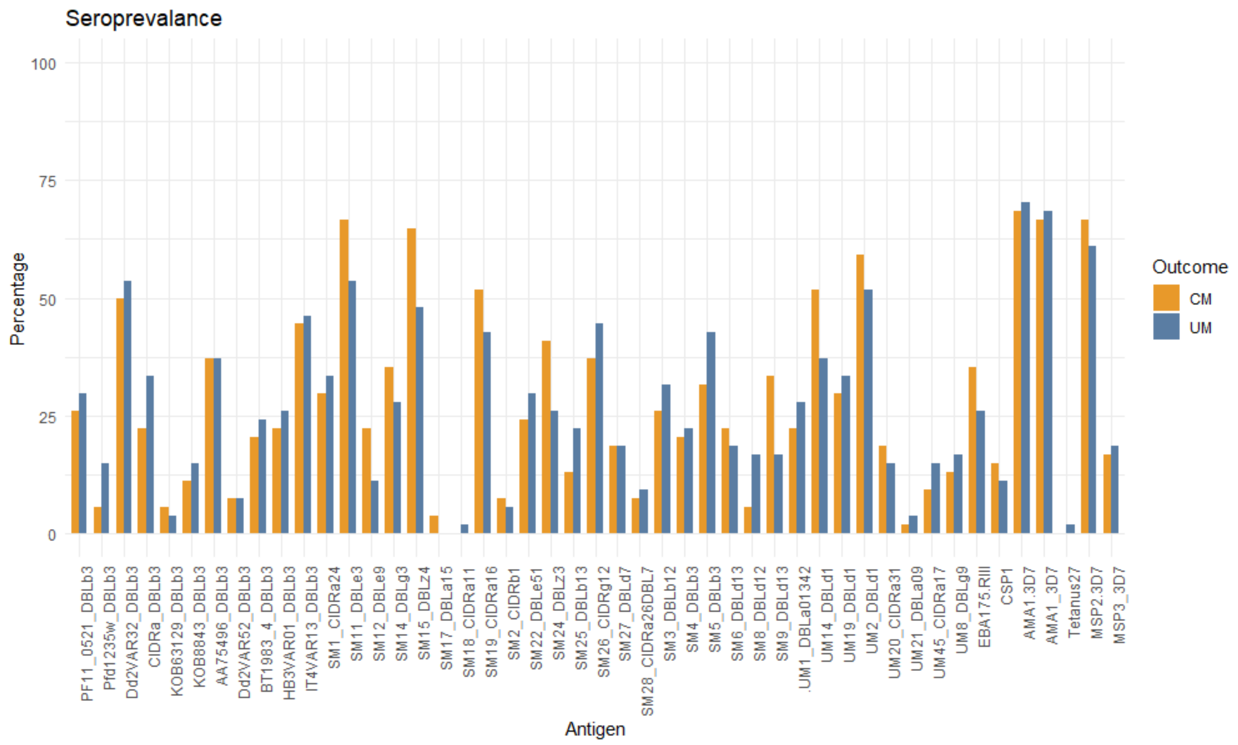

Figure S3. Percentage children with cerebral malaria (orange) or uncomplicated malaria (blue) who are seropositive to PfEMP1 antigens. Seropositivity was defined as IgG titers greater than 2 SD above the mean of malaria naïve individuals from Melbourne, Australia. DBL = Duffy Binding Like domain. CIDR = Cysteine-Rich Interdomain Region. a = alpha, b = beta, d = delta, e = epsilon, g = gamma, z = zeta.

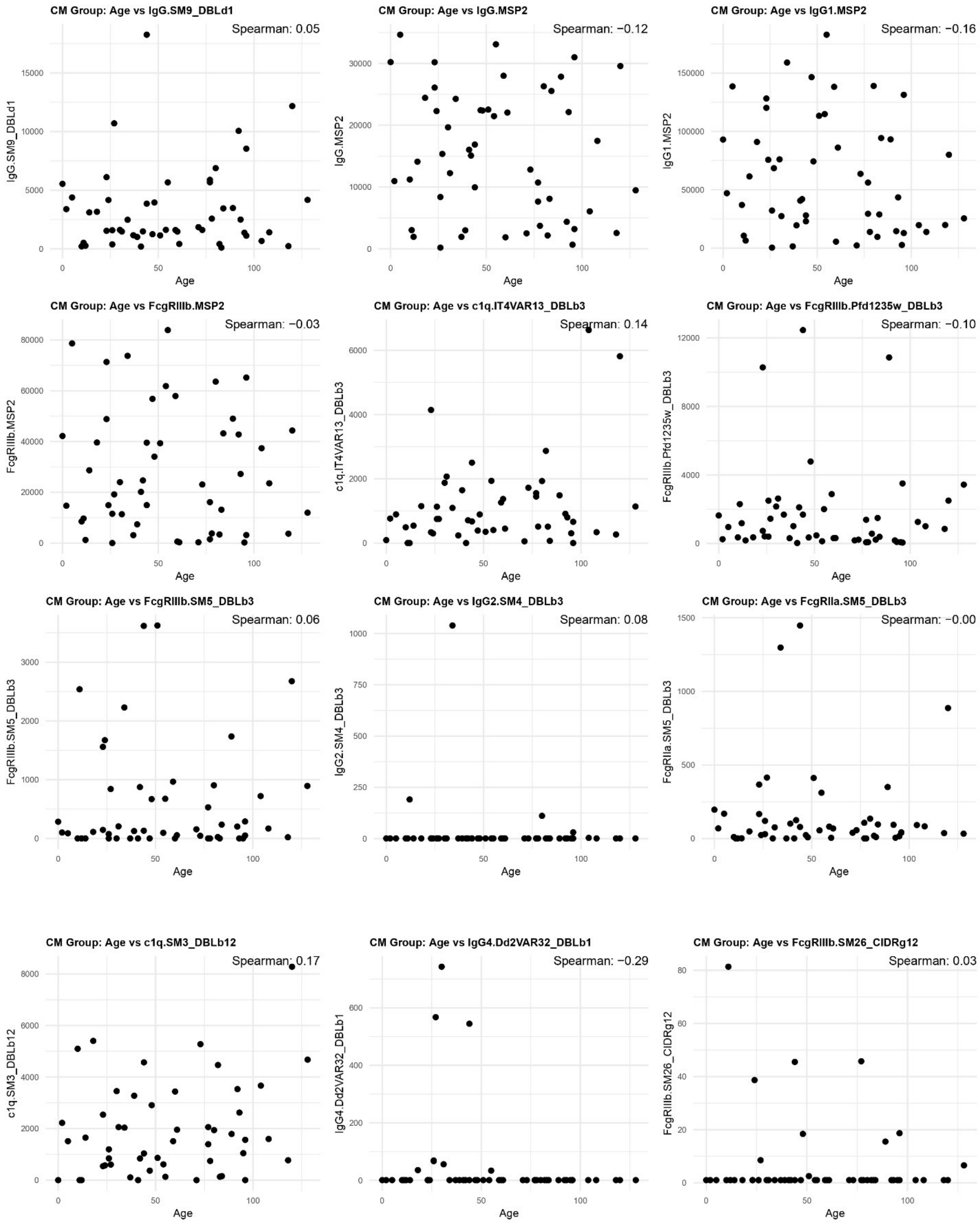

(continued on following page)

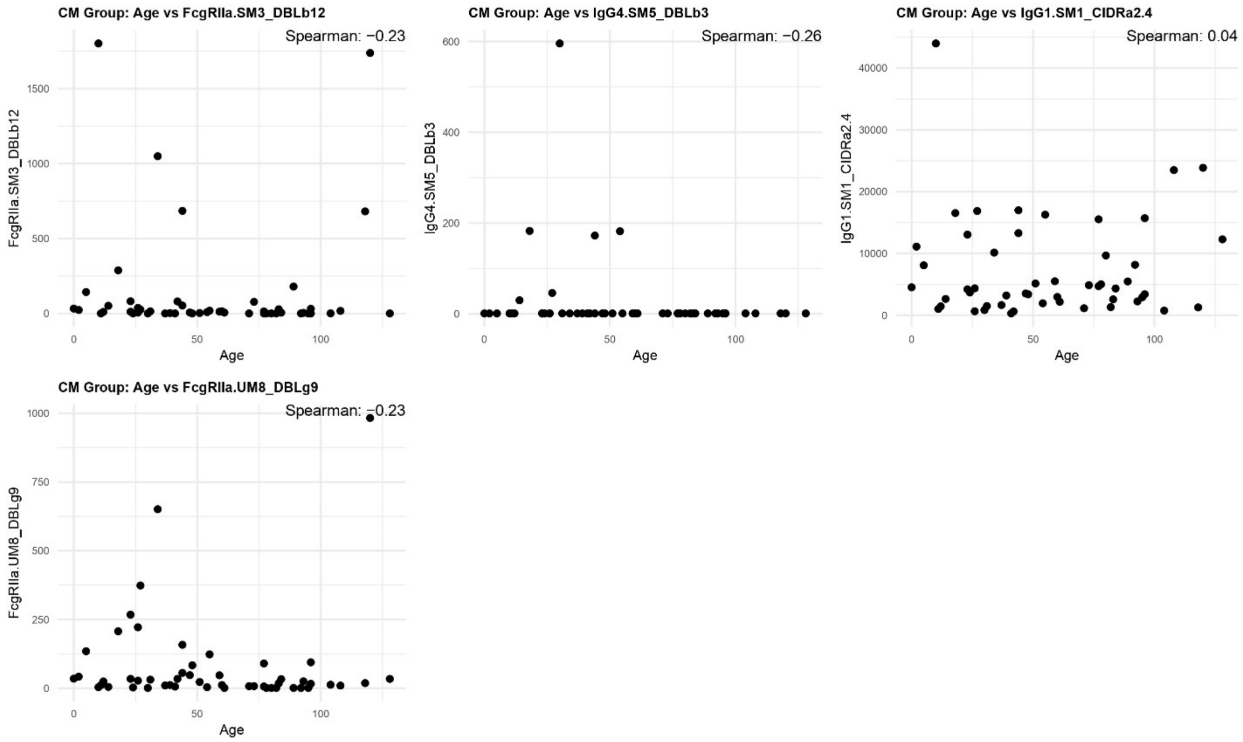

Figure S4: Correlation between age (months) and antibody response (median fluorescence intensity, measured by multiplex immunoassay) amongst children with cerebral malaria (CM), for antibody features found to be significantly and by greater than 2 folds different in children with cerebral and uncomplicated malaria. Strength of correlation represented by Spearman correlation coefficient, R (Spearman). DBL = Duffy Binding Like domain. CIDR = Cysteine-Rich Interdomain Region. a = alpha, b = beta, d = delta, e = epsilon, g = gamma, z = zeta.

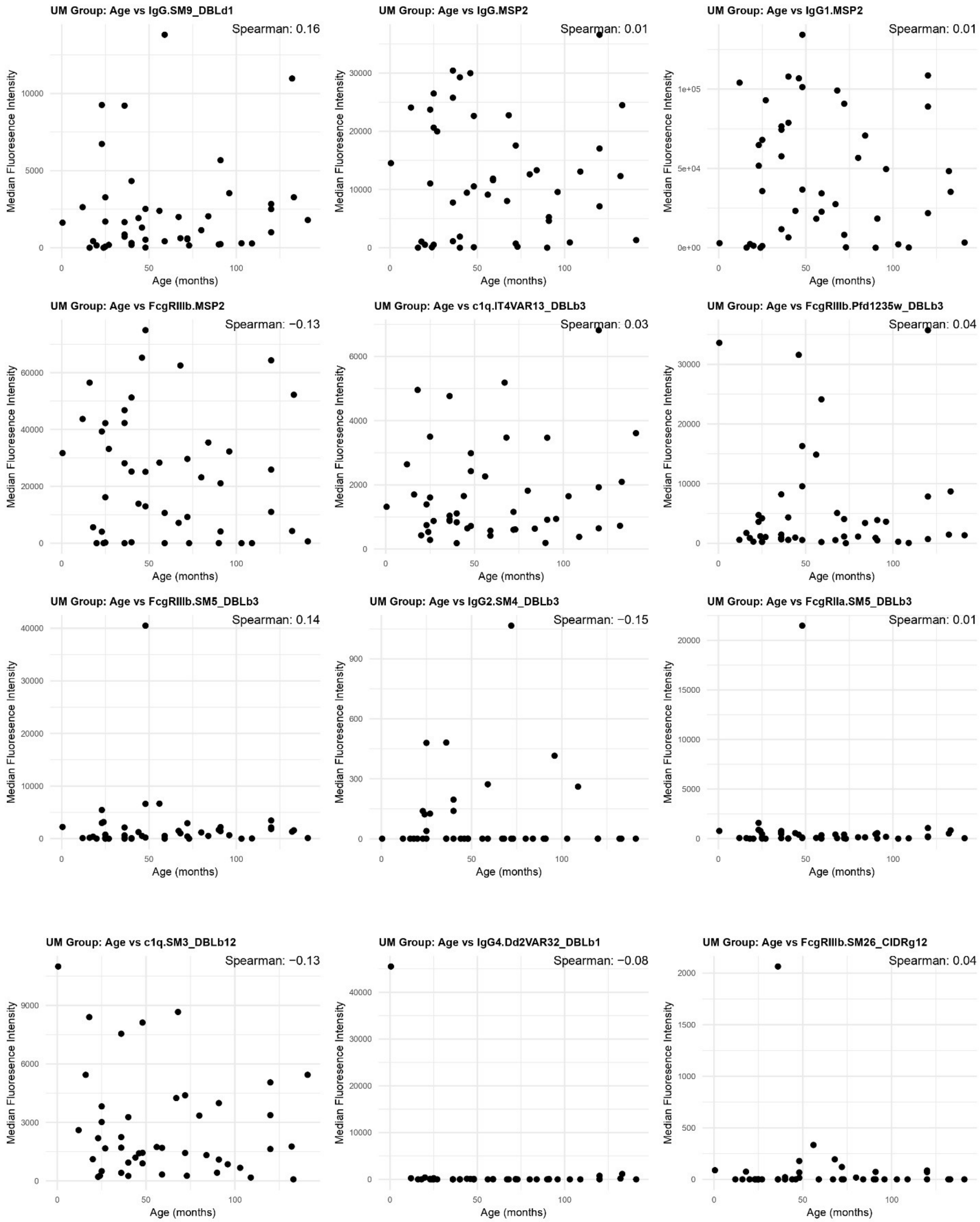

(continued on following page)

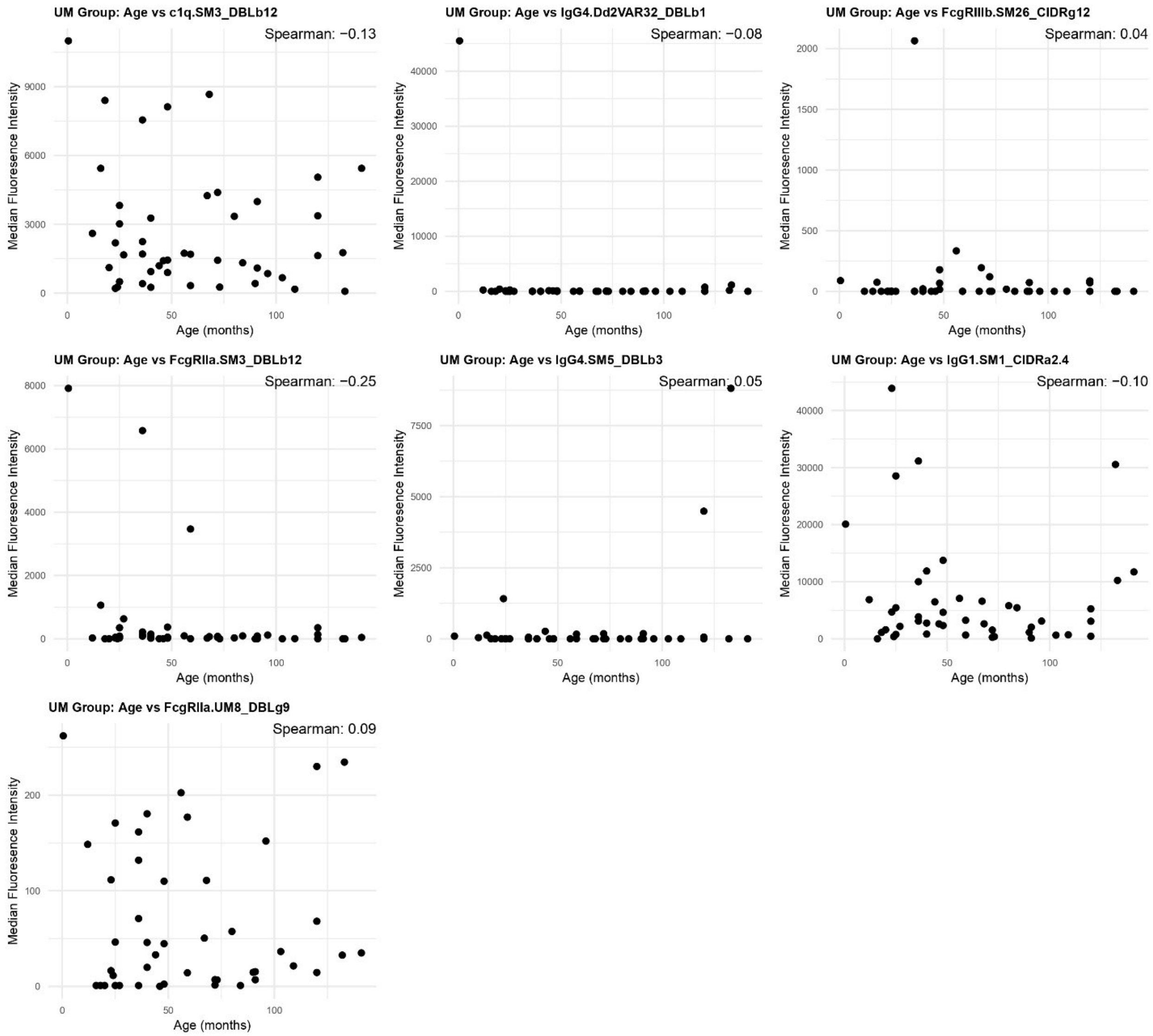

Figure S5: Correlation between age (months) and antibody response (median fluorescence intensity, measured by multiplex immunoassay) amongst children with uncomplicated malaria (UM), for antibody features found to be significantly and by greater than 2 folds different in children with cerebral and uncomplicated malaria. Strength of correlation represented by Spearman correlation coefficient, R (Spearman). DBL = Duffy Binding Like domain. CIDR = Cysteine-Rich Interdomain Region. a = alpha, b = beta, d = delta, e = epsilon, g = gamma, z = zeta.

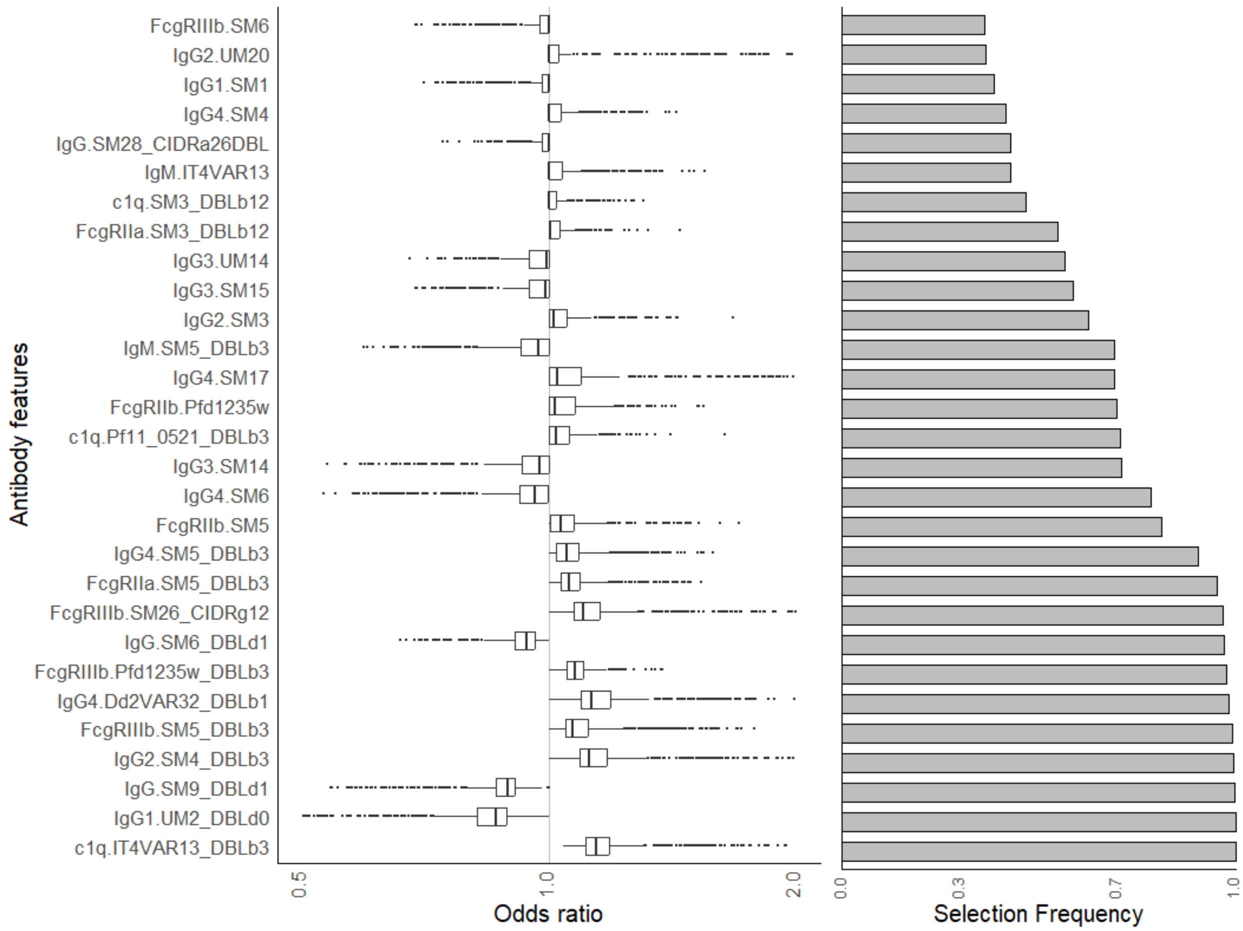

Figure S6. Odds ratio of antibody features from 5,000 repeats of 10-fold cross validated ENET regression models using alpha tuning parameter of 0.25. Variables shown in order of selection frequency (top 29 most selected variables are shown). Features with mean odds ratio greater than 1 represent responses associated with increased odds of uncomplicated malaria and features with mean odds ratio less than 1 are associated with increased odds of cerebral malaria. DBL = Duffy Binding Like domain. CIDR = Cysteine-Rich Interdomain Region. a = alpha, b = beta, d = delta, e = epsilon, g = gamma, z = zeta.

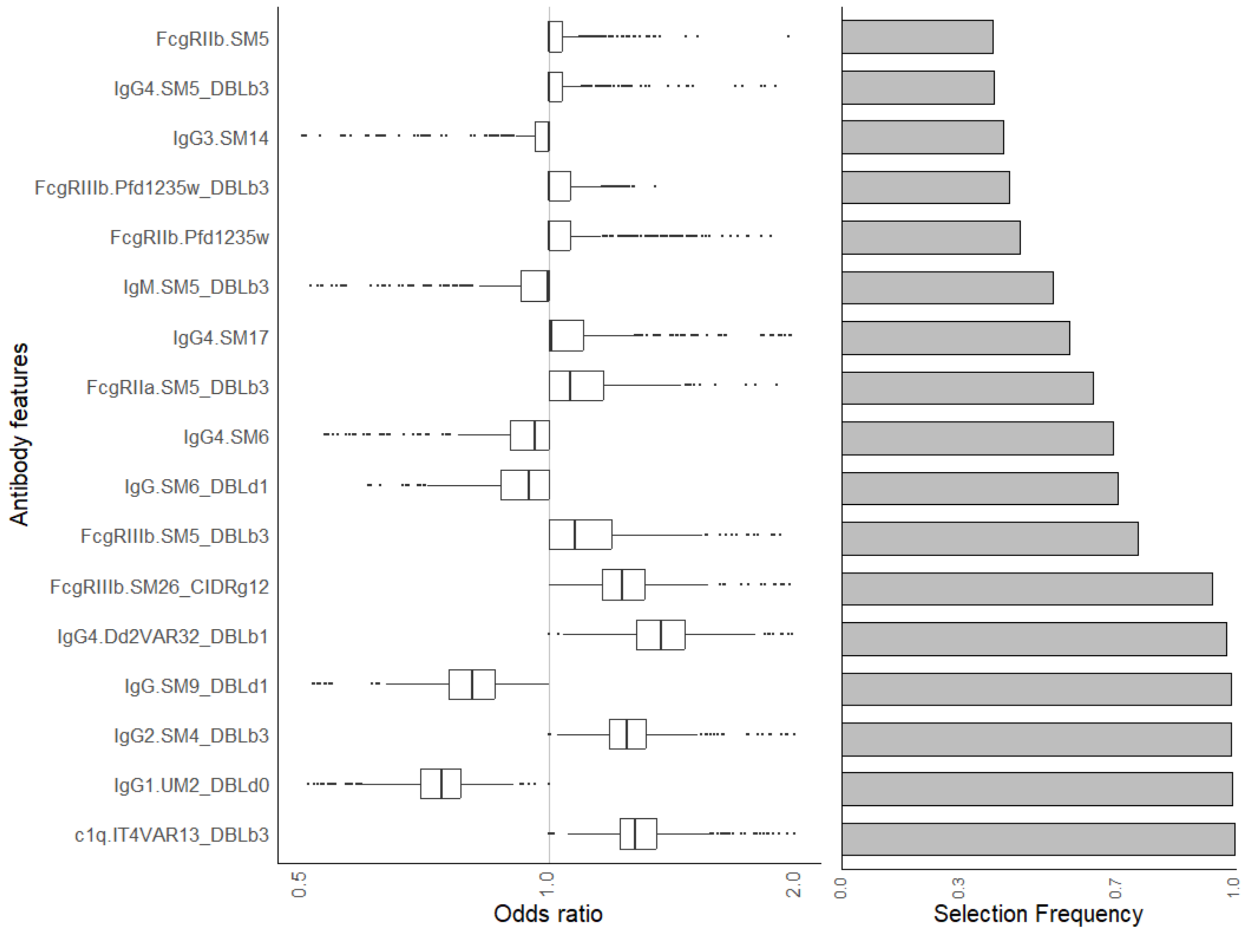

Figure S7. Odds ratio of antibody features from 5,000 repeats of 10-fold cross validated ENET regression models using alpha tuning parameter of 1. Variables shown in order of selection frequency (top 17 most selected variables are shown). Features with mean odds ratio greater than 1 represents responses associated with increased odds of uncomplicated malaria and features with mean odds ratio less than 1 are associated with increased odds of cerebral malaria. DBL = Duffy Binding Like domain. CIDR = Cysteine-Rich Interdomain Region. a = alpha, b = beta, d = delta, e = epsilon, g = gamma, z = zeta.

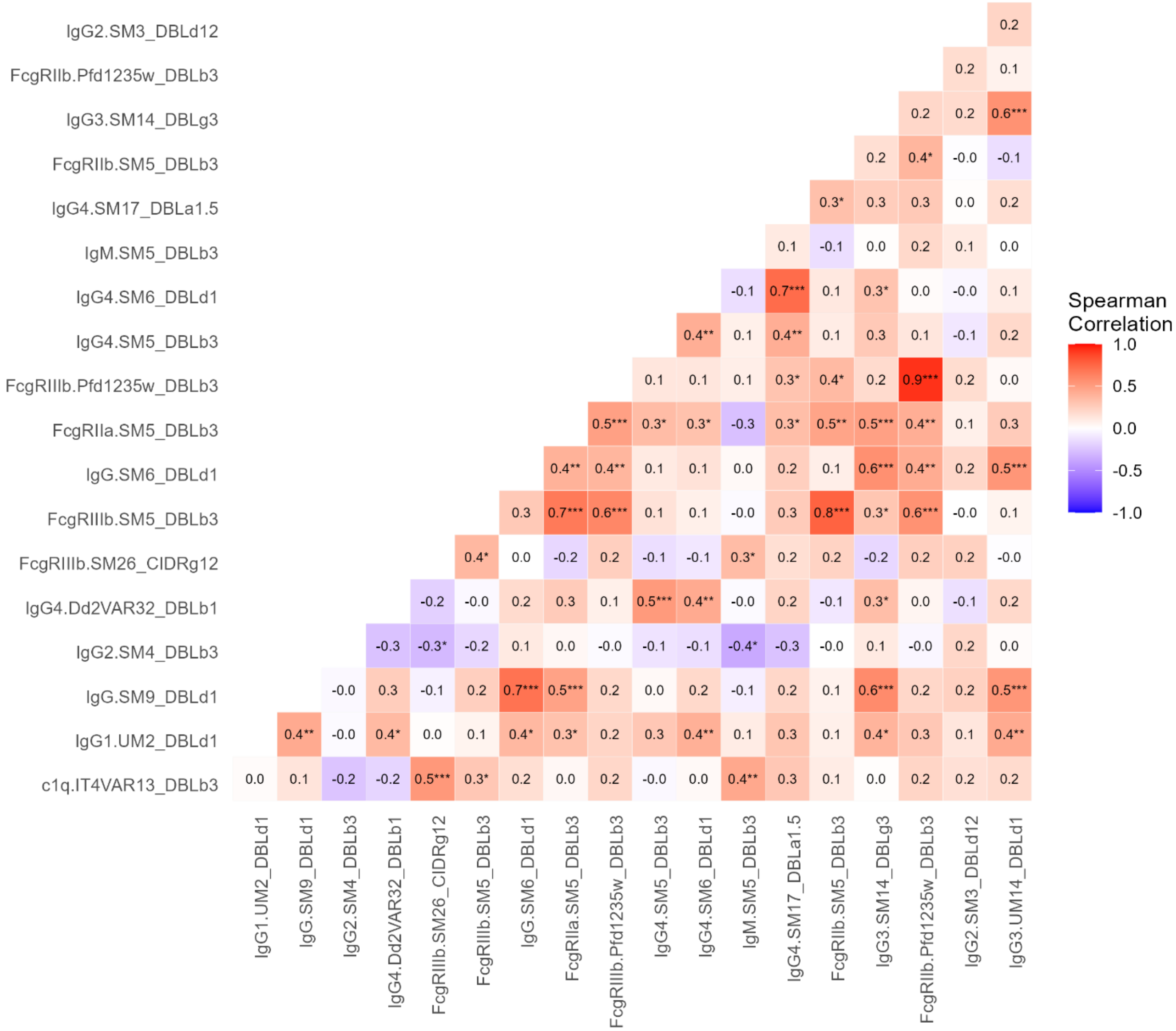

Figure S8. Spearman correlation of features that appeared in >70% of ENLR model iterations amongst children with uncomplicated malaria. Negative correlations are shaded blue and positive correlations in red. DBL = Duffy Binding Like domain. CIDR = Cysteine-Rich Interdomain Region. a = alpha, b = beta, d = delta, e = epsilon, g = gamma, z = zeta. * p-value < 0.05, ** p-value < 0.01, *** p-value <0.001.

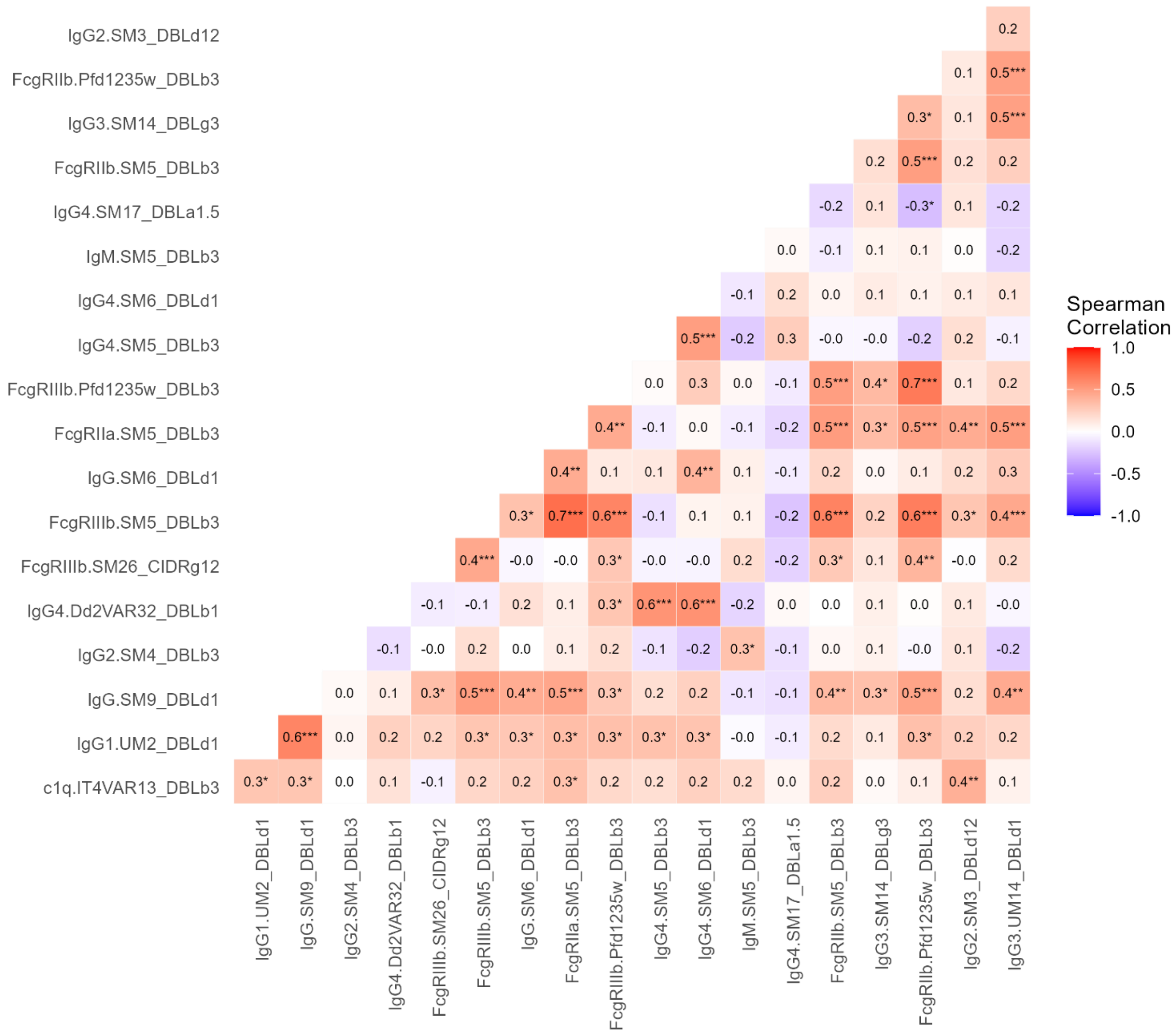

Figure S9. Spearman correlation of features that appeared in >70% of ENLR model iterations amongst children with cerebral malaria. Negative correlations are shaded blue and positive correlations in red. DBL = Duffy Binding Like domain. CIDR = Cysteine-Rich Interdomain Region. a = alpha, b = beta, d = delta, e = epsilon, g = gamma, z = zeta. * p-value < 0.05, ** p-value < 0.01, *** p-value <0.001.

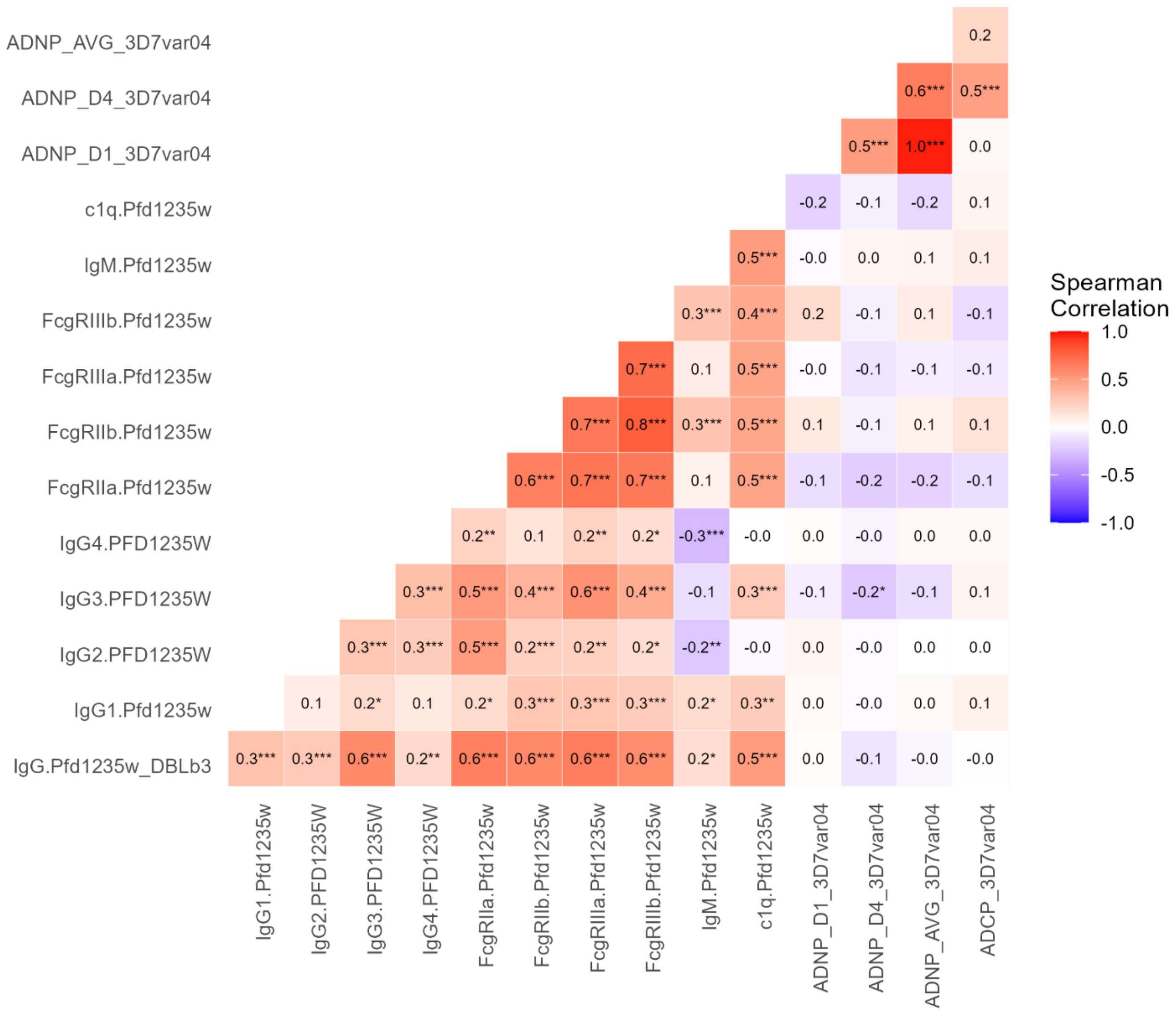

Figure S10. Correlations between antibody features targeting DBLβ3_Pfd1235w and antibody dependent phagocytosis (ADNP and ADCP) of IE expressing the corresponding PfEMP1, 3D7VAR04. Circle size and shade represents Spearman's correlation co-efficient, r. Negative correlations are shaded blue and positive correlations in red. ADNP_D1 and ADNP_D4 show ADNP from two individual neutrophil donors and ADNP_AVG shows the average ADNP of the two donors. ADCP: antibody dependent THP-1 cell phagocytosis. DBL = Duffy Binding Like domain. CIDR = Cysteine-Rich Interdomain Region. a = alpha, b = beta, d = delta, e = epsilon, g = gamma, z = zeta. * p-value < 0.05, ** p-value < 0.01, *** p-value <0.001.

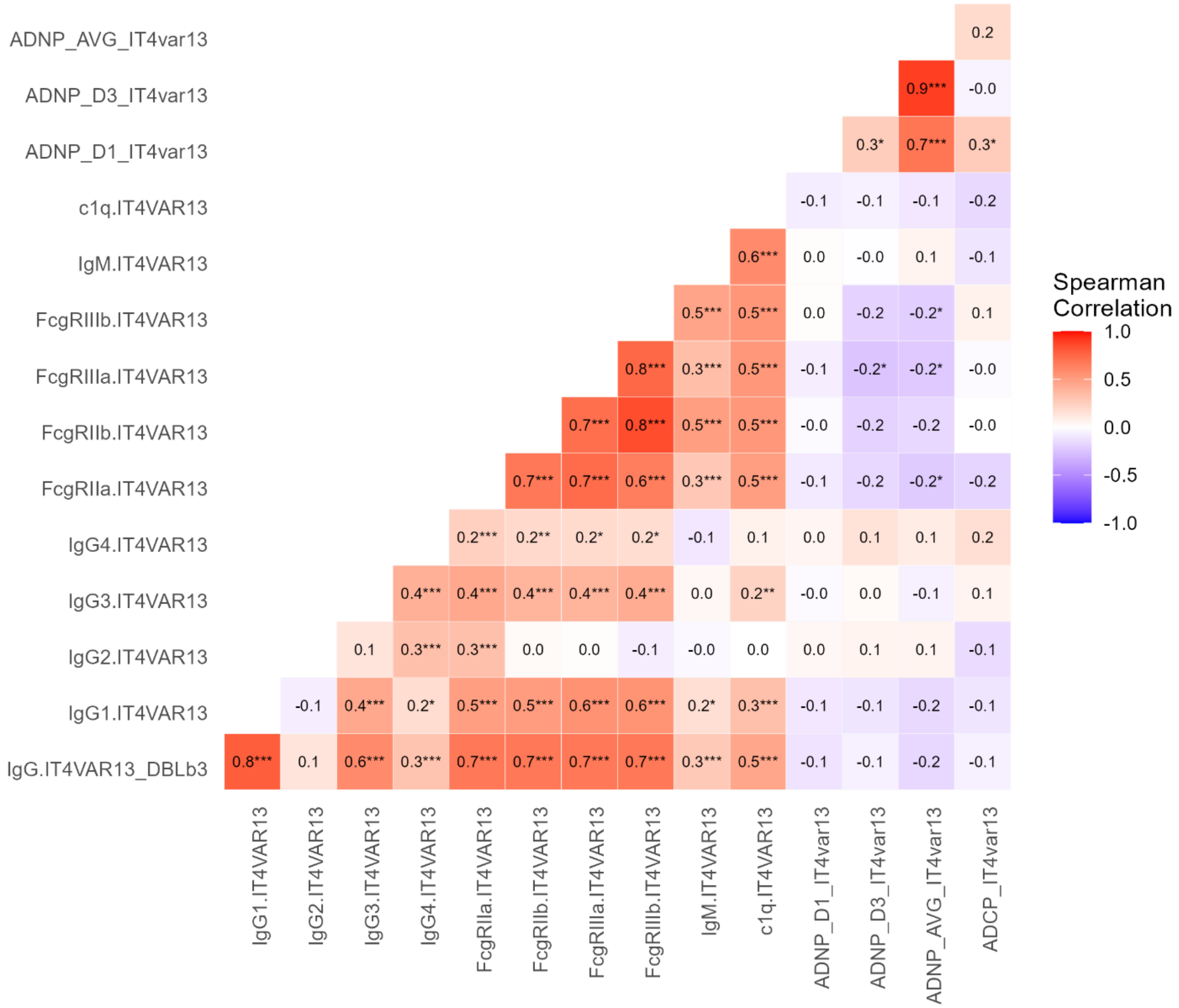

Figure S11. Correlations between antibody features targeting DBLβ3_IT4VAR13 and antibody dependent neutrophil phagocytosis (ADNP and ADCP) of IE the corresponding PfEMP1, IT4VAR13. Circle size and shade represents Spearman's correlation co-efficient, r. Negative correlations are shaded blue and positive correlations in red. ADNP_D1 and ADNP_D3 show ADNP from two individual neutrophil donors and ADNP_AVG shows the average ADNP of the two donors. ADCP: antibody dependent THP-1 cell phagocytosis. DBL = Duffy Binding Like domain. CIDR = Cysteine-Rich Interdomain Region. a = alpha, b = beta, d = delta, e = epsilon, g = gamma, z = zeta. * p-value < 0.05, ** p-value < 0.01, *** p-value <0.001.

References:

1. Tonkin-Hill GQ, Trianty L, Noviyanti R, Nguyen HHT, Sebayang BF, Lampah DA, et al. The Plasmodium falciparum transcriptome in severe malaria reveals altered expression of genes involved in important processes including surface antigen–encoding var genes. PLoS Biol. 2018;16:e2004328.

2. Rambhatla JS, Tonkin-hill GQ, Takashima E, Tsuboi T, Noviyanti R, Trianty L, et al. Identifying Targets of Protective Antibodies against Severe Malaria in Papua, Indonesia, Using Locally Expressed Domains. Infect Immun. 2022;90:e0043521.

3. Olsen RW, Ecklu-Mensah G, Bengtsson A, Ofori MF, Lusingu JP, Castberg FC, et al. Natural and Vaccine-Induced Acquisition of Cross-Reactive IgG-Inhibiting ICAM-1-Specific Binding of a Plasmodium falciparum PfEMP1 Subtype Associated Specifically with Cerebral Malaria. Infect Immun. 2018;86:1–17.

4. Olsen RW, Ecklu-Mensah G, Bengtsson A, Ofori MF, Kusi KA, Koram KA, et al. Acquisition of IGG to ICAM-1-Binding DBLB Domains in the plasmodium falciparum erythrocyte membrane protein 1 antigen family varies between groups A, B, and C. Infect Immun. 2019;87:e00224-19.

5. Bengtsson A, Joergensen L, Rask TS, Olsen RW, Andersen MA, Turner L, et al. A Novel Domain Cassette Identifies Plasmodium falciparum PfEMP1 Proteins Binding ICAM-1 and Is a Target of Cross-Reactive, Adhesion-Inhibitory Antibodies. J Immunol. 2013;190:240–9.
