# Supplementary material for: A systems serology approach to identifying key antibody correlates of protection from cerebral malaria in Malawian children
Source: BMC Med. 2024 Sep 12;22:388. doi: 10.1186/s12916-024-03604-8 (PMC11396342; doi:10.1186/s12916-024-03604-8)
Supplement: Supplementary file 2 — Additional file 2: Box and whisker plots comparing cerebral and uncomplicated malaria for grouped antigens. [file 12916_2024_3604_MOESM2_ESM.pdf]

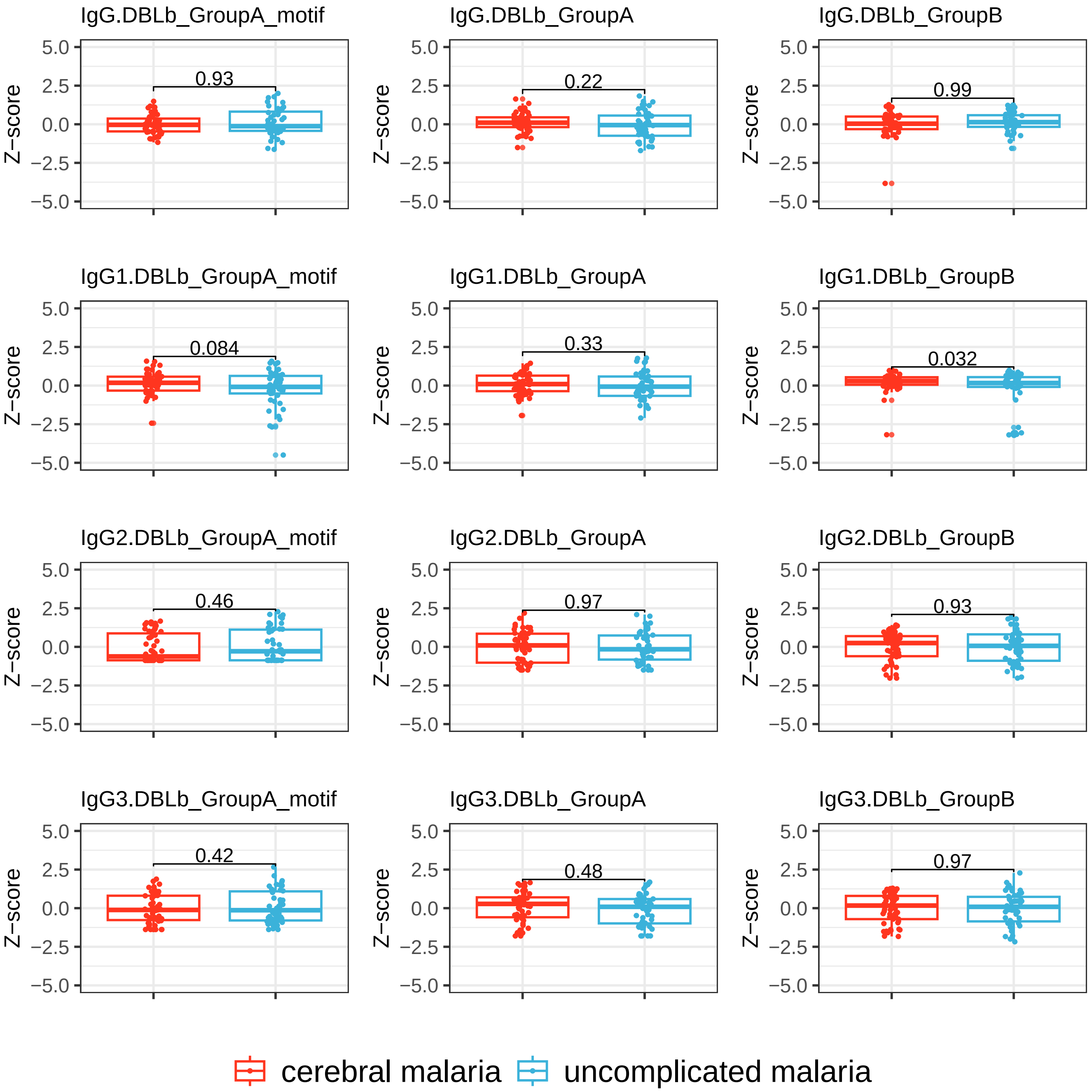
IgG.DBLb_GroupA_motif
IgG.DBLb_GroupA
IgG.DBLb_GroupB
IgG1.DBLb_GroupA_motif
IgG1.DBLb_GroupA
IgG1.DBLb_GroupB
IgG2.DBLb_GroupA_motif
IgG2.DBLb_GroupA
IgG2.DBLb_GroupB
IgG3.DBLb_GroupA_motif
IgG3.DBLb_GroupA
IgG3.DBLb_GroupB
Z−score
5.0
2.5
0.0
−2.5
−5.0
0.93
0.22
0.99
0.084
0.33
0.032
0.46
0.97
0.93
0.42
0.48
0.97
cerebral malaria
uncomplicated malaria

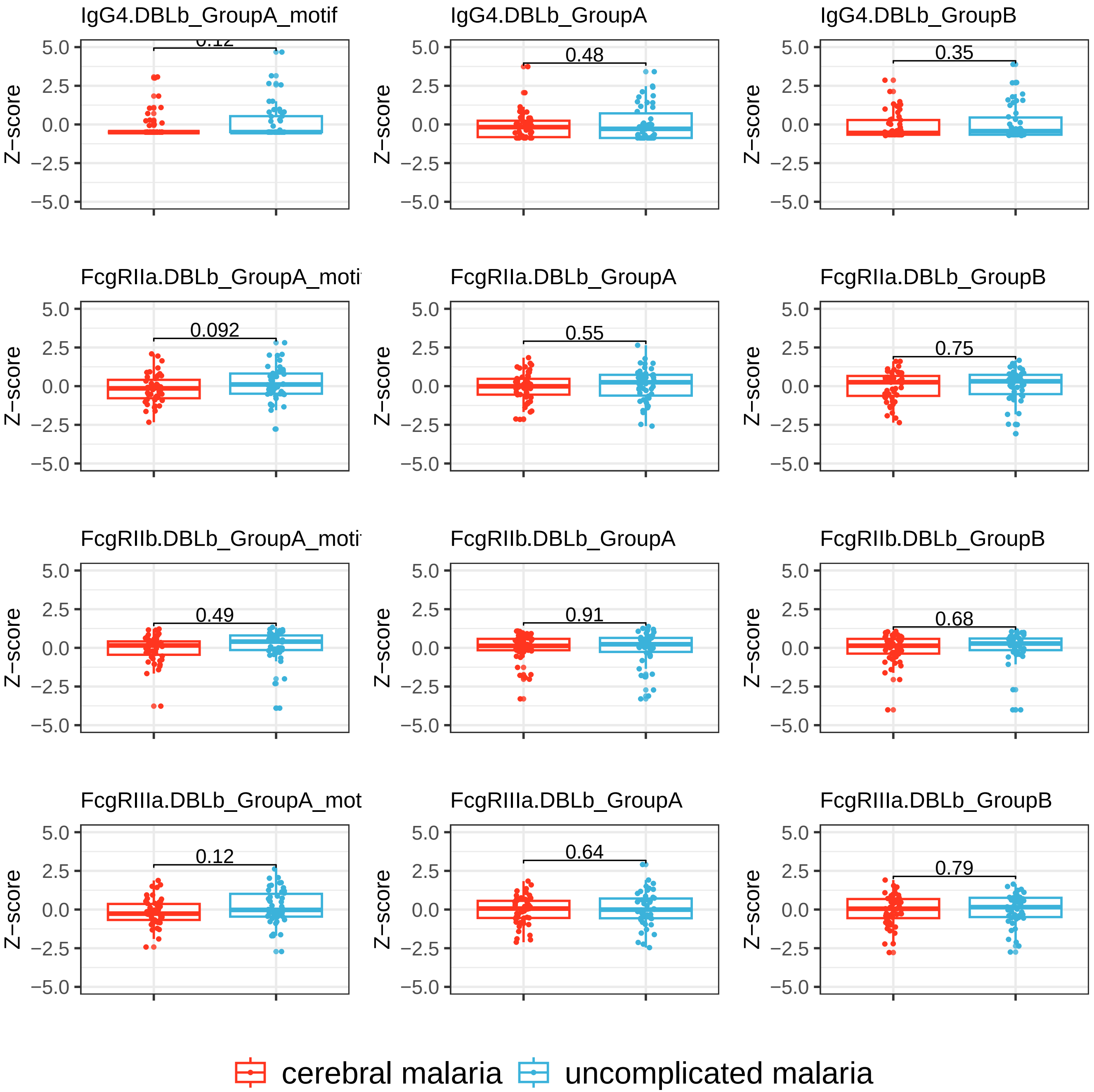

IgG4.DBLb_GroupA_motif
IgG4.DBLb_GroupA
IgG4.DBLb_GroupB
0.48
0.35
FcgRIIa.DBLb_GroupA_moti
FcgRIIa.DBLb_GroupA
FcgRIIa.DBLb_GroupB
0.092
0.55
0.75
FcgRIIb.DBLb_GroupA_moti
FcgRIIb.DBLb_GroupA
FcgRIIb.DBLb_GroupB
0.49
0.91
0.68
FcgRIIIa.DBLb_GroupA_mot
FcgRIIIa.DBLb_GroupA
FcgRIIIa.DBLb_GroupB
0.12
0.64
0.79
Z−score
5.0
2.5
0.0
−2.5
−5.0
cerebral malaria
uncomplicated malaria

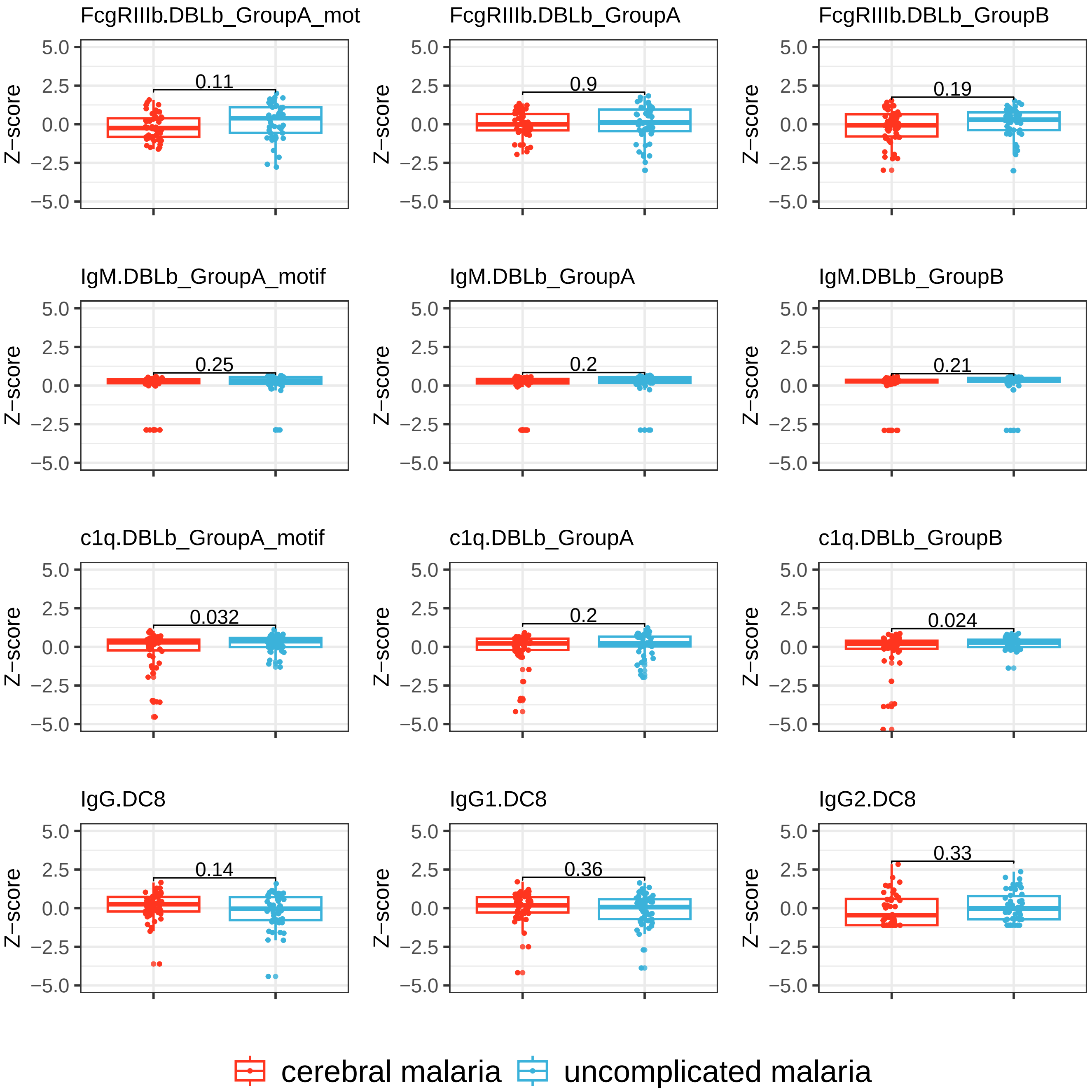

FcgRIIIb.DBLb_GroupA_mot
FcgRIIIb.DBLb_GroupA
FcgRIIIb.DBLb_GroupB
IgM.DBLb_GroupA_motif
IgM.DBLb_GroupA
IgM.DBLb_GroupB
c1q.DBLb_GroupA_motif
c1q.DBLb_GroupA
c1q.DBLb_GroupB
IgG.DC8
IgG1.DC8
IgG2.DC8
Z−score
0.11
0.9
0.19
0.25
0.2
0.21
0.032
0.2
0.024
0.14
0.36
0.33
5.0
2.5
0.0
−2.5
−5.0
cerebral malaria
uncomplicated malaria

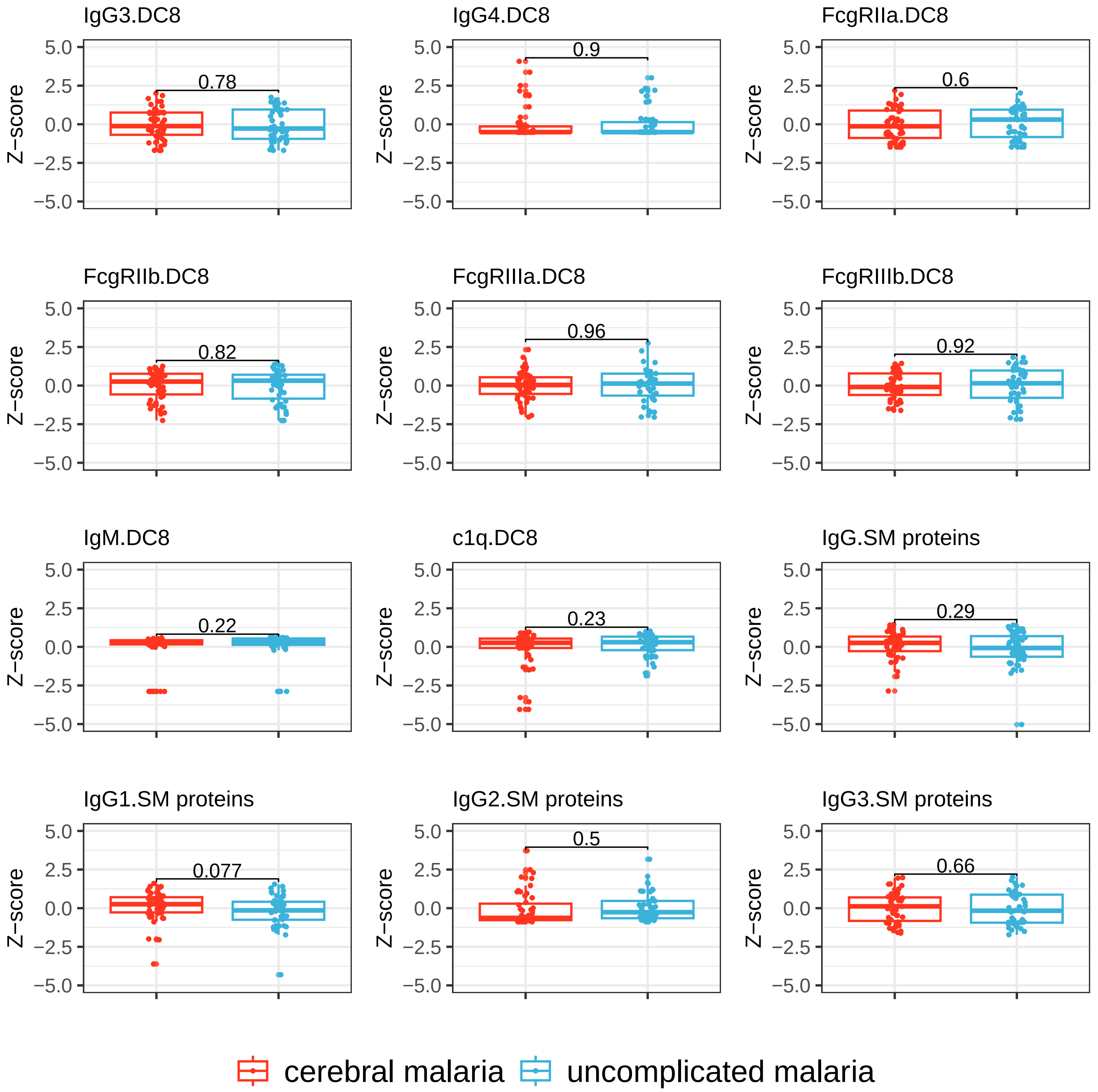
IgG3.DC8
0.78
IgG4.DC8
0.9
FcgRIIa.DC8
0.6
FcgRIIb.DC8
0.82
FcgRIIIa.DC8
0.96
FcgRIIIb.DC8
0.92
IgM.DC8
0.22
c1q.DC8
0.23
IgG.SM proteins
0.29
IgG1.SM proteins
0.077
IgG2.SM proteins
0.5
IgG3.SM proteins
0.66
Z−score
5.0
2.5
0.0
−2.5
−5.0
cerebral malaria
uncomplicated malaria

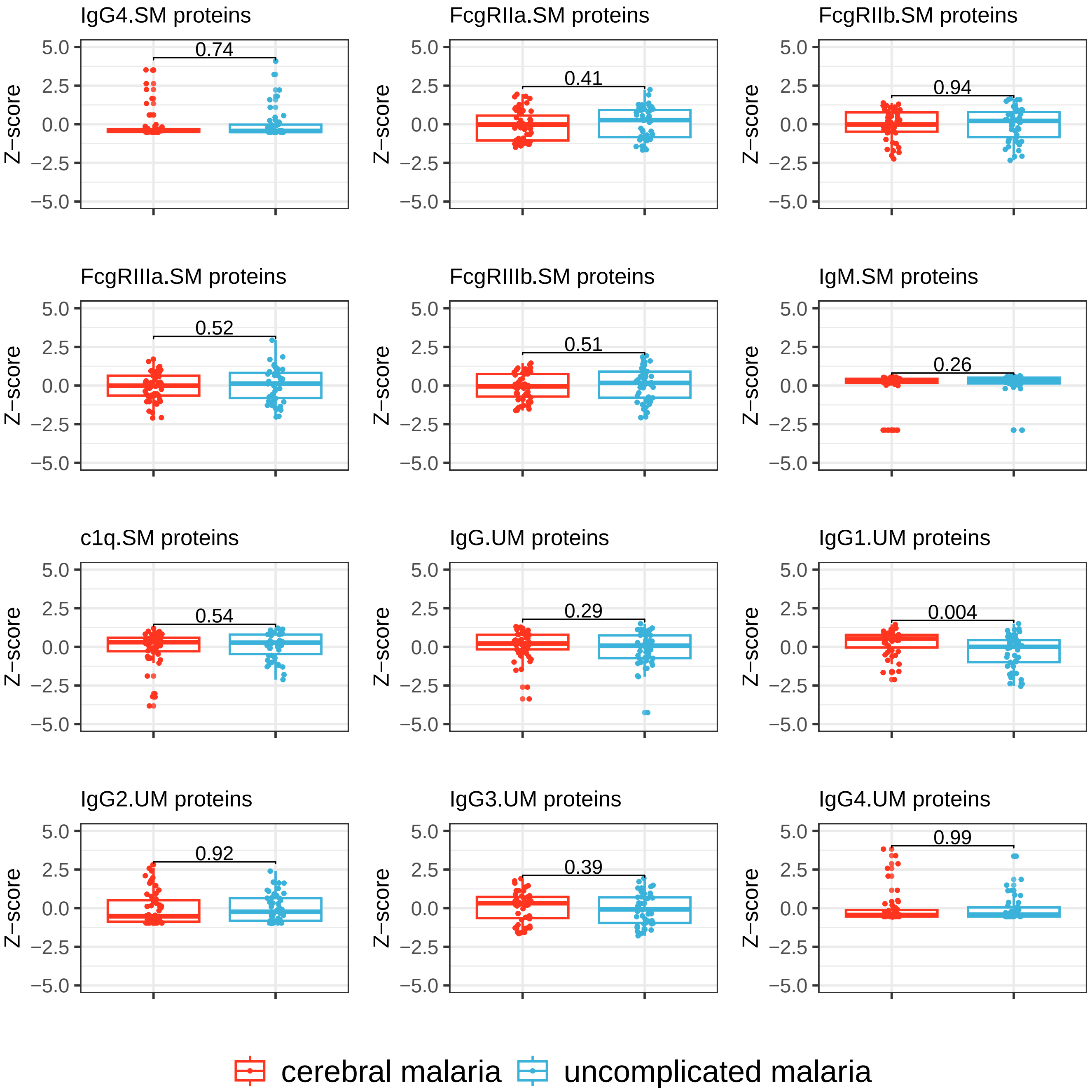
IgG4.SM proteins
0.74
FcgRIIa.SM proteins
0.41
FcgRIIb.SM proteins
0.94
FcgRIIIa.SM proteins
0.52
FcgRIIIb.SM proteins
0.51
IgM.SM proteins
0.26
c1q.SM proteins
0.54
IgG.UM proteins
0.29
IgG1.UM proteins
0.004
IgG2.UM proteins
0.92
IgG3.UM proteins
0.39
IgG4.UM proteins
0.99
Z−score
5.0
2.5
0.0
−2.5
−5.0
cerebral malaria
uncomplicated malaria

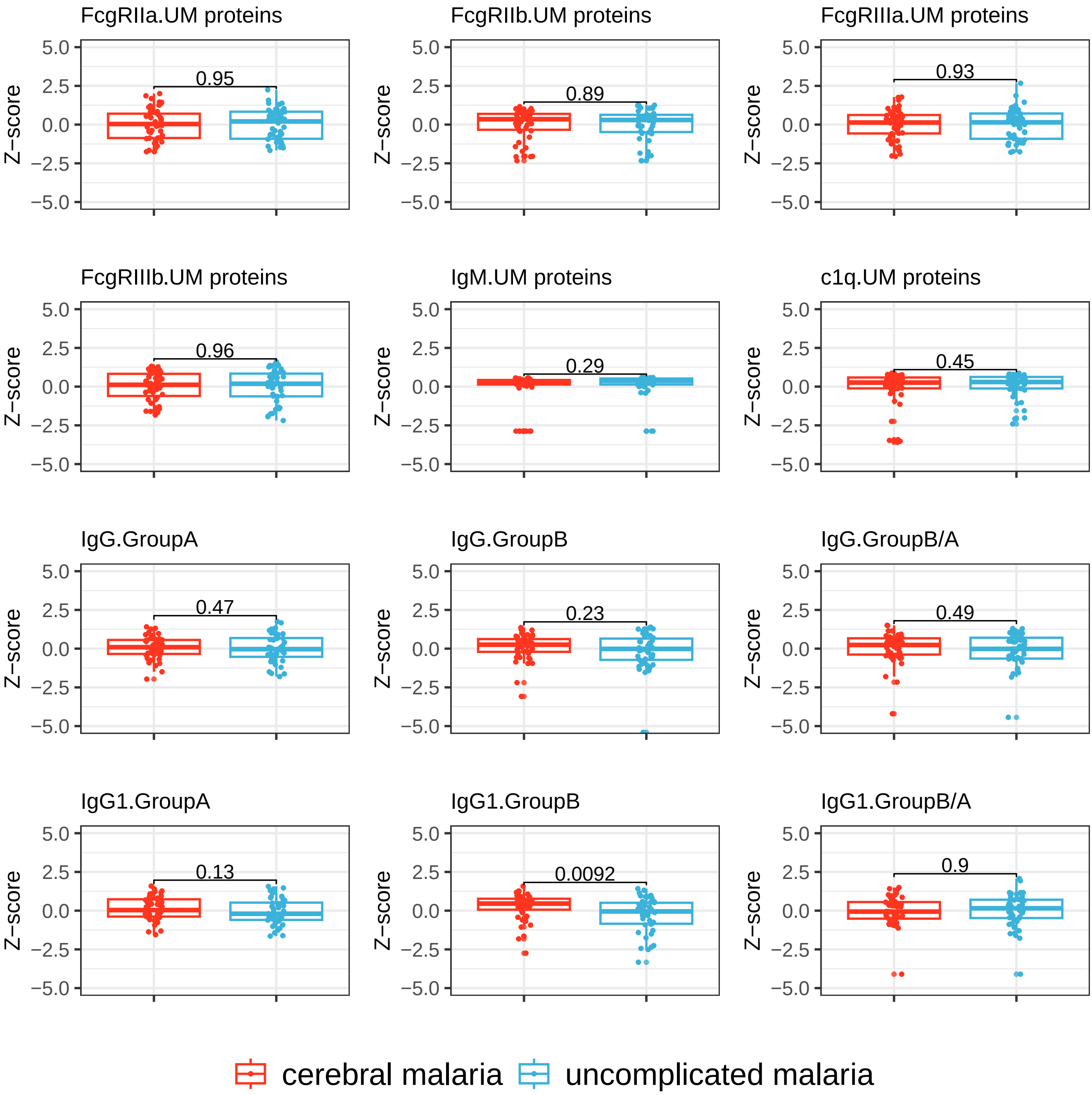
FcgRIIa.UM proteins
0.95
FcgRIIb.UM proteins
0.89
FcgRIIIa.UM proteins
0.93
FcgRIIIb.UM proteins
0.96
IgM.UM proteins
0.29
c1q.UM proteins
0.45
IgG.GroupA
0.47
IgG.GroupB
0.23
IgG.GroupB/A
0.49
IgG1.GroupA
0.13
IgG1.GroupB
0.0092
IgG1.GroupB/A
0.9
Z−score
5.0
2.5
0.0
−2.5
−5.0
cerebral malaria
uncomplicated malaria

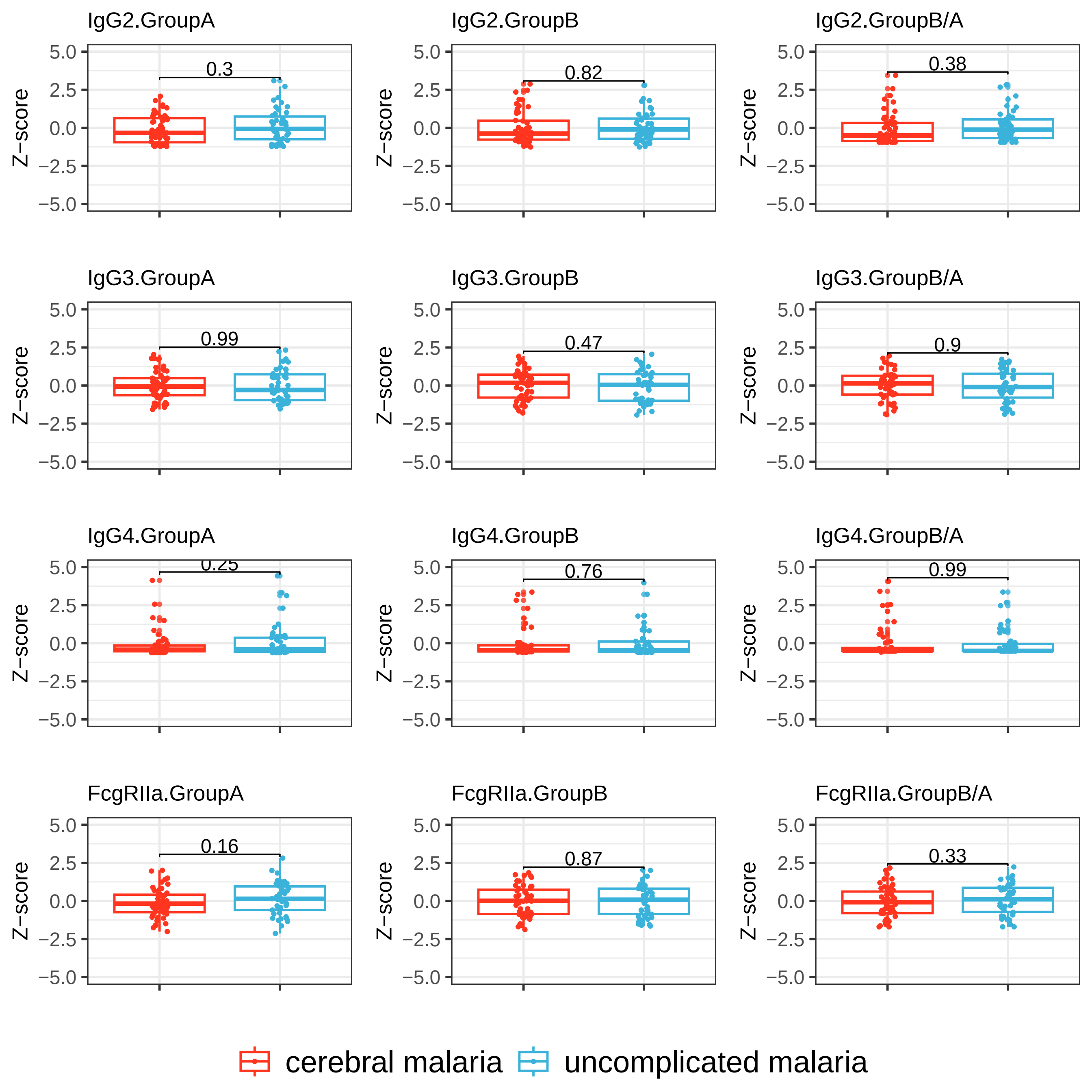
IgG2.GroupA
0.3
IgG2.GroupB
0.82
IgG2.GroupB/A
0.38
IgG3.GroupA
0.99
IgG3.GroupB
0.47
IgG3.GroupB/A
0.9
IgG4.GroupA
0.25
IgG4.GroupB
0.76
IgG4.GroupB/A
0.99
FcgRIIa.GroupA
0.16
FcgRIIa.GroupB
0.87
FcgRIIa.GroupB/A
0.33
Z−score
5.0
2.5
0.0
−2.5
−5.0
cerebral malaria
uncomplicated malaria

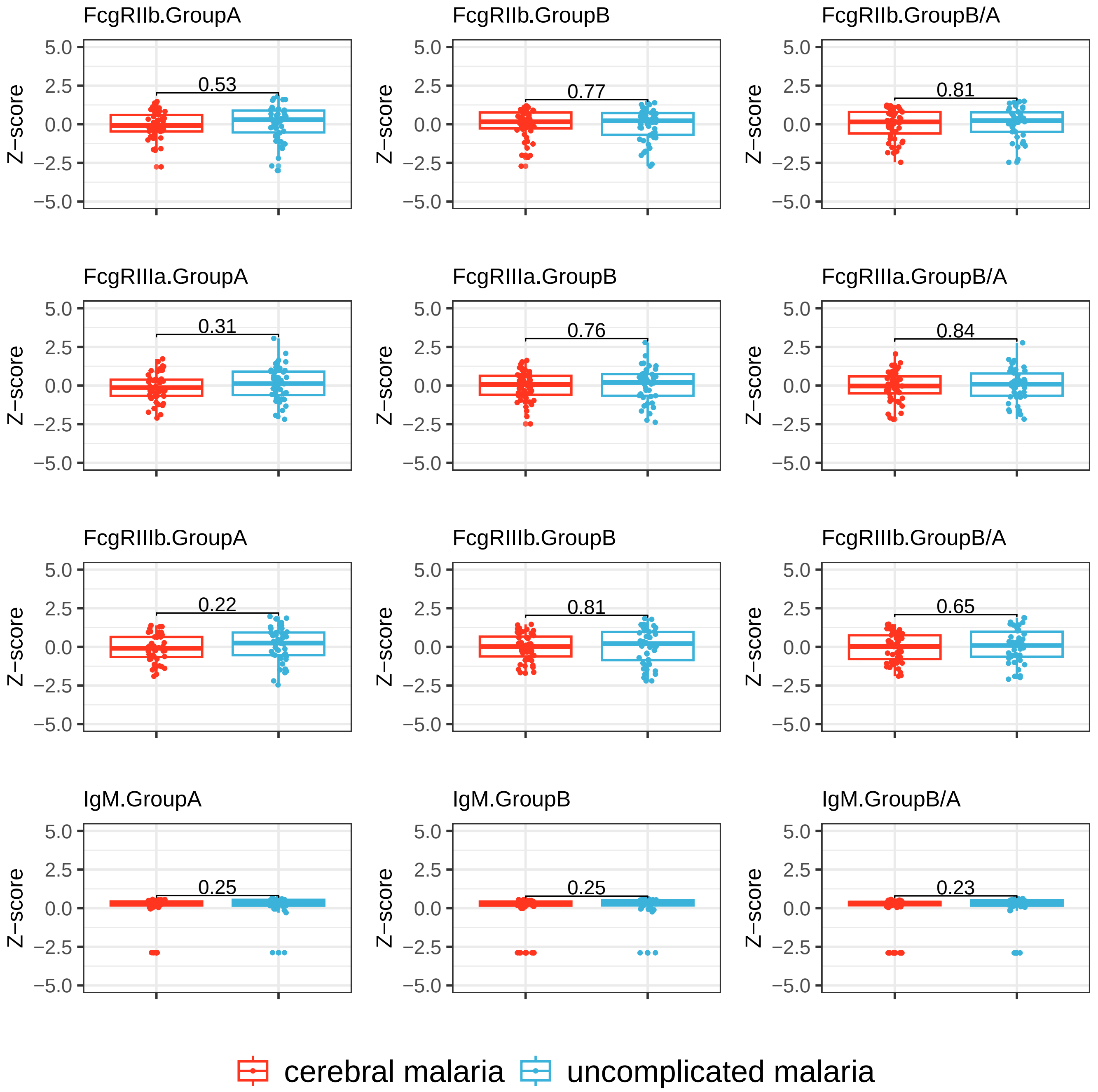

FcgRIIb.GroupA
FcgRIIb.GroupB
FcgRIIb.GroupB/A
0.53
0.77
0.81
FcgRIIIa.GroupA
FcgRIIIa.GroupB
FcgRIIIa.GroupB/A
0.31
0.76
0.84
FcgRIIIb.GroupA
FcgRIIIb.GroupB
FcgRIIIb.GroupB/A
0.22
0.81
0.65
IgM.GroupA
IgM.GroupB
IgM.GroupB/A
0.25
0.25
0.23
Z−score
5.0
2.5
0.0
−2.5
−5.0
cerebral malaria
uncomplicated malaria

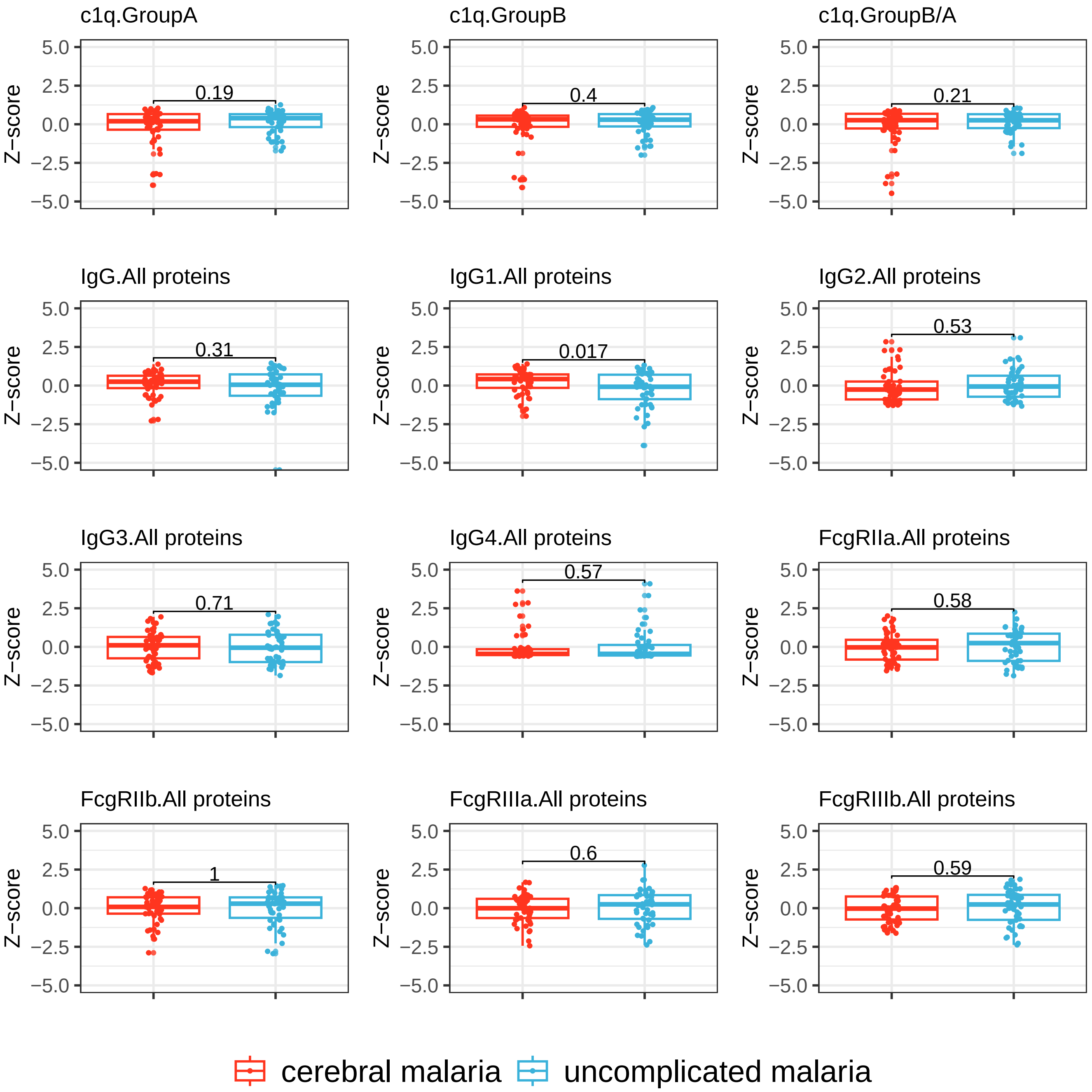

c1q.GroupA
0.19
c1q.GroupB
0.4
c1q.GroupB/A
0.21
IgG.All proteins
0.31
IgG1.All proteins
0.017
IgG2.All proteins
0.53
IgG3.All proteins
0.71
IgG4.All proteins
0.57
FcgRIIa.All proteins
0.58
FcgRIIb.All proteins
1
FcgRIIIa.All proteins
0.6
FcgRIIIb.All proteins
0.59
Z−score
5.0
2.5
0.0
−2.5
−5.0
cerebral malaria
uncomplicated malaria

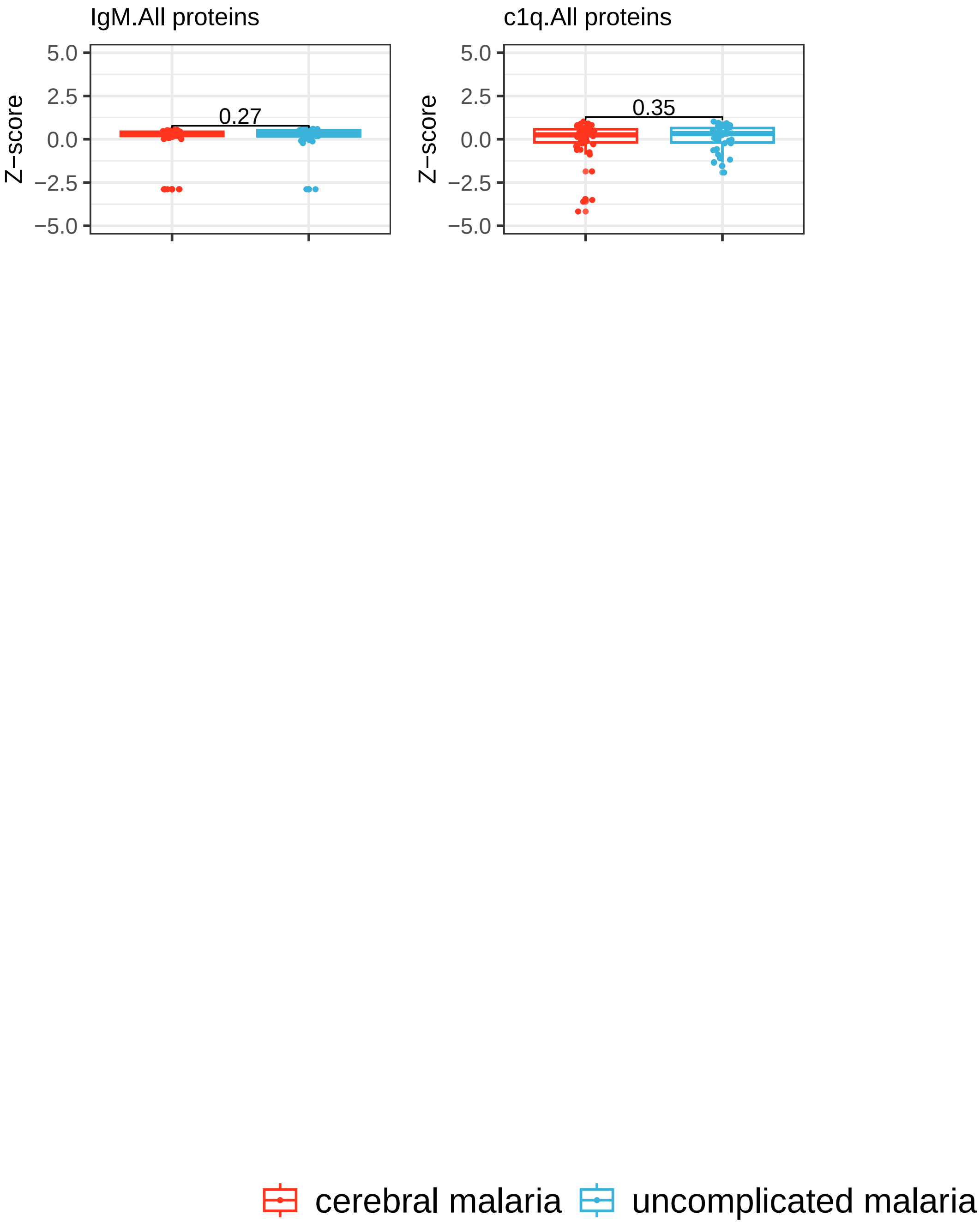

IgM.All proteins
c1q.All proteins
Z−score
5.0
2.5
0.0
−2.5
−5.0
0.27
0.35
cerebral malaria
uncomplicated malaria
