# Supplementary material for: A systems serology approach to identifying key antibody correlates of protection from cerebral malaria in Malawian children
Source: BMC Med. 2024 Sep 12;22:388. doi: 10.1186/s12916-024-03604-8 (PMC11396342; doi:10.1186/s12916-024-03604-8)
Supplement: Supplementary file 3 — Additional file 3: Box and whisker plots comparing cerebral and uncomplicated malaria for all variables. [file 12916_2024_3604_MOESM3_ESM.pdf]

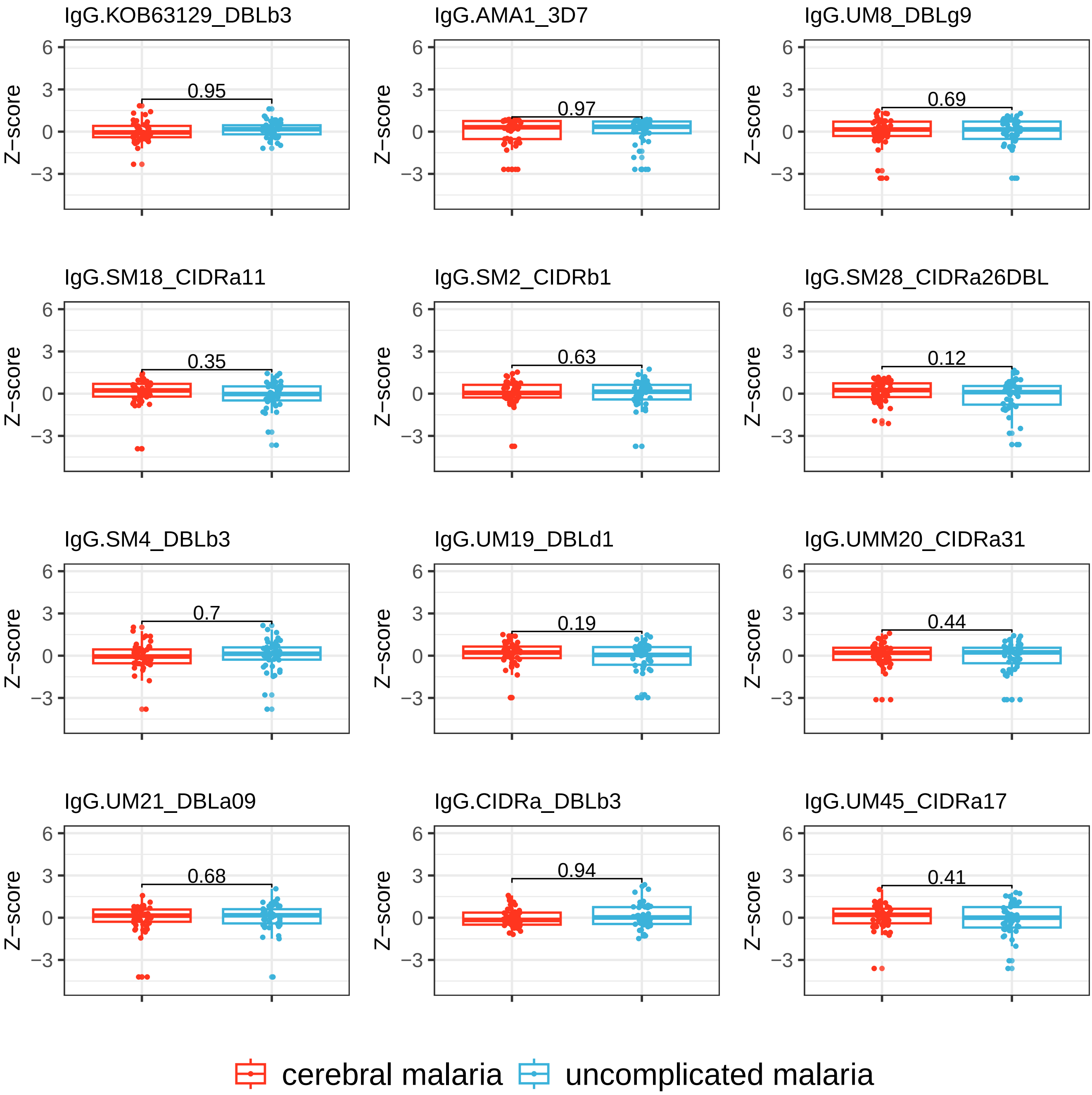
IgG.KOB63129_DBLb3
0.95
IgG.AMA1_3D7
0.97
IgG.UM8_DBLg9
0.69
IgG.SM18_CIDRa11
0.35
IgG.SM2_CIDRb1
0.63
IgG.SM28_CIDRa26DBL
0.12
IgG.SM4_DBLb3
0.7
IgG.UM19_DBLd1
0.19
IgG.UMM20_CIDRa31
0.44
IgG.UM21_DBLa09
0.68
IgG.CIDRa_DBLb3
0.94
IgG.UM45_CIDRa17
0.41
Z−score
6
3
0
−3
cerebral malaria
uncomplicated malaria

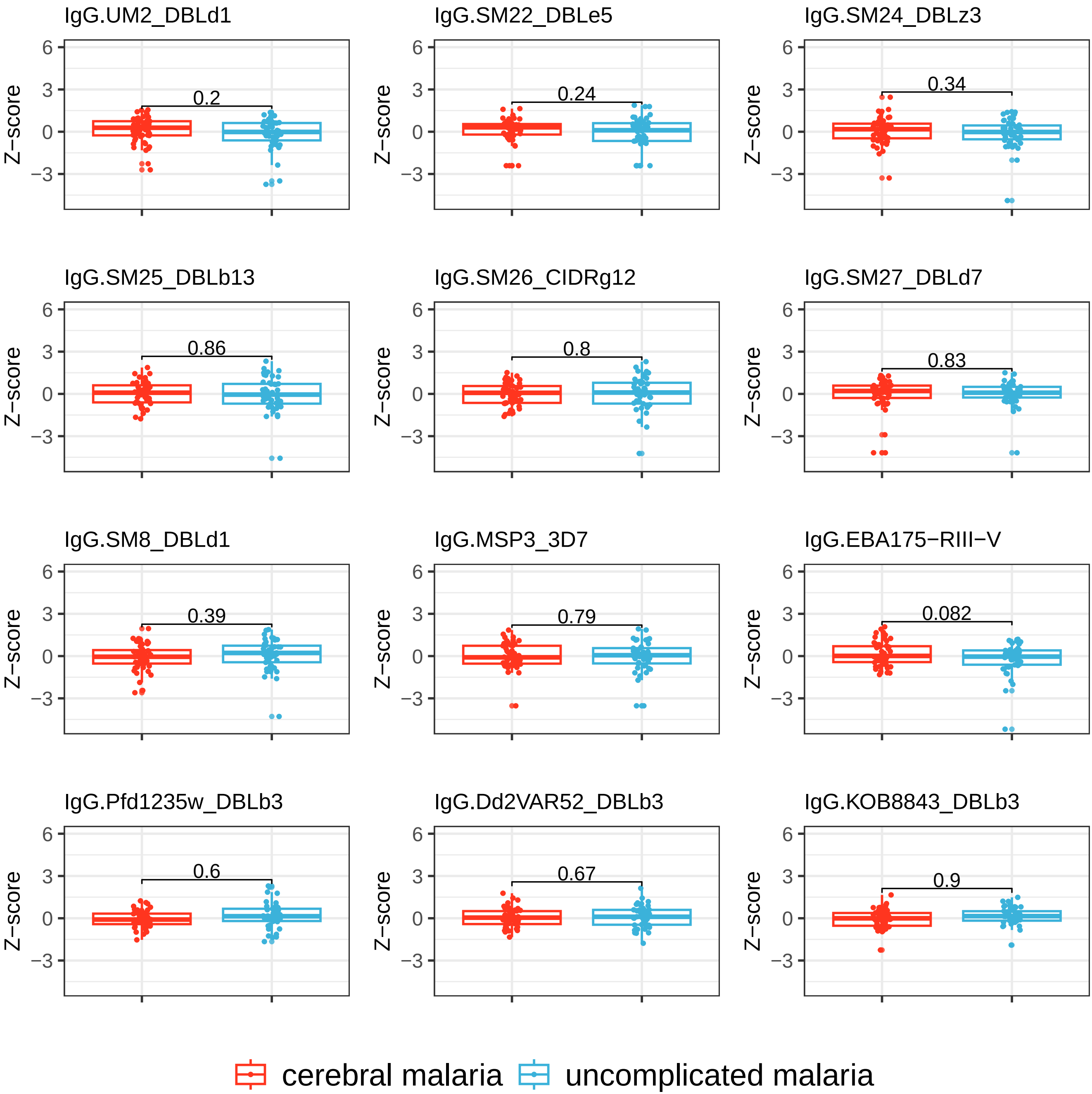
IgG.UM2_DBLd1
0.2
IgG.SM22_DBLe5
0.24
IgG.SM24_DBLz3
0.34
IgG.SM25_DBLb13
0.86
IgG.SM26_CIDRg12
0.8
IgG.SM27_DBLd7
0.83
IgG.SM8_DBLd1
0.39
IgG.MSP3_3D7
0.79
IgG.EBA175−RIII−V
0.082
IgG.Pfd1235w_DBLb3
0.6
IgG.Dd2VAR52_DBLb3
0.67
IgG.KOB8843_DBLb3
0.9
Z−score
6
3
0
−3
cerebral malaria
uncomplicated malaria

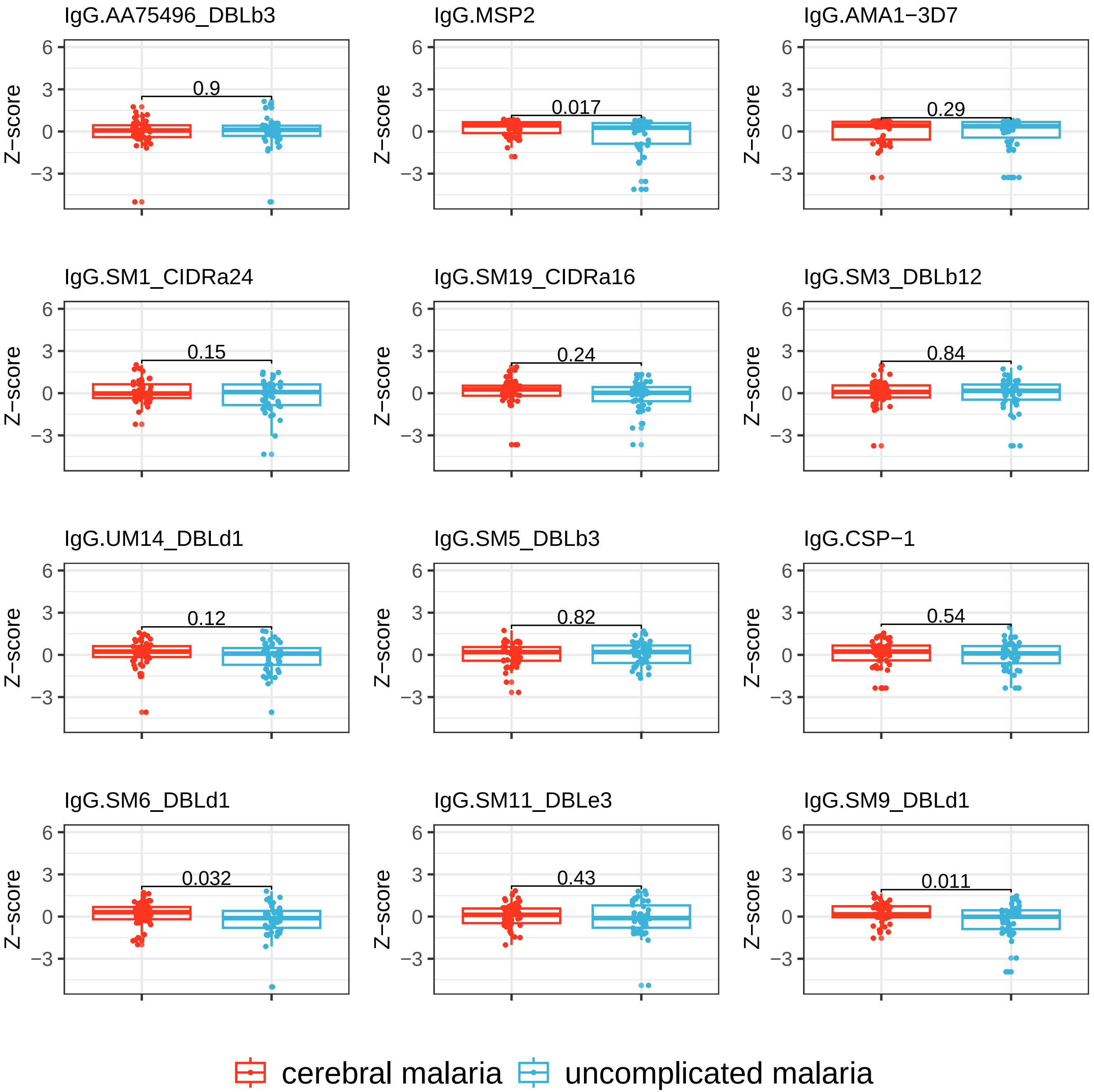
IgG.AA75496_DBLb3
0.9
IgG.MSP2
0.017
IgG.AMA1−3D7
0.29
IgG.SM1_CIDRa24
0.15
IgG.SM19_CIDRa16
0.24
IgG.SM3_DBLb12
0.84
IgG.UM14_DBLd1
0.12
IgG.SM5_DBLb3
0.82
IgG.CSP−1
0.54
IgG.SM6_DBLd1
0.032
IgG.SM11_DBLe3
0.43
IgG.SM9_DBLd1
0.011
Z−score
6
3
0
−3
cerebral malaria
uncomplicated malaria

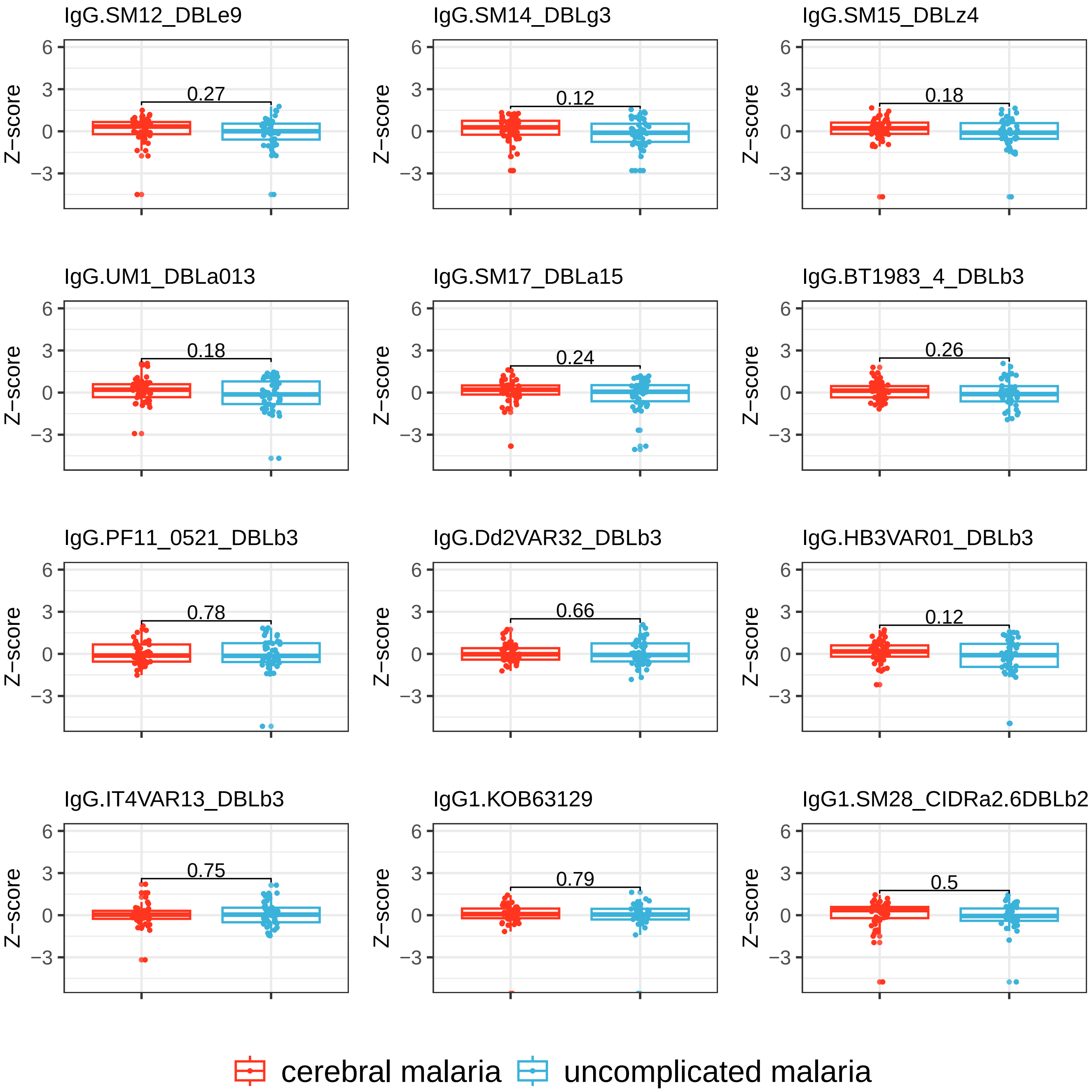
IgG.SM12_DBLe9
0.27
IgG.SM14_DBLg3
0.12
IgG.SM15_DBLz4
0.18
IgG.UM1_DBLa013
0.18
IgG.SM17_DBLa15
0.24
IgG.BT1983_4_DBLb3
0.26
IgG.PF11_0521_DBLb3
0.78
IgG.Dd2VAR32_DBLb3
0.66
IgG.HB3VAR01_DBLb3
0.12
IgG.IT4VAR13_DBLb3
0.75
IgG1.KOB63129
0.79
IgG1.SM28_CIDRa2.6DBLb2
0.5
Z−score
6
3
0
−3
cerebral malaria
uncomplicated malaria

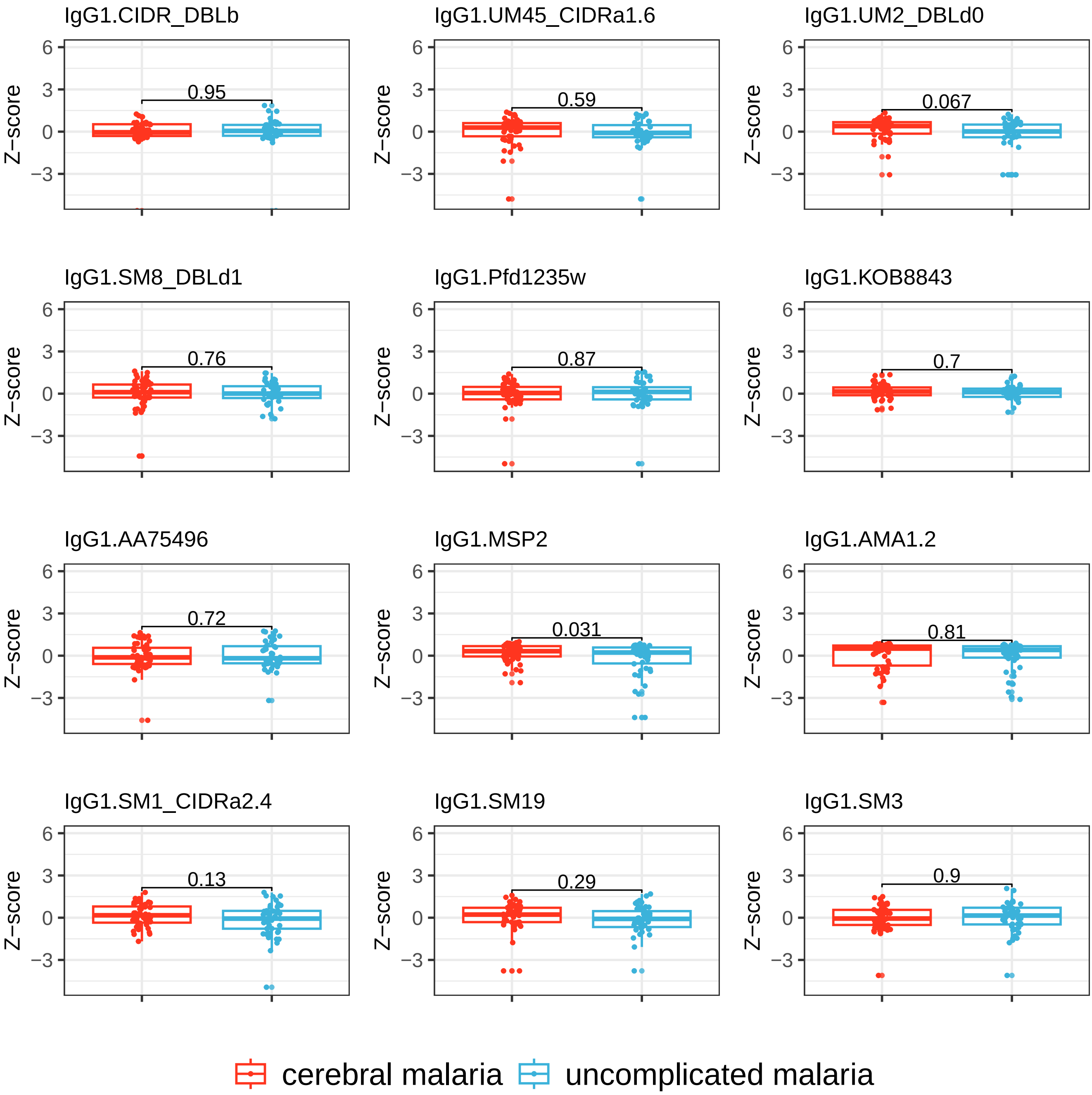
IgG1.CIDR_DBLb
0.95
IgG1.UM45_CIDRa1.6
0.59
IgG1.UM2_DBLd0
0.067
IgG1.SM8_DBLd1
0.76
IgG1.Pfd1235w
0.87
IgG1.KOB8843
0.7
IgG1.AA75496
0.72
IgG1.MSP2
0.031
IgG1.AMA1.2
0.81
IgG1.SM1_CIDRa2.4
0.13
IgG1.SM19
0.29
IgG1.SM3
0.9
Z−score
6
3
0
−3
cerebral malaria
uncomplicated malaria

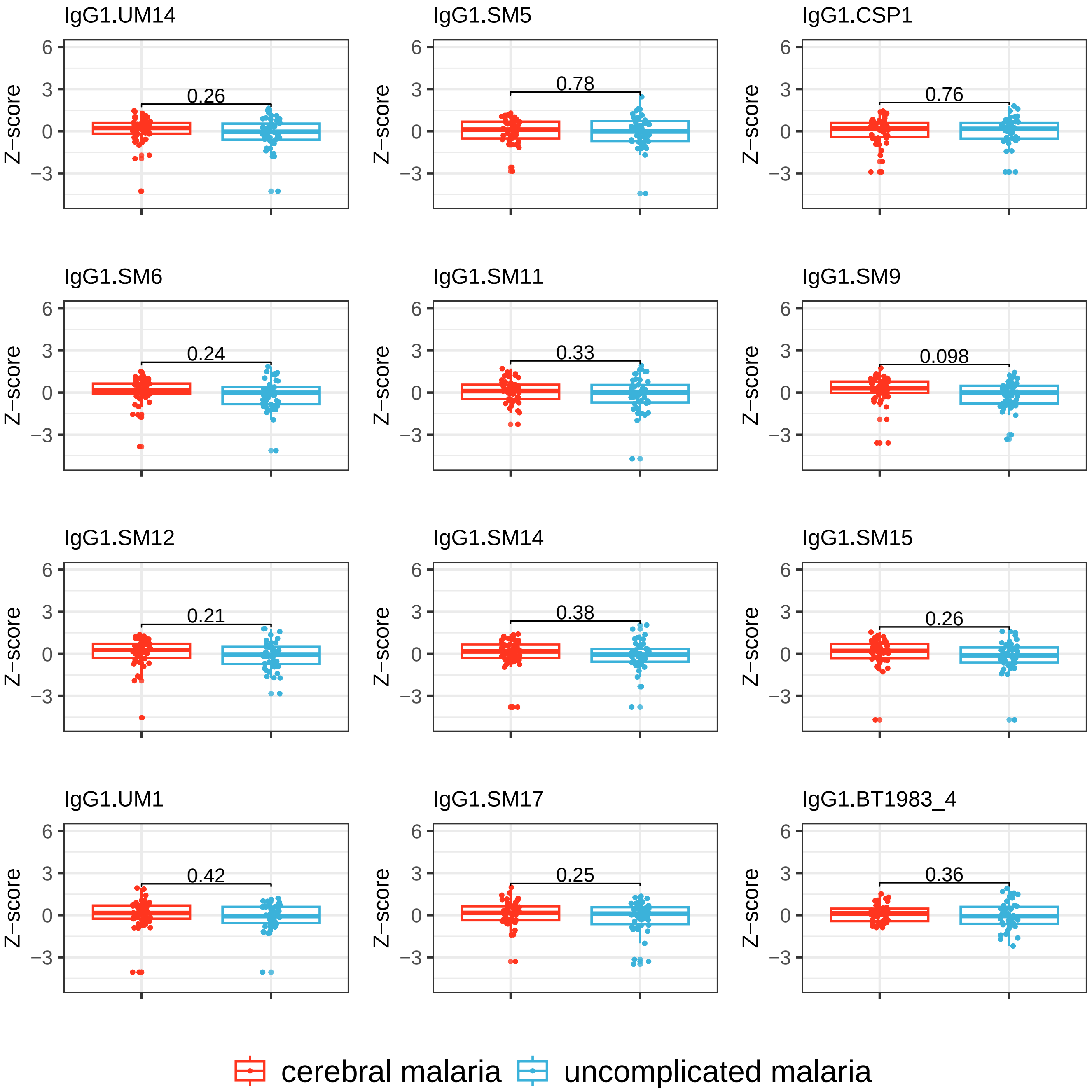
IgG1.UM14
0.26
IgG1.SM5
0.78
IgG1.CSP1
0.76
IgG1.SM6
0.24
IgG1.SM11
0.33
IgG1.SM9
0.098
IgG1.SM12
0.21
IgG1.SM14
0.38
IgG1.SM15
0.26
IgG1.UM1
0.42
IgG1.SM17
0.25
IgG1.BT1983_4
0.36
Z−score
6
3
0
−3
cerebral malaria
uncomplicated malaria

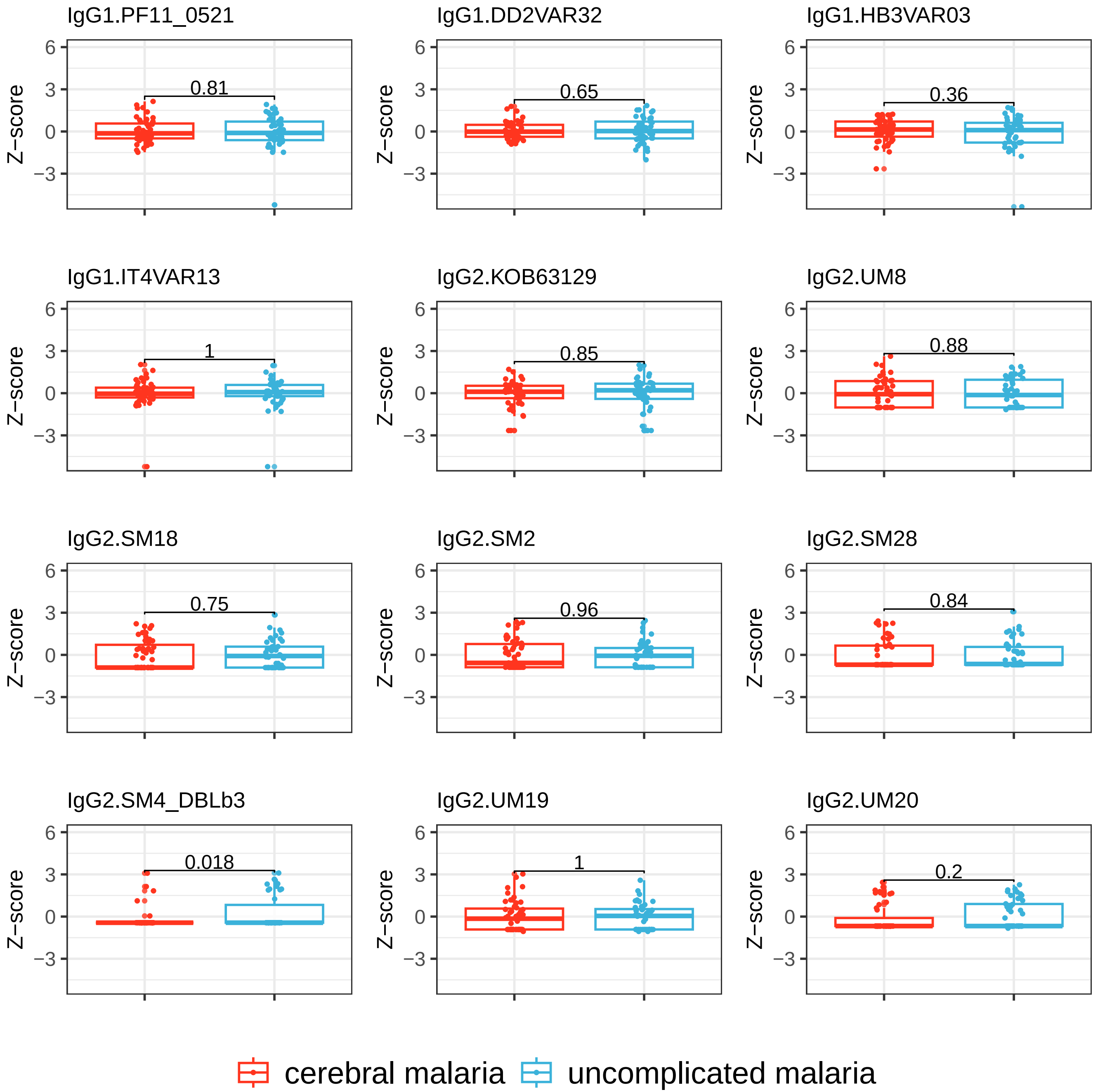
IgG1.PF11_0521
0.81
IgG1.DD2VAR32
0.65
IgG1.HB3VAR03
0.36
IgG1.IT4VAR13
1
IgG2.KOB63129
0.85
IgG2.UM8
0.88
IgG2.SM18
0.75
IgG2.SM2
0.96
IgG2.SM28
0.84
IgG2.SM4_DBLb3
0.018
IgG2.UM19
1
IgG2.UM20
0.2
Z−score
6
3
0
−3
cerebral malaria
uncomplicated malaria

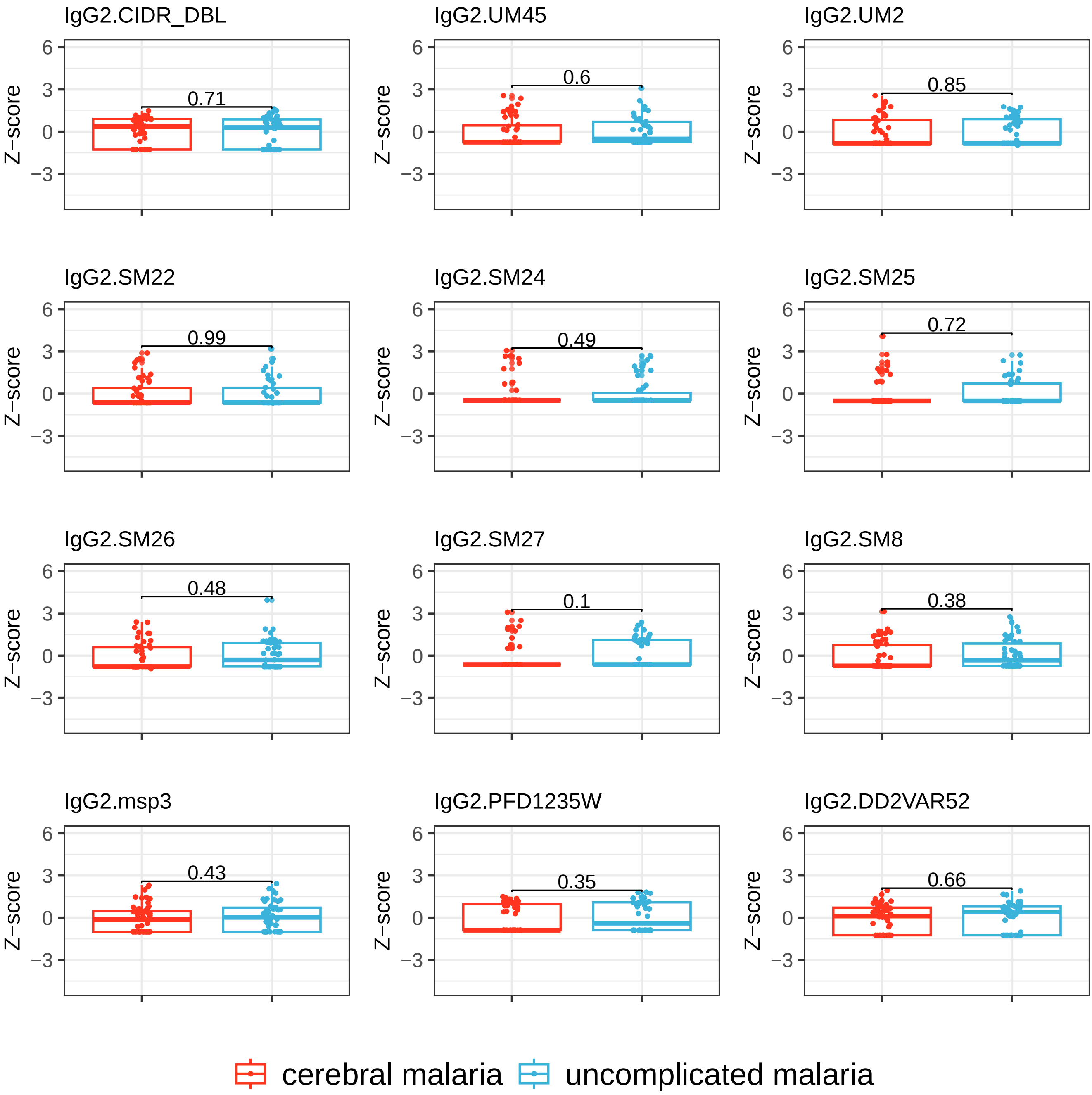

IgG2.CIDR_DBL
0.71
IgG2.UM45
0.6
IgG2.UM2
0.85
IgG2.SM22
0.99
IgG2.SM24
0.49
IgG2.SM25
0.72
IgG2.SM26
0.48
IgG2.SM27
0.1
IgG2.SM8
0.38
IgG2.msp3
0.43
IgG2.PFD1235W
0.35
IgG2.DD2VAR52
0.66
Z−score
6
3
0
−3
cerebral malaria
uncomplicated malaria

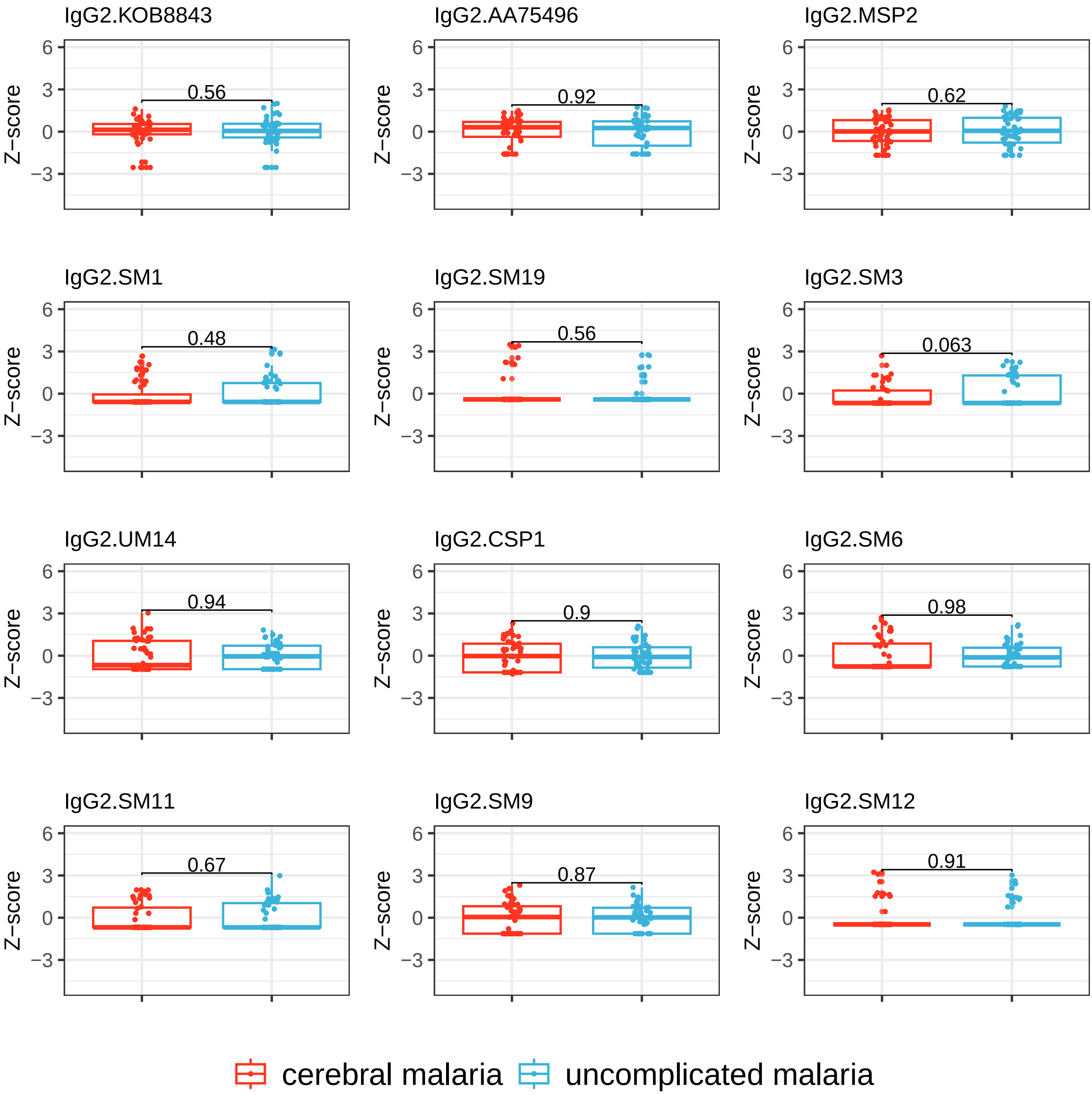
IgG2.KOB8843
0.56
IgG2.AA75496
0.92
IgG2.MSP2
0.62
IgG2.SM1
0.48
IgG2.SM19
0.56
IgG2.SM3
0.063
IgG2.UM14
0.94
IgG2.CSP1
0.9
IgG2.SM6
0.98
IgG2.SM11
0.67
IgG2.SM9
0.87
IgG2.SM12
0.91
Z−score
6
3
0
−3
cerebral malaria
uncomplicated malaria

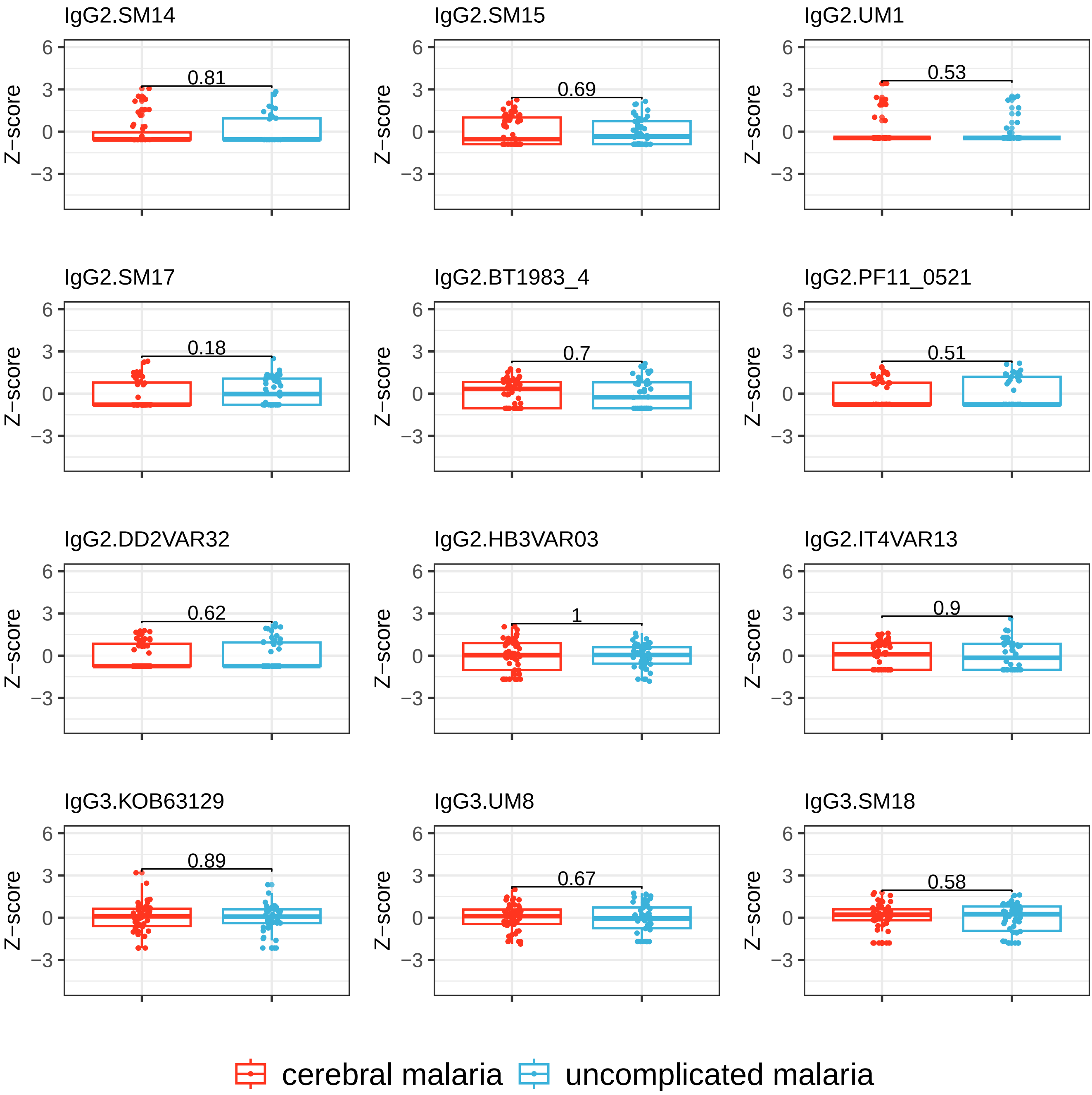
IgG2.SM14
0.81
IgG2.SM15
0.69
IgG2.UM1
0.53
IgG2.SM17
0.18
IgG2.BT1983_4
0.7
IgG2.PF11_0521
0.51
IgG2.DD2VAR32
0.62
IgG2.HB3VAR03
1
IgG2.IT4VAR13
0.9
IgG3.KOB63129
0.89
IgG3.UM8
0.67
IgG3.SM18
0.58
Z−score
6
3
0
−3
cerebral malaria
uncomplicated malaria

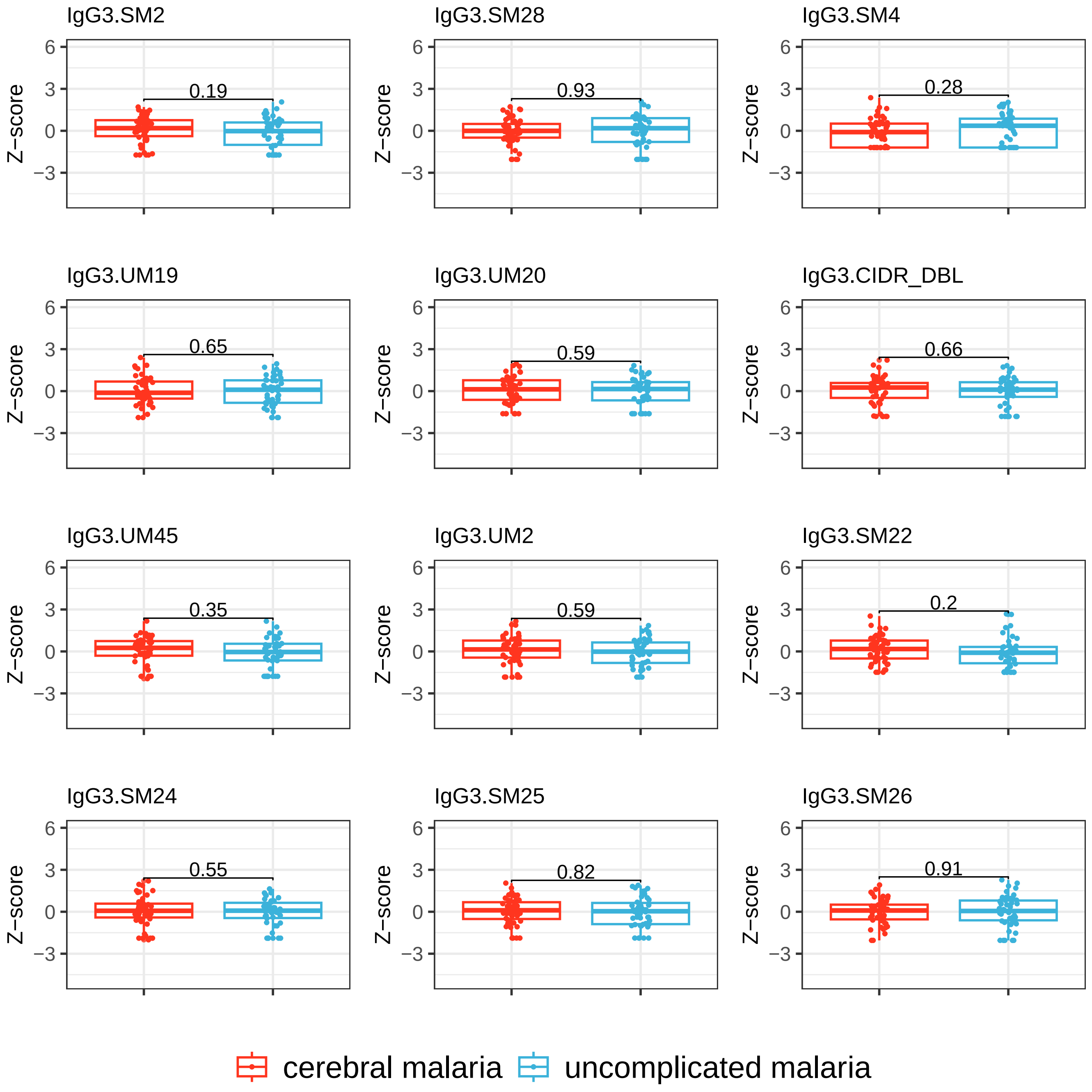
IgG3.SM2
0.19
IgG3.SM28
0.93
IgG3.SM4
0.28
IgG3.UM19
0.65
IgG3.UM20
0.59
IgG3.CIDR_DBL
0.66
IgG3.UM45
0.35
IgG3.UM2
0.59
IgG3.SM22
0.2
IgG3.SM24
0.55
IgG3.SM25
0.82
IgG3.SM26
0.91
Z−score
6
3
0
−3
cerebral malaria
uncomplicated malaria

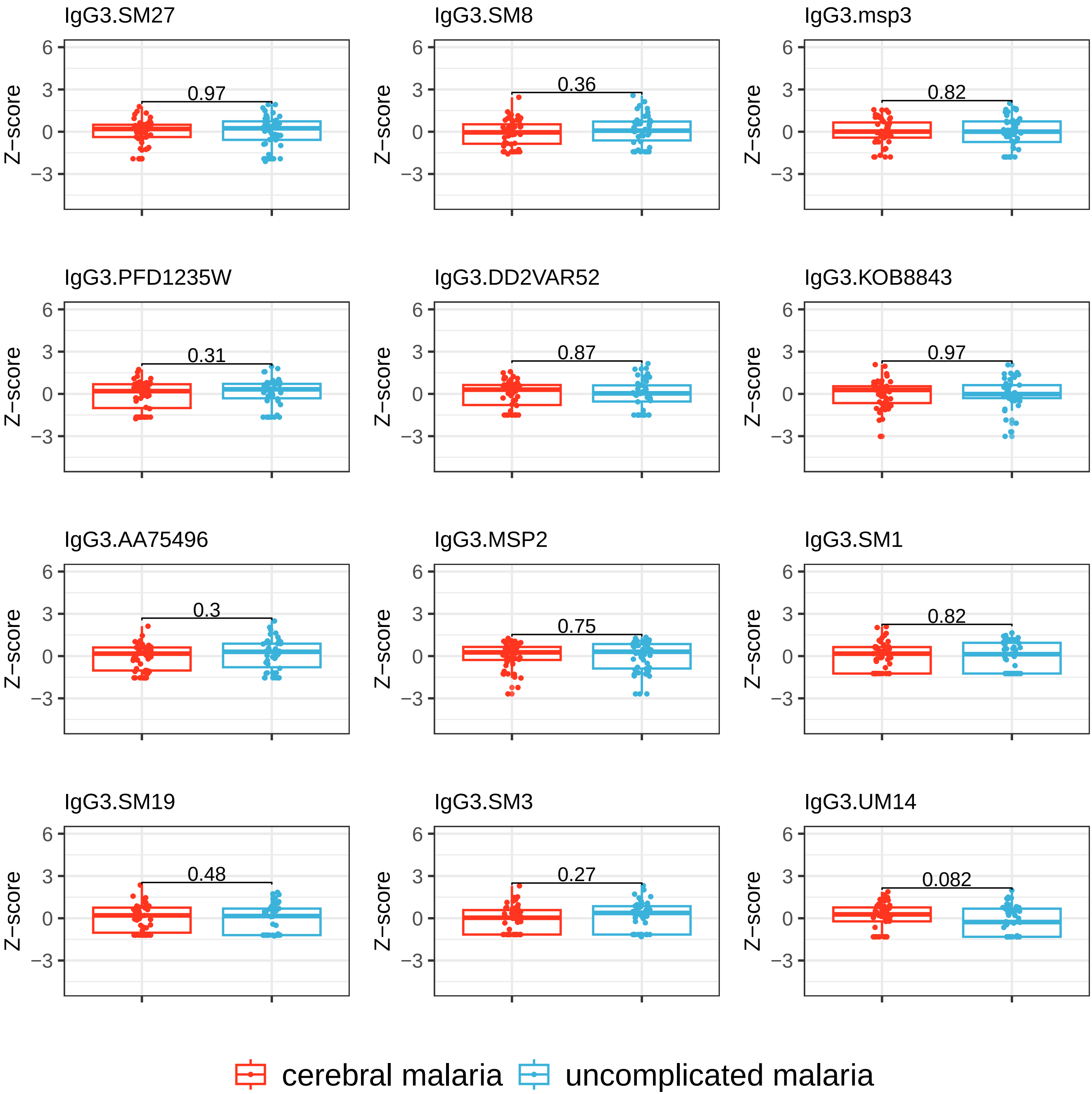
IgG3.SM27
0.97
IgG3.SM8
0.36
IgG3.msp3
0.82
IgG3.PFD1235W
0.31
IgG3.DD2VAR52
0.87
IgG3.KOB8843
0.97
IgG3.AA75496
0.3
IgG3.MSP2
0.75
IgG3.SM1
0.82
IgG3.SM19
0.48
IgG3.SM3
0.27
IgG3.UM14
0.082
Z−score
6
3
0
−3
cerebral malaria
uncomplicated malaria

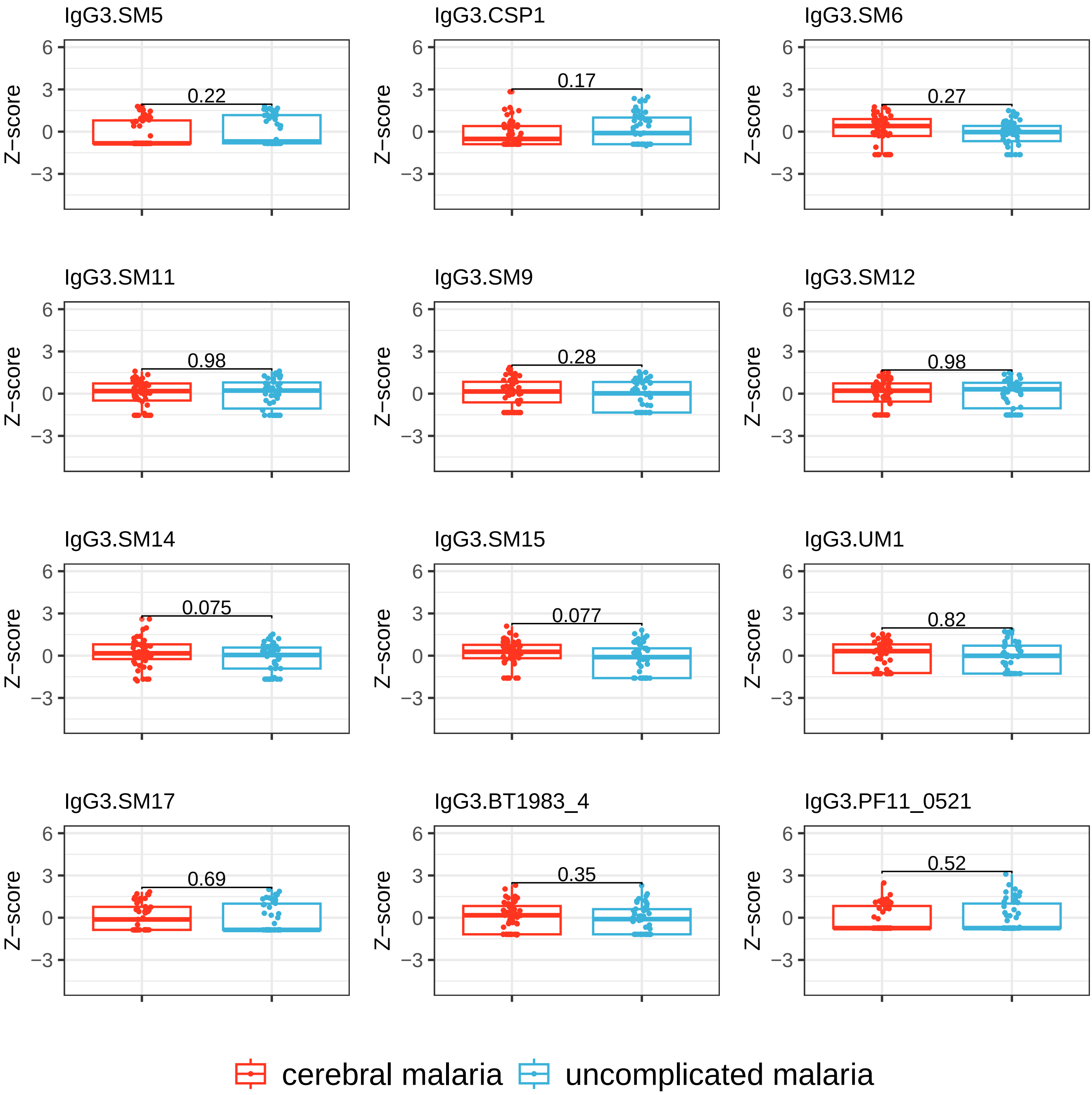
IgG3.SM5
0.22
IgG3.CSP1
0.17
IgG3.SM6
0.27
IgG3.SM11
0.98
IgG3.SM9
0.28
IgG3.SM12
0.98
IgG3.SM14
0.075
IgG3.SM15
0.077
IgG3.UM1
0.82
IgG3.SM17
0.69
IgG3.BT1983_4
0.35
IgG3.PF11_0521
0.52
Z−score
6
3
0
−3
cerebral malaria
uncomplicated malaria

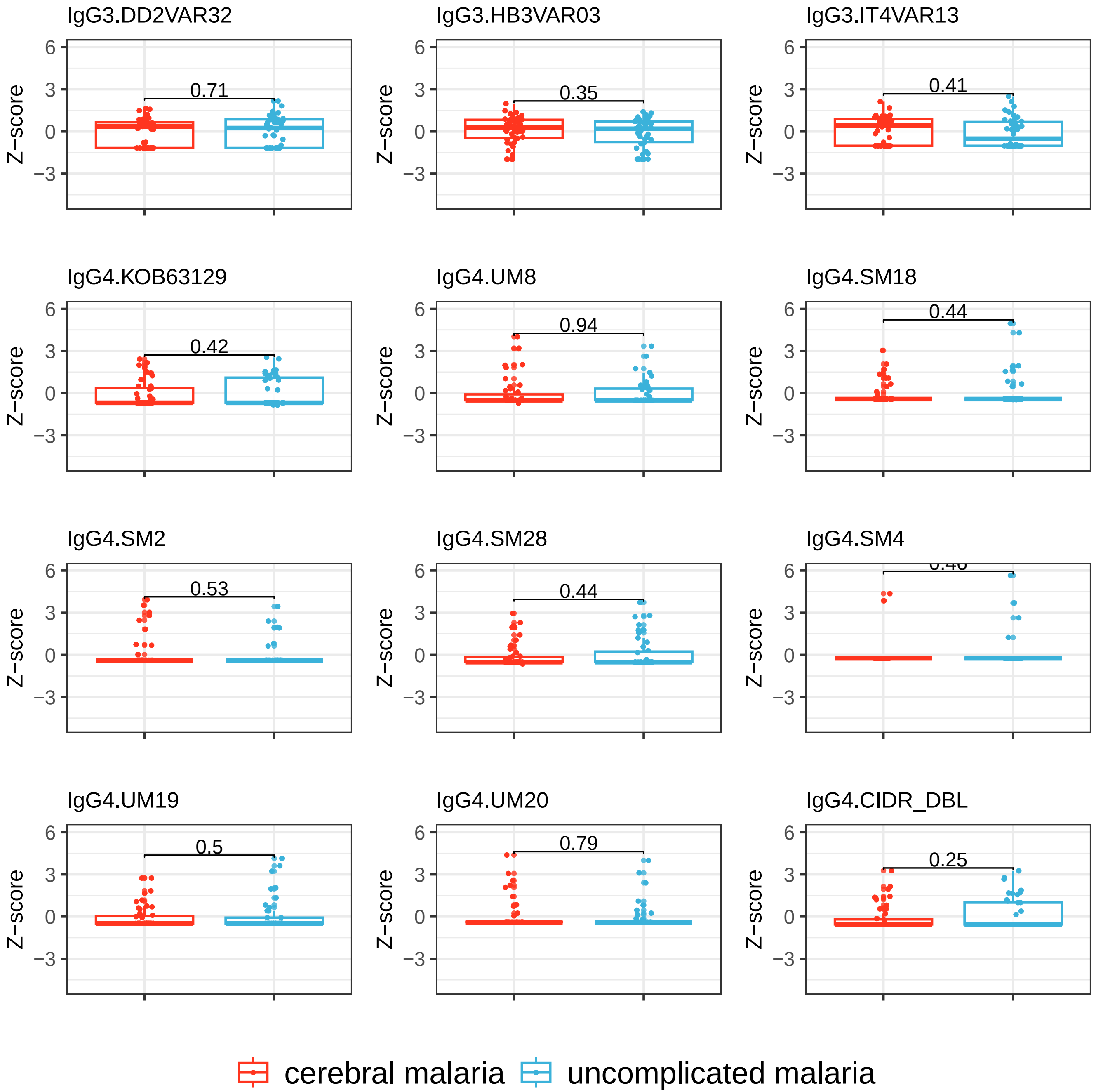

IgG3.DD2VAR32
0.71
IgG3.HB3VAR03
0.35
IgG3.IT4VAR13
0.41
IgG4.KOB63129
0.42
IgG4.UM8
0.94
IgG4.SM18
0.44
IgG4.SM2
0.53
IgG4.SM28
0.44
IgG4.SM4
IgG4.UM19
0.5
IgG4.UM20
0.79
IgG4.CIDR_DBL
0.25
Z−score
6
3
0
−3
cerebral malaria
uncomplicated malaria

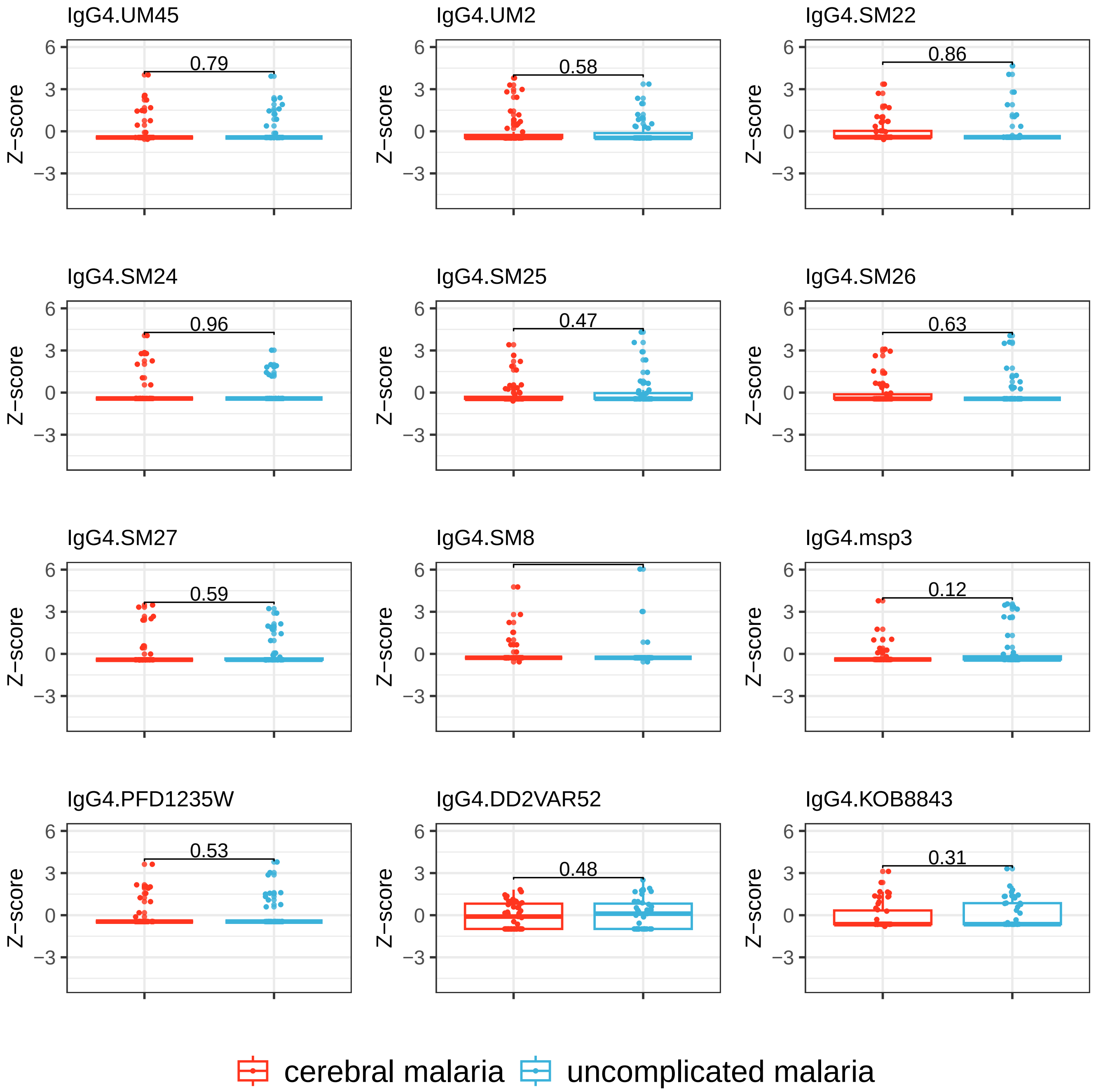
IgG4.UM45
0.79
IgG4.UM2
0.58
IgG4.SM22
0.86
IgG4.SM24
0.96
IgG4.SM25
0.47
IgG4.SM26
0.63
IgG4.SM27
0.59
IgG4.SM8
IgG4.msp3
0.12
IgG4.PFD1235W
0.53
IgG4.DD2VAR52
0.48
IgG4.KOB8843
0.31
Z−score
6
3
0
−3
cerebral malaria
uncomplicated malaria

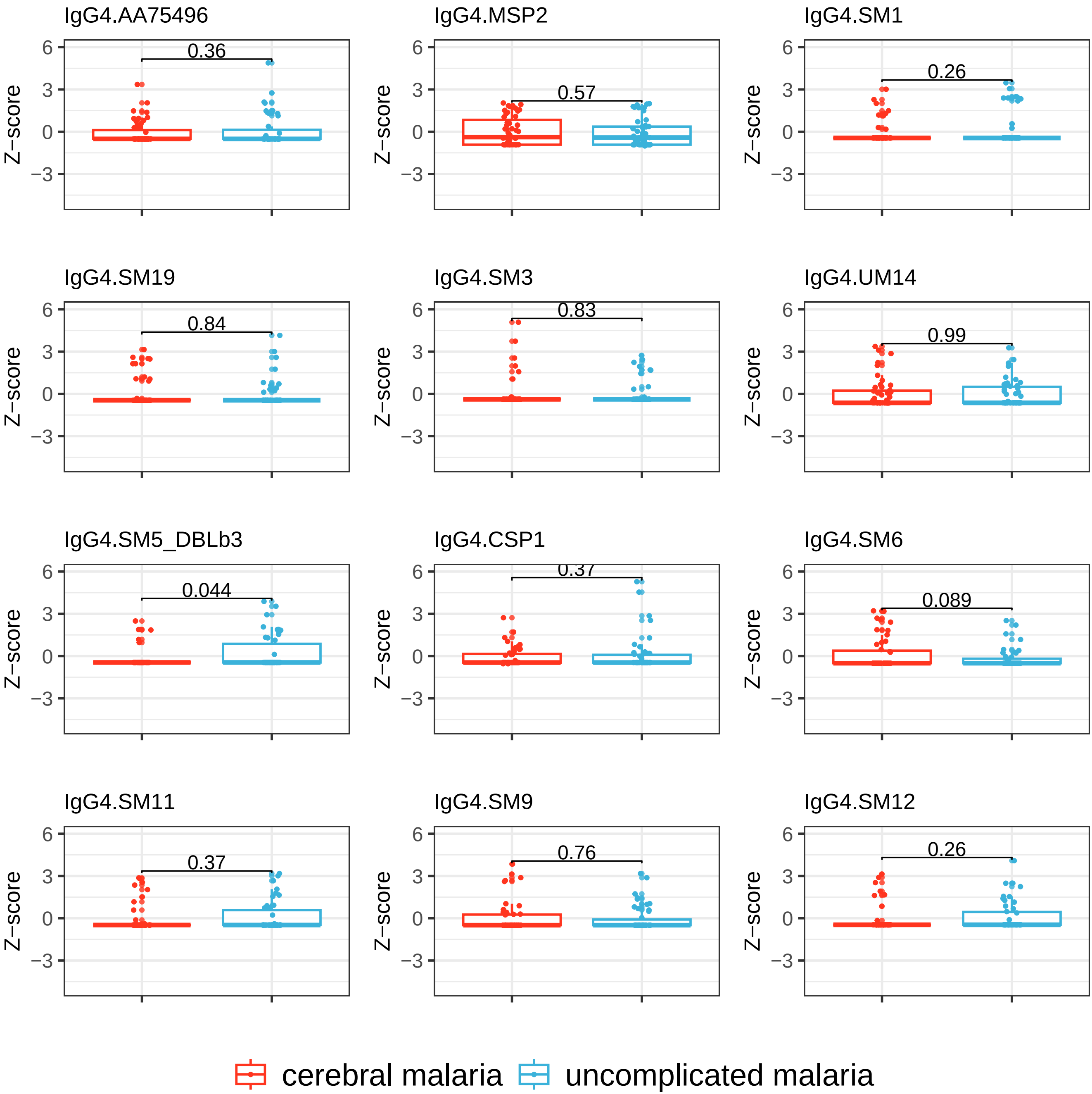
IgG4.AA75496
0.36
IgG4.MSP2
0.57
IgG4.SM1
0.26
IgG4.SM19
0.84
IgG4.SM3
0.83
IgG4.UM14
0.99
IgG4.SM5_DBLb3
0.044
IgG4.CSP1
0.37
IgG4.SM6
0.089
IgG4.SM11
0.37
IgG4.SM9
0.76
IgG4.SM12
0.26
Z−score
6
3
0
−3
cerebral malaria
uncomplicated malaria

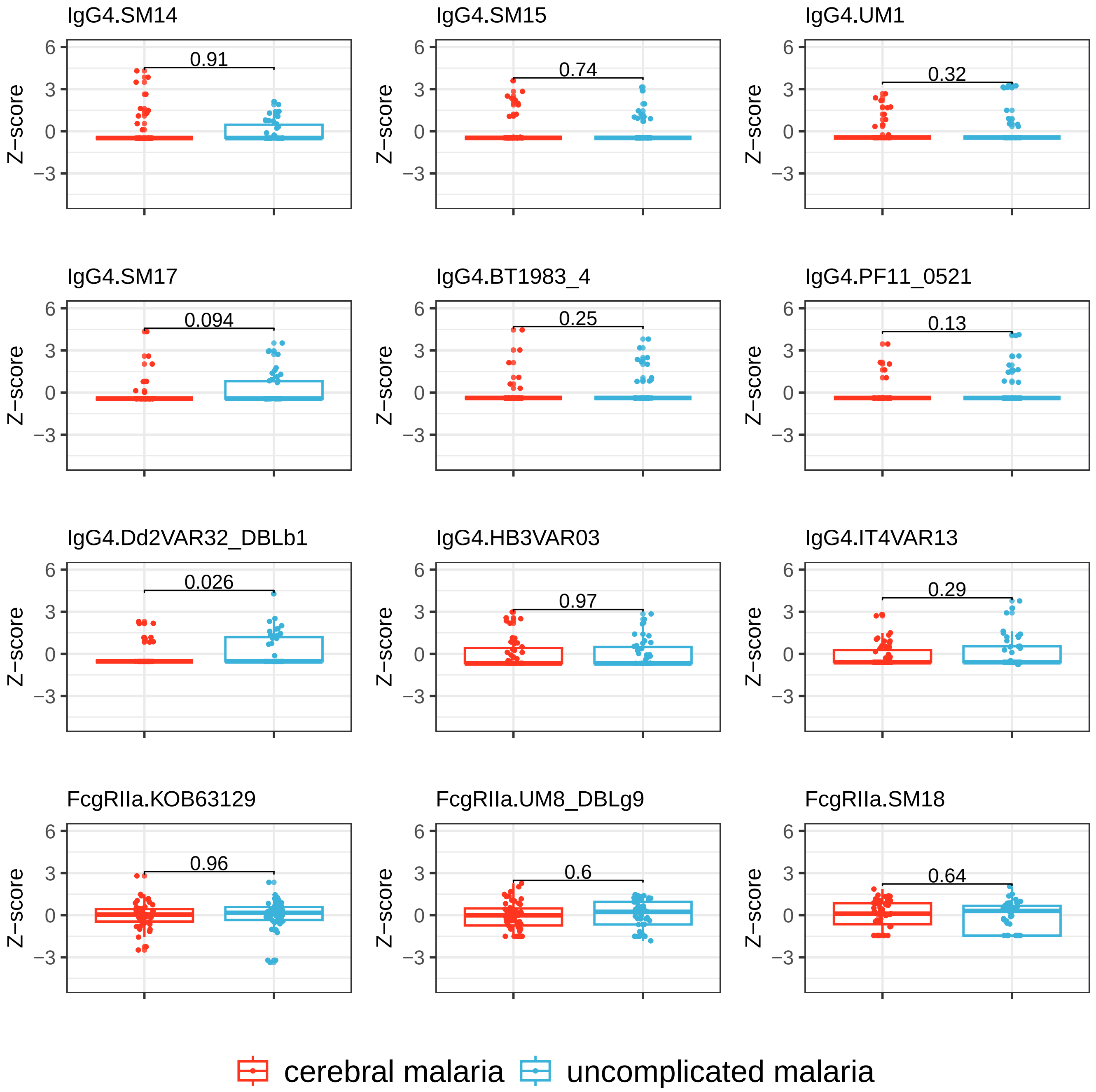

IgG4.SM14
0.91
IgG4.SM15
0.74
IgG4.UM1
0.32
IgG4.SM17
0.094
IgG4.BT1983_4
0.25
IgG4.PF11_0521
0.13
IgG4.Dd2VAR32_DBLb1
0.026
IgG4.HB3VAR03
0.97
IgG4.IT4VAR13
0.29
FcgRIIa.KOB63129
0.96
FcgRIIa.UM8_DBLg9
0.6
FcgRIIa.SM18
0.64
Z−score
6
3
0
−3
cerebral malaria
uncomplicated malaria

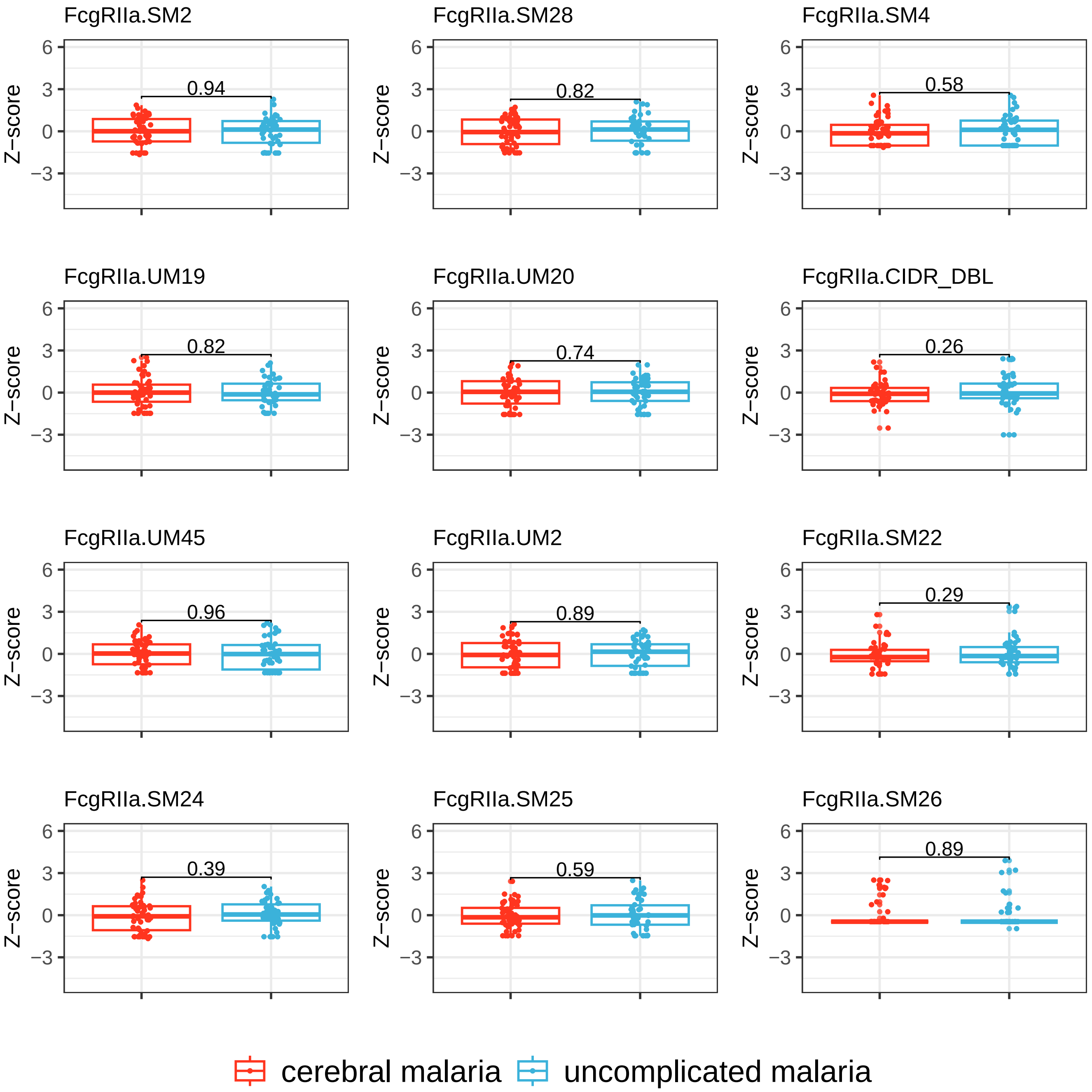

FcgRIIa.SM2
0.94
FcgRIIa.SM28
0.82
FcgRIIa.SM4
0.58
FcgRIIa.UM19
0.82
FcgRIIa.UM20
0.74
FcgRIIa.CIDR_DBL
0.26
FcgRIIa.UM45
0.96
FcgRIIa.UM2
0.89
FcgRIIa.SM22
0.29
FcgRIIa.SM24
0.39
FcgRIIa.SM25
0.59
FcgRIIa.SM26
0.89
Z−score
6
3
0
−3
cerebral malaria
uncomplicated malaria

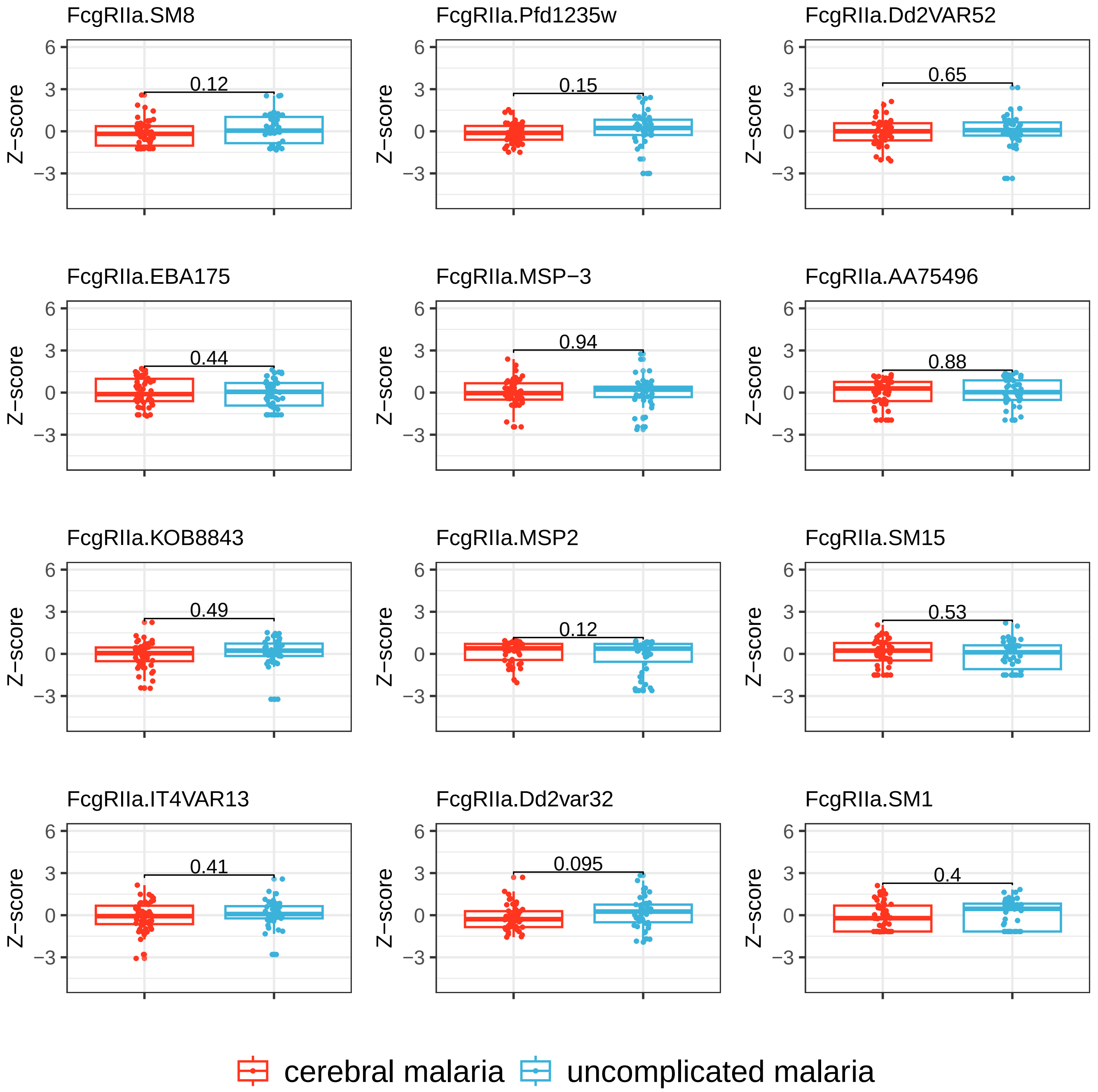
FcgRIIa.SM8
0.12
FcgRIIa.Pfd1235w
0.15
FcgRIIa.Dd2VAR52
0.65
FcgRIIa.EBA175
0.44
FcgRIIa.MSP−3
0.94
FcgRIIa.AA75496
0.88
FcgRIIa.KOB8843
0.49
FcgRIIa.MSP2
0.12
FcgRIIa.SM15
0.53
FcgRIIa.IT4VAR13
0.41
FcgRIIa.Dd2var32
0.095
FcgRIIa.SM1
0.4
Z−score
6
3
0
−3
cerebral malaria
uncomplicated malaria

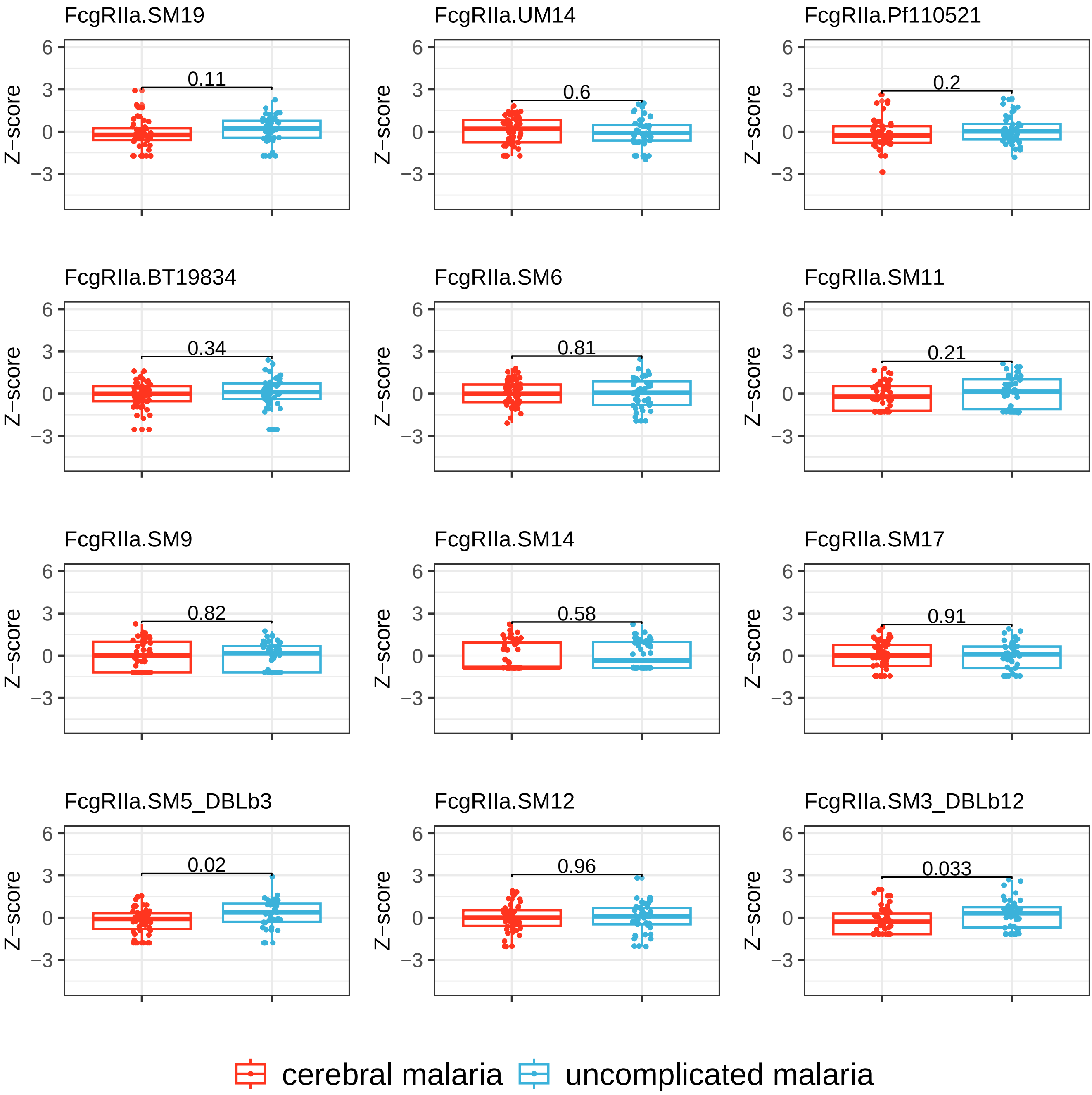
FcgRIIa.SM19
0.11
FcgRIIa.UM14
0.6
FcgRIIa.Pf110521
0.2
FcgRIIa.BT19834
0.34
FcgRIIa.SM6
0.81
FcgRIIa.SM11
0.21
FcgRIIa.SM9
0.82
FcgRIIa.SM14
0.58
FcgRIIa.SM17
0.91
FcgRIIa.SM5_DBLb3
0.02
FcgRIIa.SM12
0.96
FcgRIIa.SM3_DBLb12
0.033
Z−score
6
3
0
−3
cerebral malaria
uncomplicated malaria

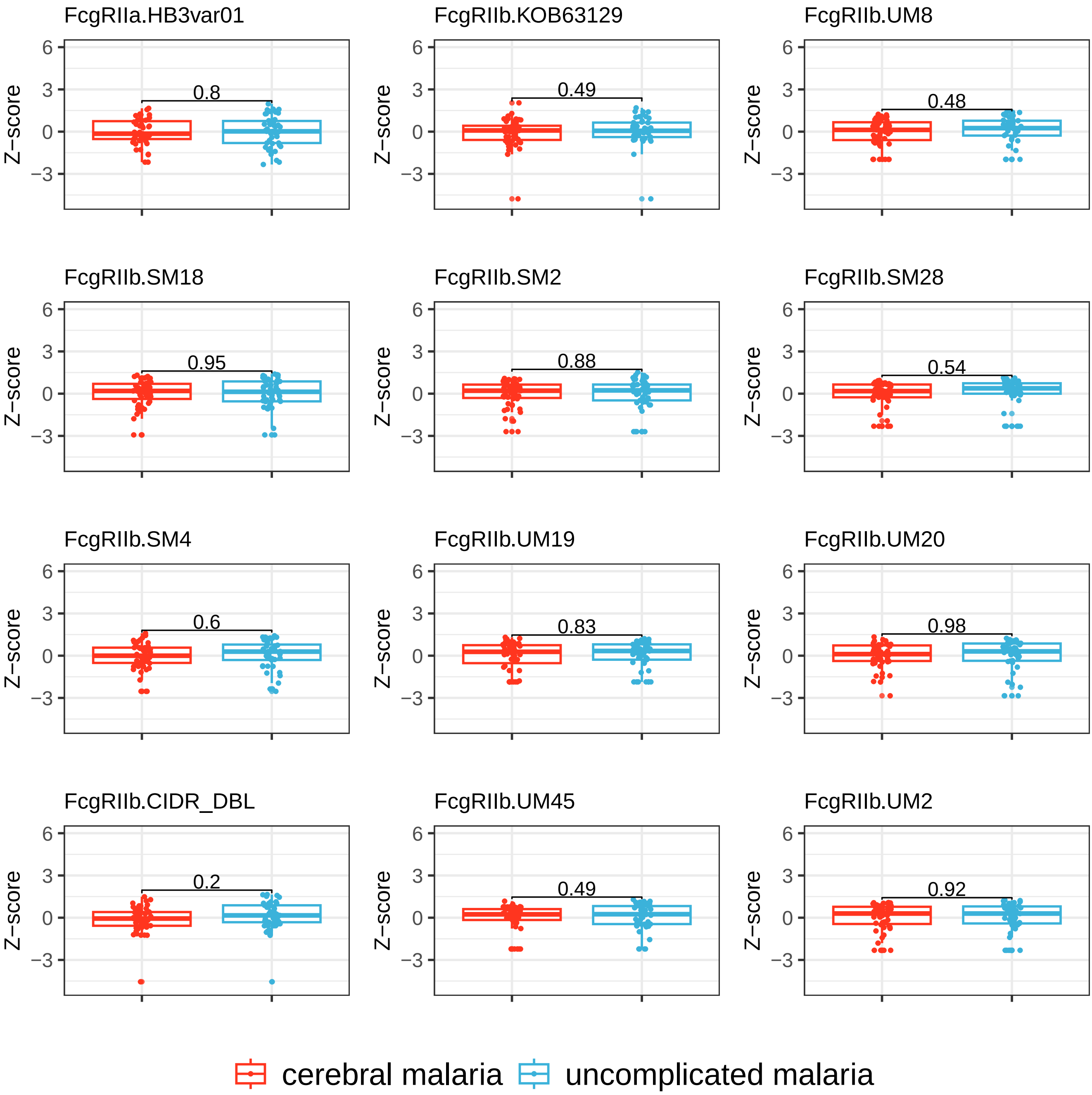
FcgRIIa.HB3var01
0.8
FcgRIIb.KOB63129
0.49
FcgRIIb.UM8
0.48
FcgRIIb.SM18
0.95
FcgRIIb.SM2
0.88
FcgRIIb.SM28
0.54
FcgRIIb.SM4
0.6
FcgRIIb.UM19
0.83
FcgRIIb.UM20
0.98
FcgRIIb.CIDR_DBL
0.2
FcgRIIb.UM45
0.49
FcgRIIb.UM2
0.92
Z−score
6
3
0
−3
cerebral malaria
uncomplicated malaria

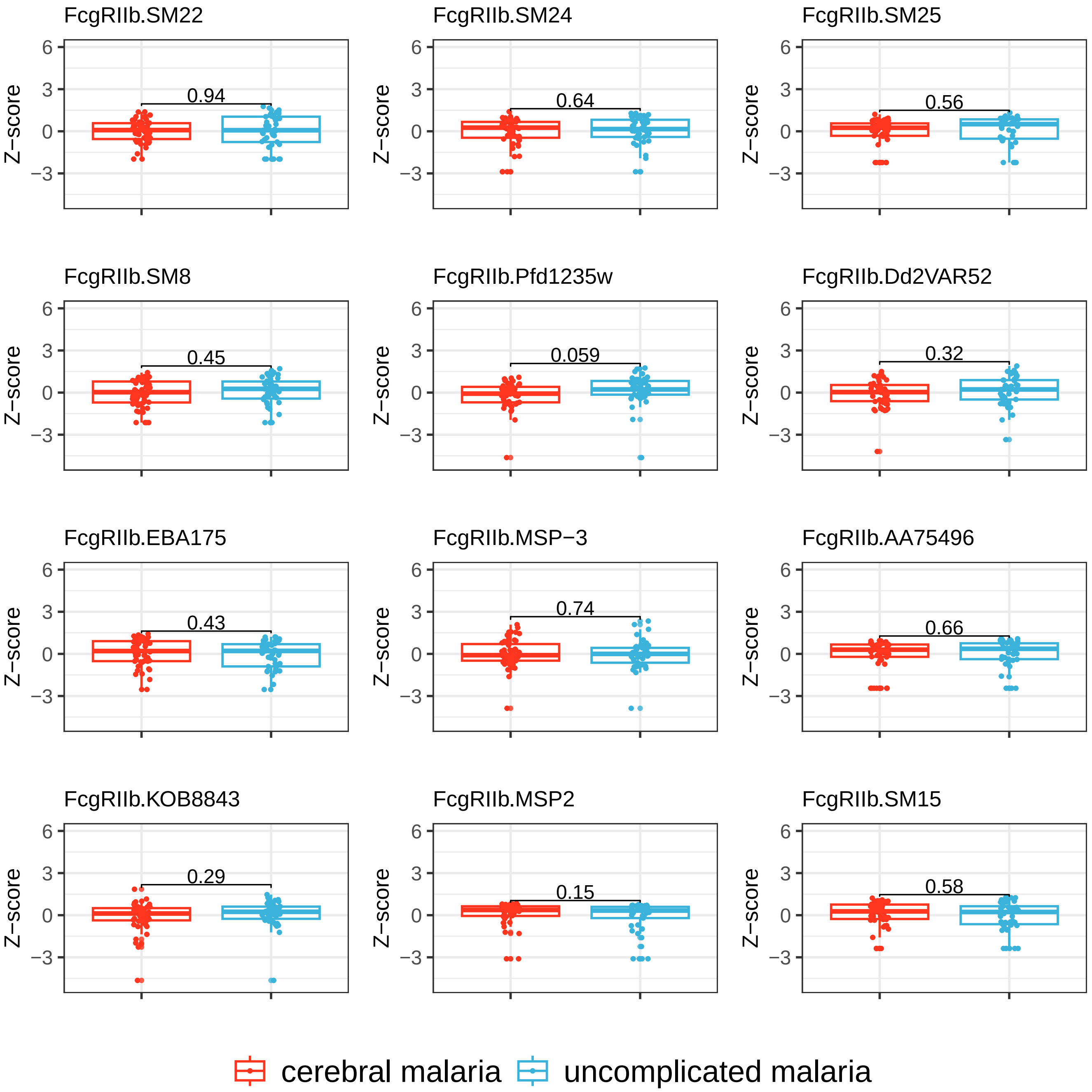

FcgRIIb.SM22
0.94
FcgRIIb.SM24
0.64
FcgRIIb.SM25
0.56
FcgRIIb.SM8
0.45
FcgRIIb.Pfd1235w
0.059
FcgRIIb.Dd2VAR52
0.32
FcgRIIb.EBA175
0.43
FcgRIIb.MSP−3
0.74
FcgRIIb.AA75496
0.66
FcgRIIb.KOB8843
0.29
FcgRIIb.MSP2
0.15
FcgRIIb.SM15
0.58
Z−score
6
3
0
−3
cerebral malaria
uncomplicated malaria

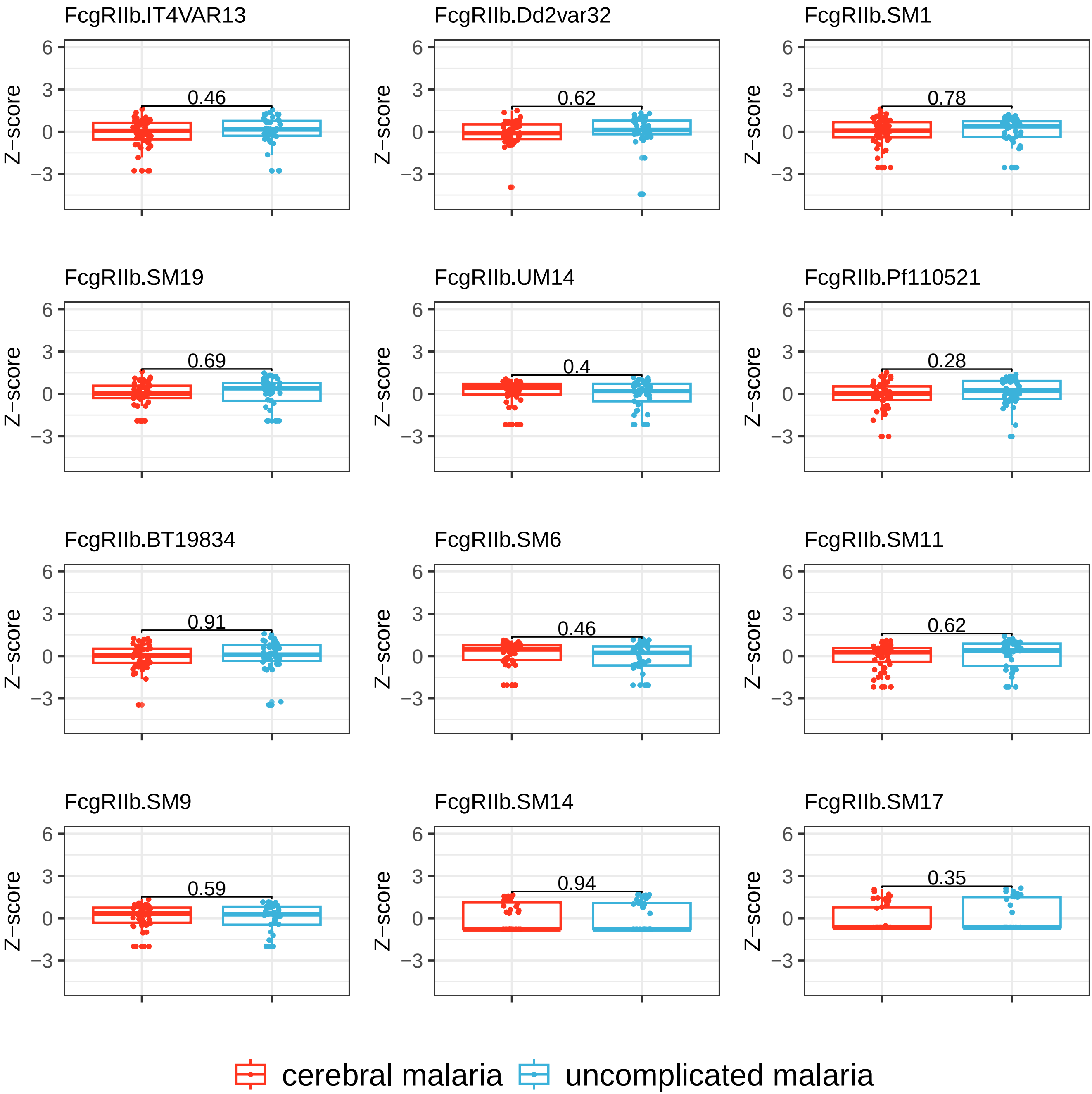

FcgRIIb.IT4VAR13
0.46
FcgRIIb.Dd2var32
0.62
FcgRIIb.SM1
0.78
FcgRIIb.SM19
0.69
FcgRIIb.UM14
0.4
FcgRIIb.Pf110521
0.28
FcgRIIb.BT19834
0.91
FcgRIIb.SM6
0.46
FcgRIIb.SM11
0.62
FcgRIIb.SM9
0.59
FcgRIIb.SM14
0.94
FcgRIIb.SM17
0.35
Z−score
6
3
0
−3
cerebral malaria
uncomplicated malaria

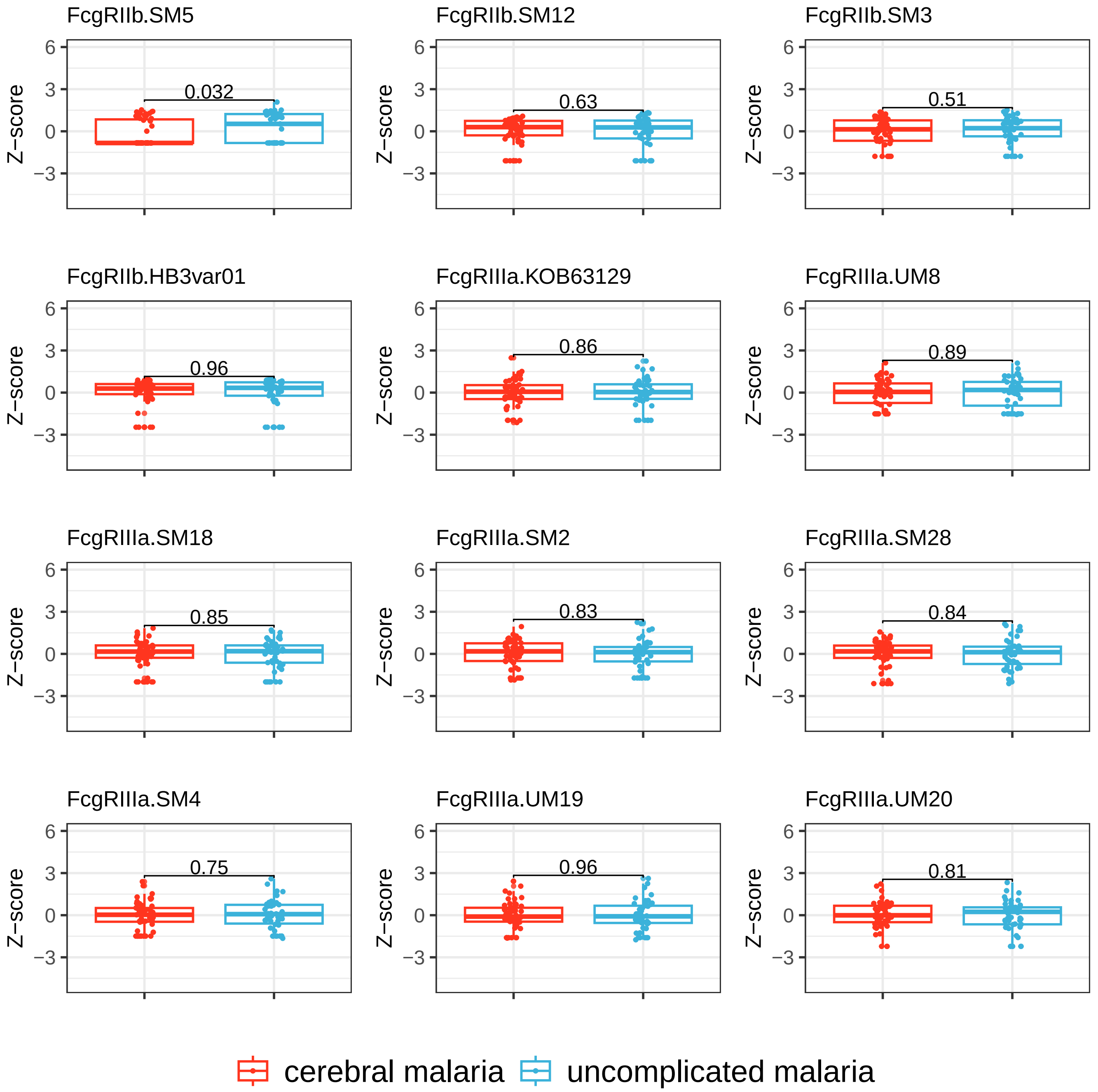

FcgRIIb.SM5
0.032
FcgRIIb.SM12
0.63
FcgRIIb.SM3
0.51
FcgRIIb.HB3var01
0.96
FcgRIIIa.KOB63129
0.86
FcgRIIIa.UM8
0.89
FcgRIIIa.SM18
0.85
FcgRIIIa.SM2
0.83
FcgRIIIa.SM28
0.84
FcgRIIIa.SM4
0.75
FcgRIIIa.UM19
0.96
FcgRIIIa.UM20
0.81
Z−score
6
3
0
−3
cerebral malaria
uncomplicated malaria

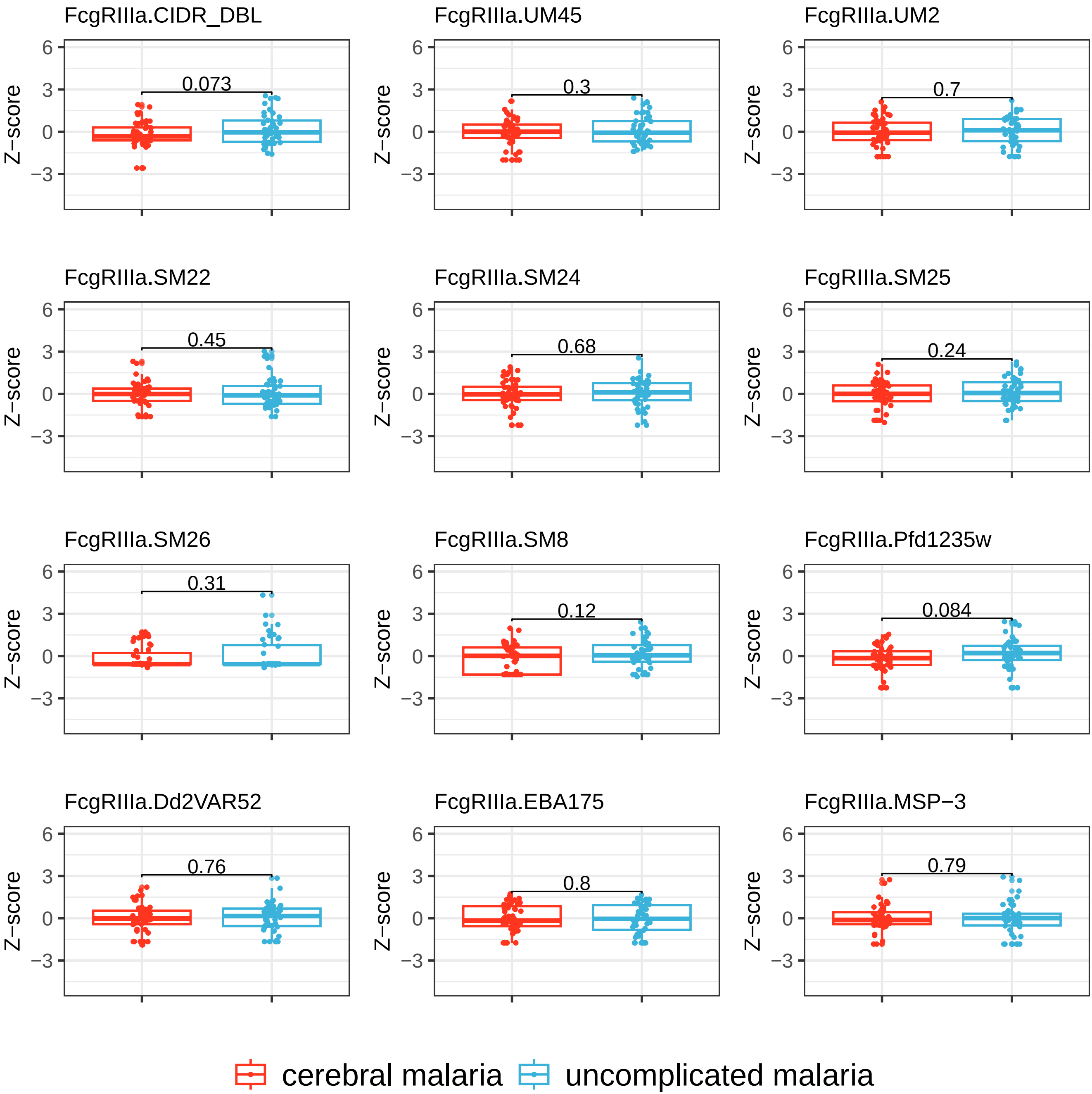
FcgRIIIa.CIDR_DBL
0.073
FcgRIIIa.UM45
0.3
FcgRIIIa.UM2
0.7
FcgRIIIa.SM22
0.45
FcgRIIIa.SM24
0.68
FcgRIIIa.SM25
0.24
FcgRIIIa.SM26
0.31
FcgRIIIa.SM8
0.12
FcgRIIIa.Pfd1235w
0.084
FcgRIIIa.Dd2VAR52
0.76
FcgRIIIa.EBA175
0.8
FcgRIIIa.MSP−3
0.79
Z−score
6
3
0
−3
cerebral malaria
uncomplicated malaria

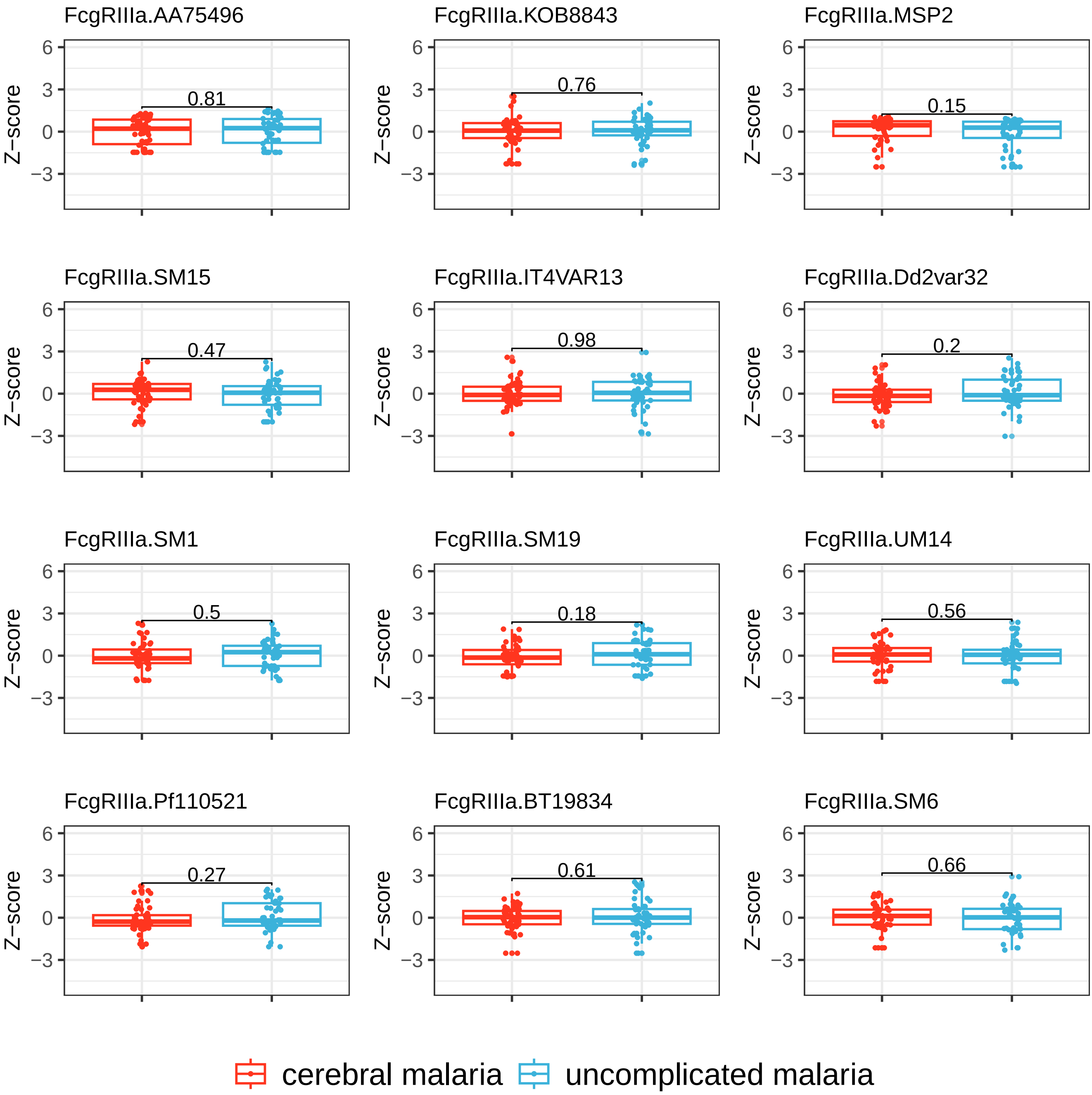

FcgRIIIa.AA75496
0.81
FcgRIIIa.KOB8843
0.76
FcgRIIIa.MSP2
0.15
FcgRIIIa.SM15
0.47
FcgRIIIa.IT4VAR13
0.98
FcgRIIIa.Dd2var32
0.2
FcgRIIIa.SM1
0.5
FcgRIIIa.SM19
0.18
FcgRIIIa.UM14
0.56
FcgRIIIa.Pf110521
0.27
FcgRIIIa.BT19834
0.61
FcgRIIIa.SM6
0.66
Z−score
6
3
0
−3
cerebral malaria
uncomplicated malaria

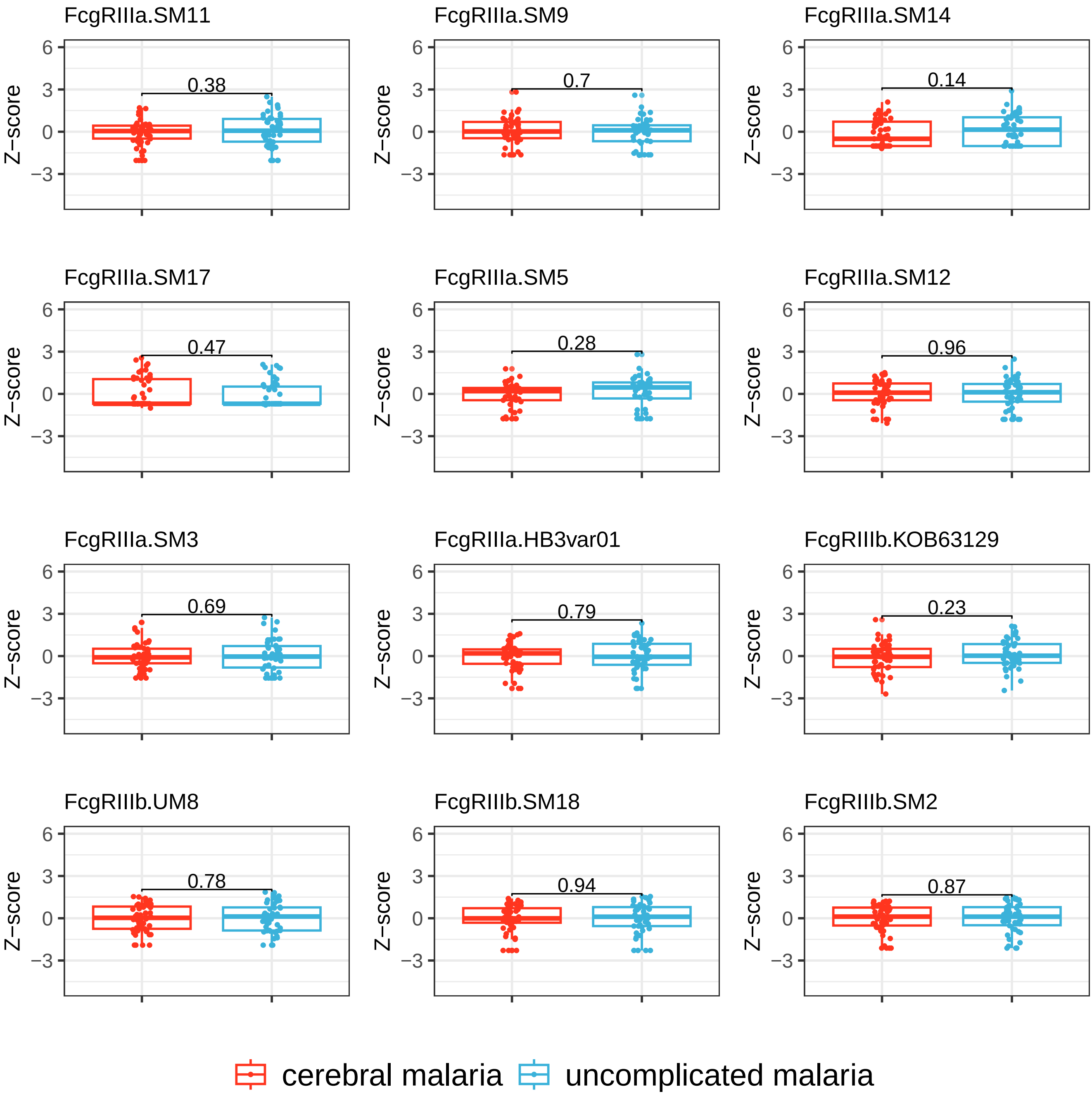
FcgRIIIa.SM11
0.38
FcgRIIIa.SM9
0.7
FcgRIIIa.SM14
0.14
FcgRIIIa.SM17
0.47
FcgRIIIa.SM5
0.28
FcgRIIIa.SM12
0.96
FcgRIIIa.SM3
0.69
FcgRIIIa.HB3var01
0.79
FcgRIIIb.KOB63129
0.23
FcgRIIIb.UM8
0.78
FcgRIIIb.SM18
0.94
FcgRIIIb.SM2
0.87
Z−score
6
3
0
−3
cerebral malaria
uncomplicated malaria

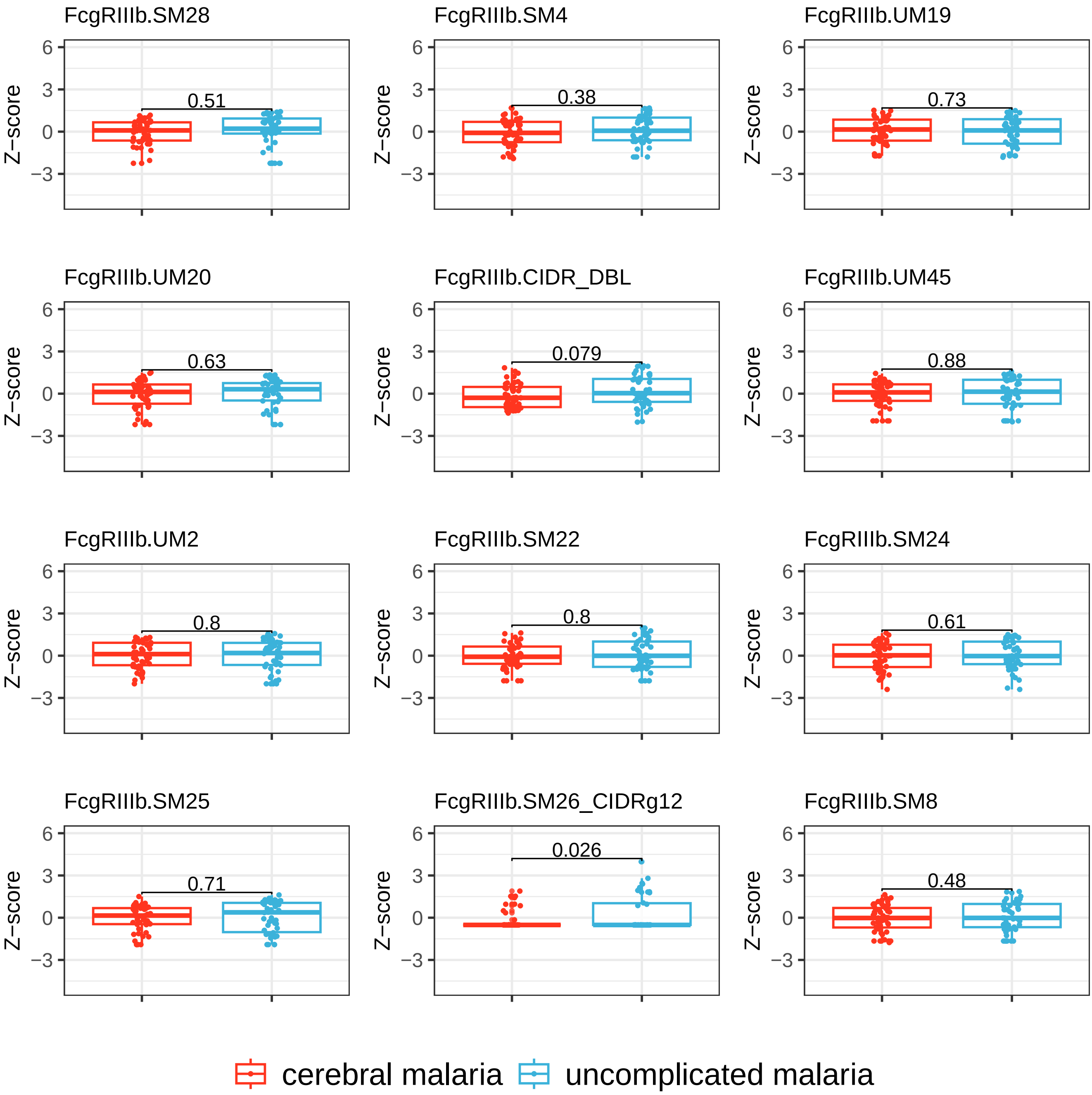
FcgRIIIb.SM28
0.51
FcgRIIIb.SM4
0.38
FcgRIIIb.UM19
0.73
FcgRIIIb.UM20
0.63
FcgRIIIb.CIDR_DBL
0.079
FcgRIIIb.UM45
0.88
FcgRIIIb.UM2
0.8
FcgRIIIb.SM22
0.8
FcgRIIIb.SM24
0.61
FcgRIIIb.SM25
0.71
FcgRIIIb.SM26_CIDRg12
0.026
FcgRIIIb.SM8
0.48
Z−score
6
3
0
−3
cerebral malaria
uncomplicated malaria

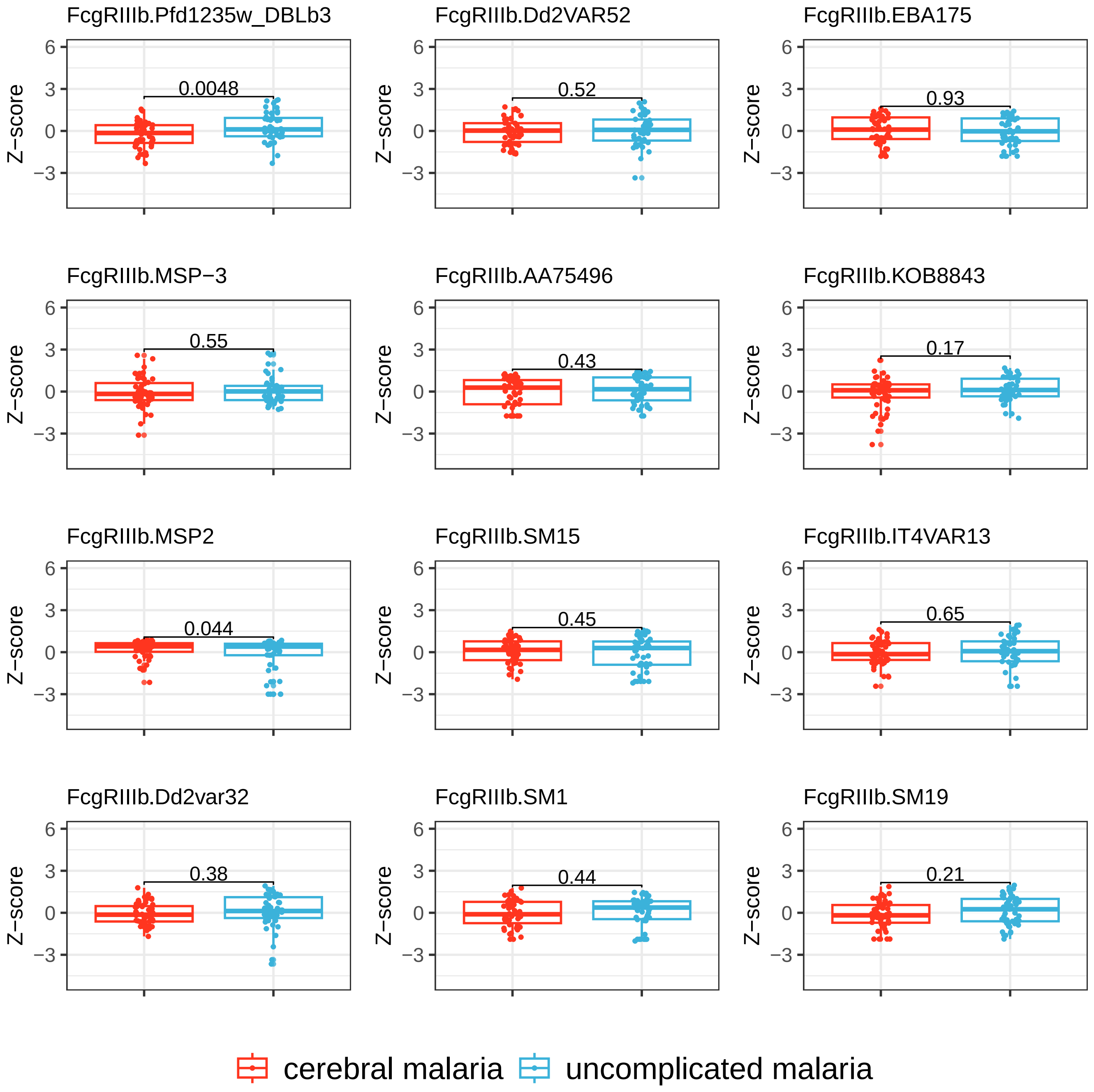
FcgRIIIb.Pfd1235w_DBLb3
0.0048
FcgRIIIb.Dd2VAR52
0.52
FcgRIIIb.EBA175
0.93
FcgRIIIb.MSP−3
0.55
FcgRIIIb.AA75496
0.43
FcgRIIIb.KOB8843
0.17
FcgRIIIb.MSP2
0.044
FcgRIIIb.SM15
0.45
FcgRIIIb.IT4VAR13
0.65
FcgRIIIb.Dd2var32
0.38
FcgRIIIb.SM1
0.44
FcgRIIIb.SM19
0.21
Z−score
6
3
0
−3
cerebral malaria
uncomplicated malaria

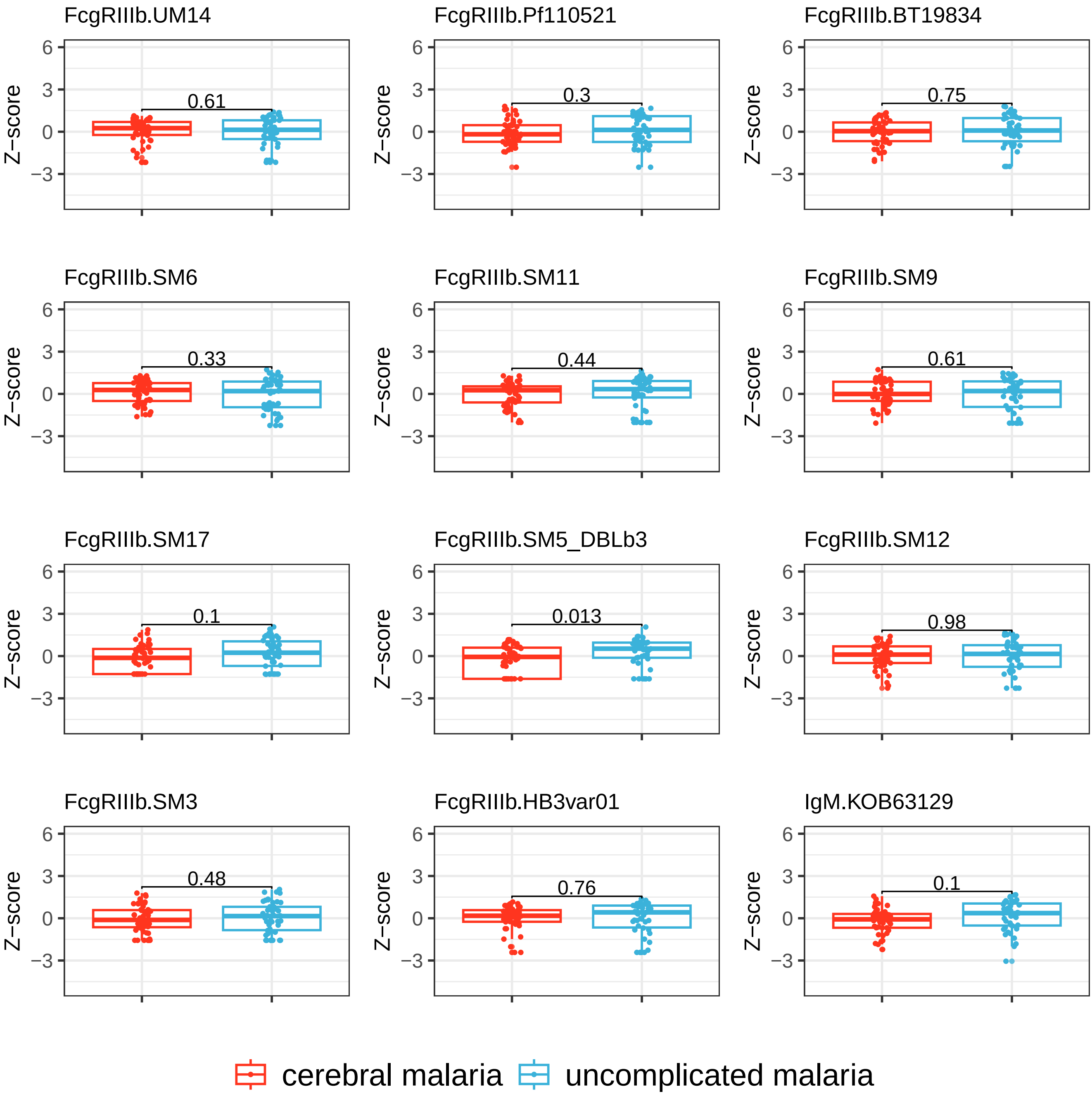
FcgRIIIb.UM14
0.61
FcgRIIIb.Pf110521
0.3
FcgRIIIb.BT19834
0.75
FcgRIIIb.SM6
0.33
FcgRIIIb.SM11
0.44
FcgRIIIb.SM9
0.61
FcgRIIIb.SM17
0.1
FcgRIIIb.SM5_DBLb3
0.013
FcgRIIIb.SM12
0.98
FcgRIIIb.SM3
0.48
FcgRIIIb.HB3var01
0.76
IgM.KOB63129
0.1
Z−score
6
3
0
−3
cerebral malaria
uncomplicated malaria

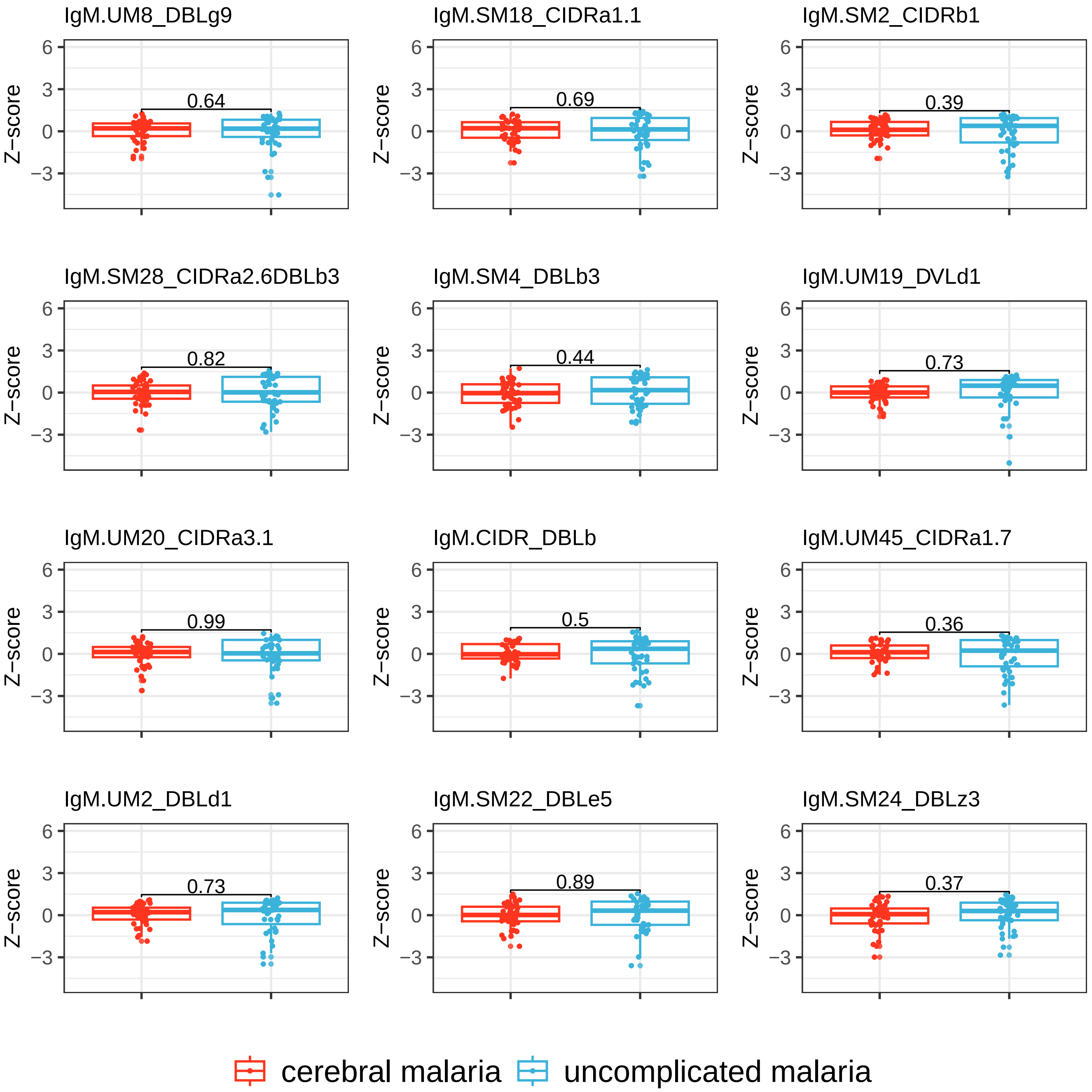
IgM.UM8_DBLg9
0.64
IgM.SM18_CIDRa1.1
0.69
IgM.SM2_CIDRb1
0.39
IgM.SM28_CIDRa2.6DBLb3
0.82
IgM.SM4_DBLb3
0.44
IgM.UM19_DVLd1
0.73
IgM.UM20_CIDRa3.1
0.99
IgM.CIDR_DBLb
0.5
IgM.UM45_CIDRa1.7
0.36
IgM.UM2_DBLd1
0.73
IgM.SM22_DBLe5
0.89
IgM.SM24_DBLz3
0.37
Z−score
6
3
0
−3
cerebral malaria
uncomplicated malaria

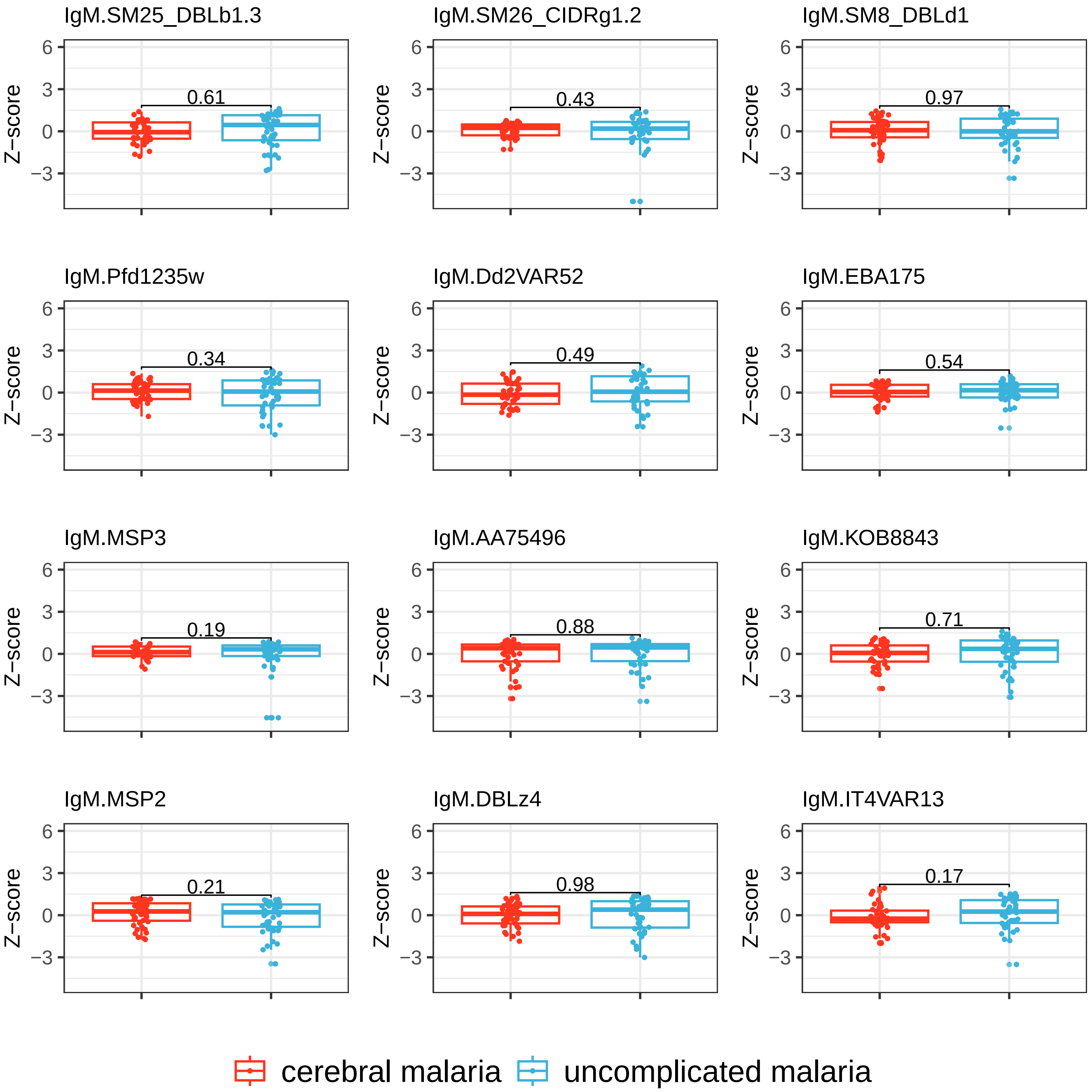

IgM.SM25_DBLb1.3
0.61
IgM.SM26_CIDRg1.2
0.43
IgM.SM8_DBLd1
0.97
IgM.Pfd1235w
0.34
IgM.Dd2VAR52
0.49
IgM.EBA175
0.54
IgM.MSP3
0.19
IgM.AA75496
0.88
IgM.KOB8843
0.71
IgM.MSP2
0.21
IgM.DBLz4
0.98
IgM.IT4VAR13
0.17
Z−score
6
3
0
−3
cerebral malaria
uncomplicated malaria

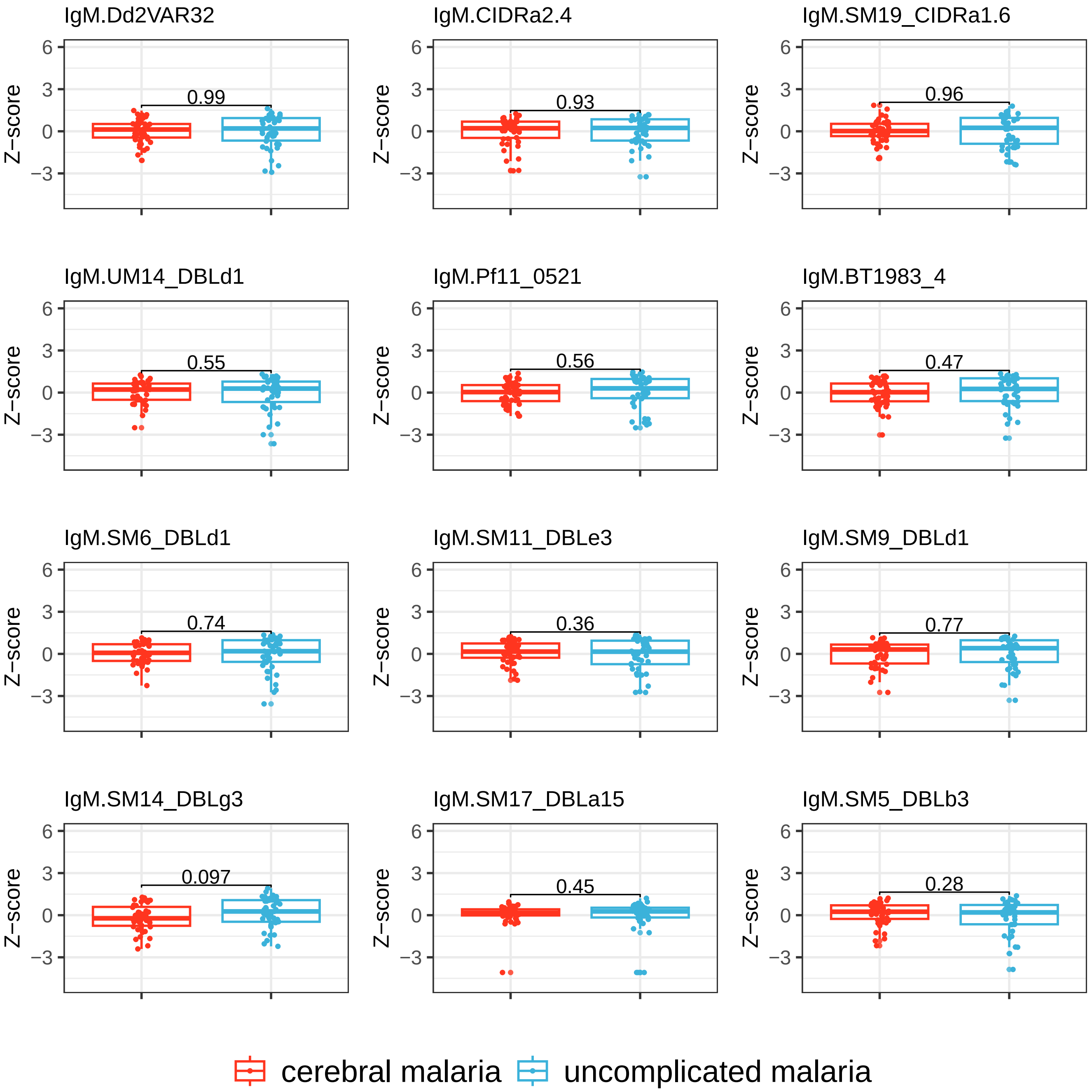
IgM.Dd2VAR32
0.99
IgM.CIDRa2.4
0.93
IgM.SM19_CIDRa1.6
0.96
IgM.UM14_DBLd1
0.55
IgM.Pf11_0521
0.56
IgM.BT1983_4
0.47
IgM.SM6_DBLd1
0.74
IgM.SM11_DBLe3
0.36
IgM.SM9_DBLd1
0.77
IgM.SM14_DBLg3
0.097
IgM.SM17_DBLa15
0.45
IgM.SM5_DBLb3
0.28
Z−score
6
3
0
−3
cerebral malaria
uncomplicated malaria

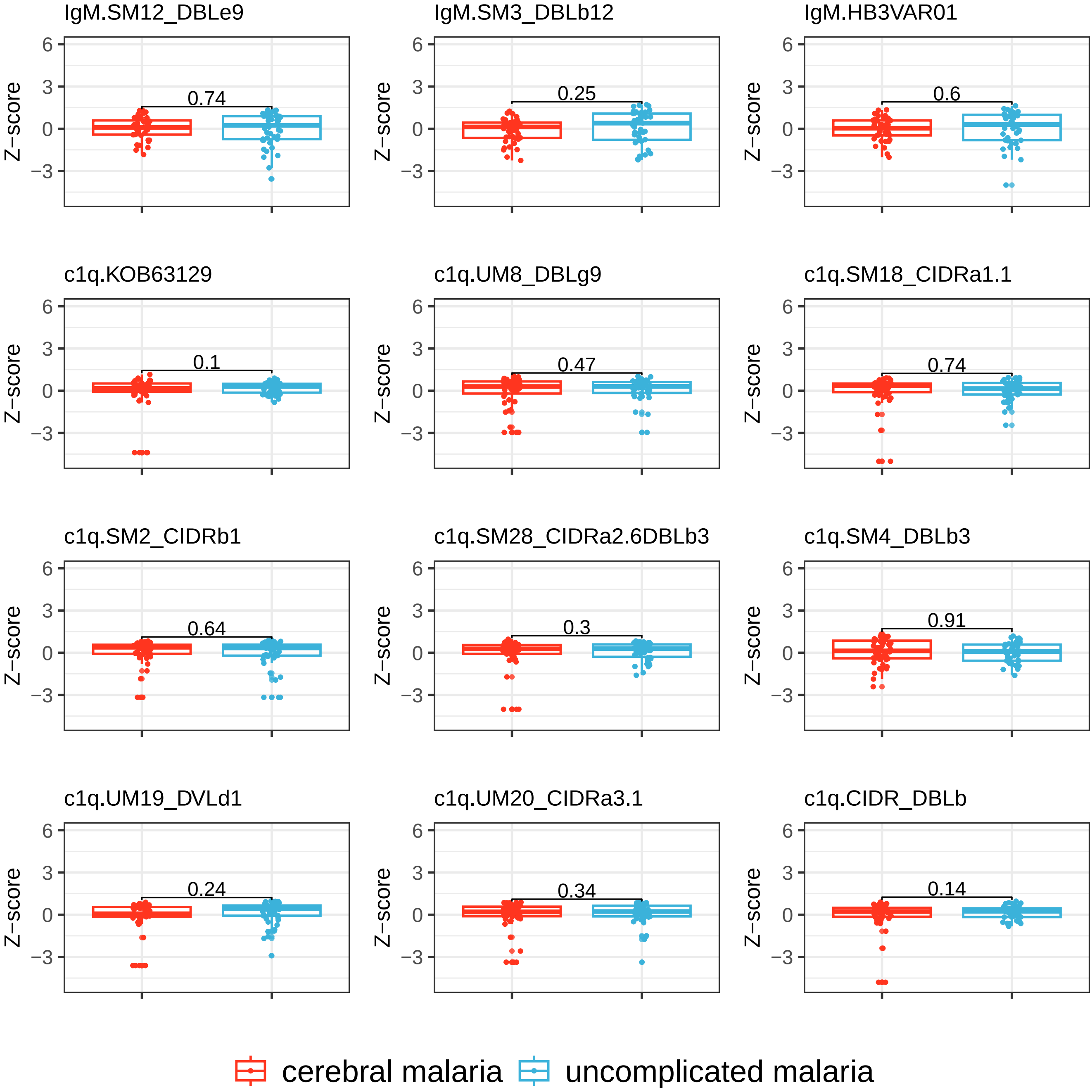
IgM.SM12_DBLe9
0.74
IgM.SM3_DBLb12
0.25
IgM.HB3VAR01
0.6
c1q.KOB63129
0.1
c1q.UM8_DBLg9
0.47
c1q.SM18_CIDRa1.1
0.74
c1q.SM2_CIDRb1
0.64
c1q.SM28_CIDRa2.6DBLb3
0.3
c1q.SM4_DBLb3
0.91
c1q.UM19_DVLd1
0.24
c1q.UM20_CIDRa3.1
0.34
c1q.CIDR_DBLb
0.14
Z−score
6
3
0
−3
cerebral malaria
uncomplicated malaria

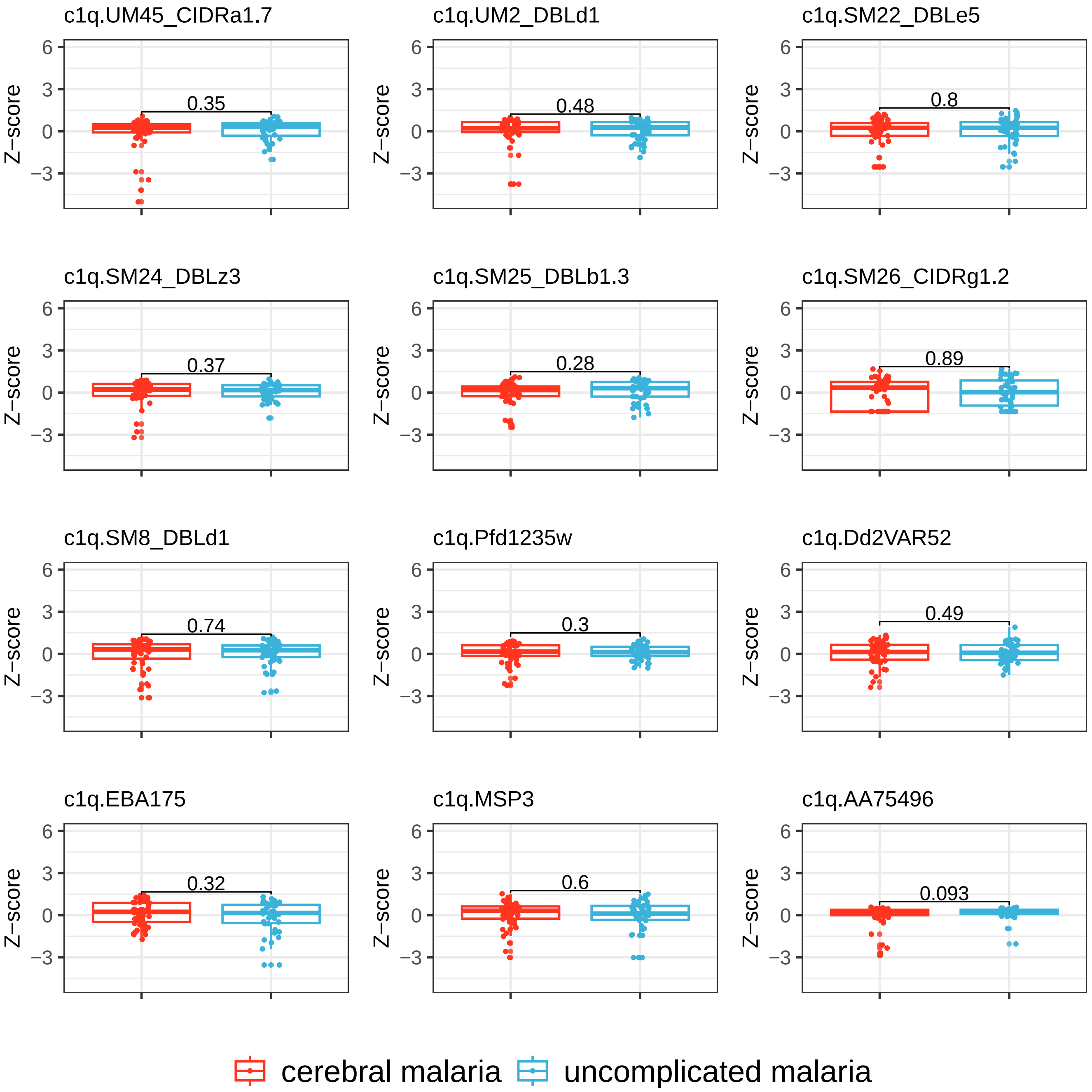
c1q.UM45_CIDRa1.7
0.35
c1q.UM2_DBLd1
0.48
c1q.SM22_DBLe5
0.8
c1q.SM24_DBLz3
0.37
c1q.SM25_DBLb1.3
0.28
c1q.SM26_CIDRg1.2
0.89
c1q.SM8_DBLd1
0.74
c1q.Pfd1235w
0.3
c1q.Dd2VAR52
0.49
c1q.EBA175
0.32
c1q.MSP3
0.6
c1q.AA75496
0.093
Z−score
6
3
0
−3
cerebral malaria
uncomplicated malaria

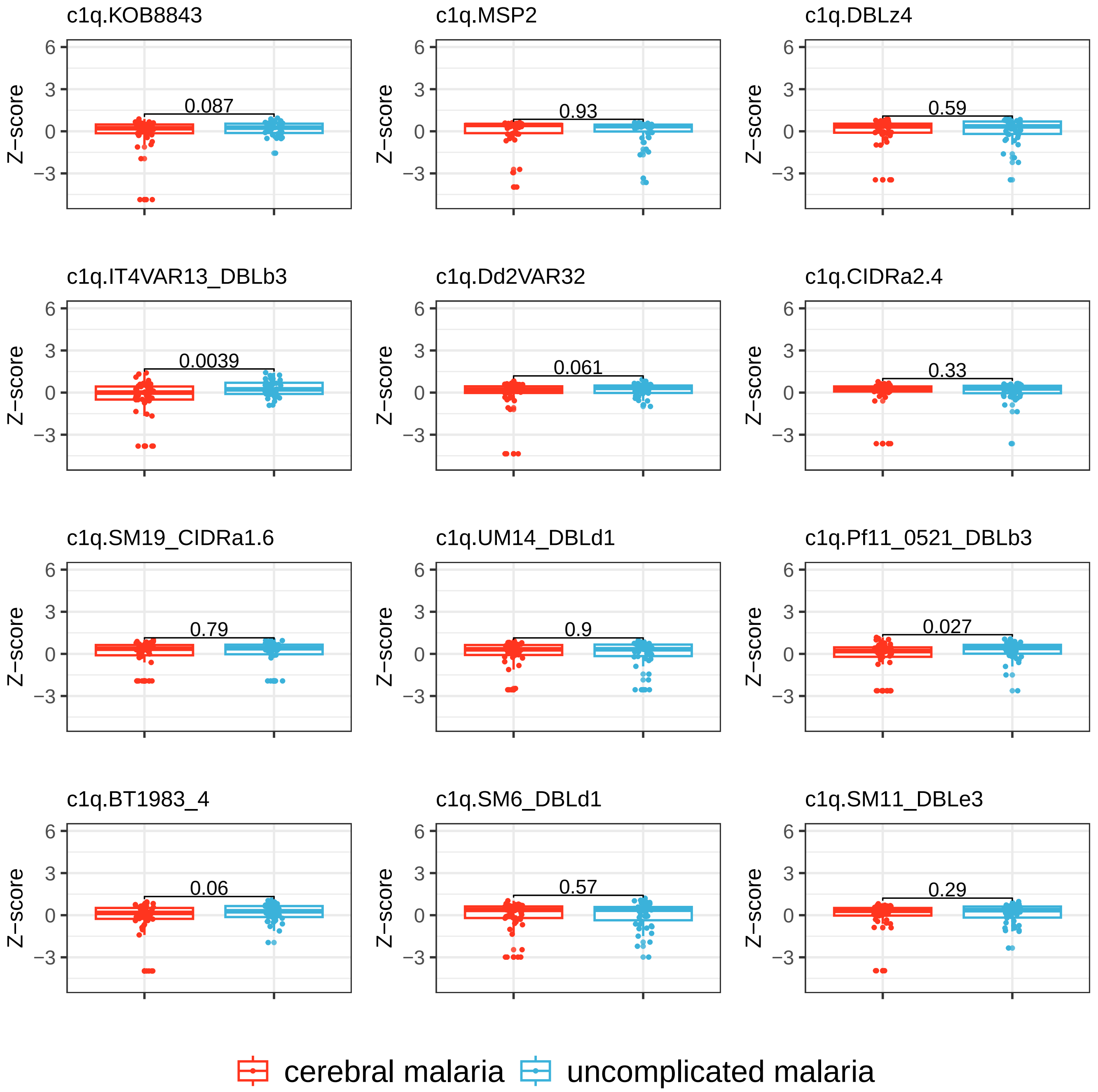

c1q.KOB8843
0.087
c1q.MSP2
0.93
c1q.DBLz4
0.59
c1q.IT4VAR13_DBLb3
0.0039
c1q.Dd2VAR32
0.061
c1q.CIDRa2.4
0.33
c1q.SM19_CIDRa1.6
0.79
c1q.UM14_DBLd1
0.9
c1q.Pf11_0521_DBLb3
0.027
c1q.BT1983_4
0.06
c1q.SM6_DBLd1
0.57
c1q.SM11_DBLe3
0.29
Z−score
6
3
0
−3
cerebral malaria
uncomplicated malaria

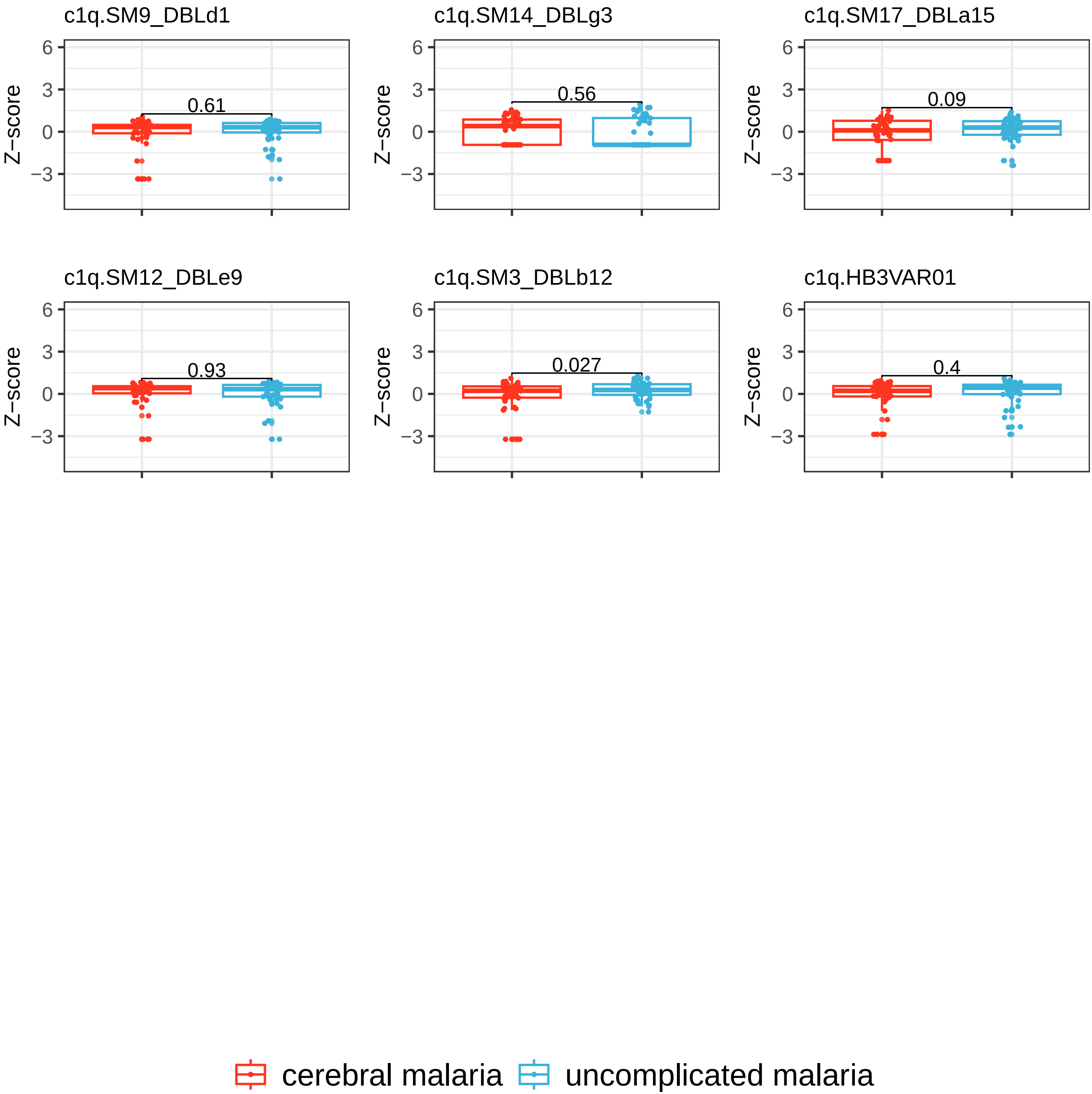
c1q.SM9_DBLd1
c1q.SM14_DBLg3
c1q.SM17_DBLa15
c1q.SM12_DBLe9
c1q.SM3_DBLb12
c1q.HB3VAR01
Z−score
0.61
0.56
0.09
0.93
0.027
0.4
6
3
0
−3
cerebral malaria
uncomplicated malaria
